# Automated annotation of complex natural products using a modular fragmentation–based structure assembly (MFSA) strategy

**DOI:** 10.1126/sciadv.adw4693

**Published:** 2025-08-15

**Authors:** Mi Zhang, Kouharu Otsuki, Lingjian Tan, Takashi Kikuchi, Ning Li, Wei Li

**Affiliations:** ^1^ Faculty of Pharmaceutical Sciences, Toho University, Miyama 2-2-1, Funabashi, Chiba 274-8510, Japan.; ^2^ School of Traditional Chinese Materia Medica, Key Laboratory of Innovative Traditional Chinese Medicine for Major Chronic Diseases of Liaoning province, Key Laboratory for TCM Material Basis Study and Innovative Drug Development of Shenyang City, Shenyang Pharmaceutical University, Shenyang 110016, China.

## Abstract

Complex natural products (CNPs) feature polycyclic structures, multiple consecutive chiral centers, and nonrepetitive structural units. Given their complexity, the structural annotation of CNPs remains a major bottleneck. Here, we introduce the modular fragmentation–based structural assembly (MFSA) strategy for the target CNP structural annotation. The MFSA strategy disassembled the structure based on fragmentation patterns, recognized targets via a pseudo-library, and reassembled the structure using characteristic ions and neutral losses. As a proof of concept, we focused on daphnane-type diterpenoids, a kind of specific bioactive CNP from Thymelaeaceae plants. Furthermore, we present a user-friendly application named CNPs-MFSA coded in Python. Using an in-house daphnane library, CNPs-MFSA outperformed SIRIUS, MS-FINDER, and MetFrag in Top-1 annotation accuracy. By applying CNPs-MFSA to 56 Thymelaeaceae plants, 204 high-confidence daphnanes within 822 annotated results, including 105 previously unreported compounds were found. Aconitine, paclitaxel, and obakunone analogs were incorporated as additional target CNP classes to illustrate the extension workflow.

## Teaser

A modular fragmentation strategy enables automated annotation of target complex natural products via mass spectrometry.

## INTRODUCTION

Natural products (NPs) are granted a privileged status in drug discovery due to their structural properties (*1*, *2*). Nearly half of drugs newly approved by the Food and Drug Administration were NPs or their derivatives (*3*, *4*). Although more than 340,000 NPs have been recorded in the Dictionary of Natural Products, the vast majority remains insufficiently elucidated and underused (*5*, *6*). Complex natural products (CNPs) are characterized by polycyclic structures, abundant stereochemistry throughout the sp^3^-hybridized carbon atom skeleton, and nonrepetitive structural units, unlike lipids or peptides (*7*). Many CNPs exhibit substantial bioactivity and provide a unique source of previously uncharacterized scaffolds with unprecedented modes of action. In natural extracts, the complexity arises from the large variety of CNPs present, each in varying concentrations. This complexity makes elucidating the components of natural extracts quite challenging. Conventional techniques such as nuclear magnetic resonance (NMR) spectral analysis and x-ray crystallography have been widely used for structural elucidation (*8*). These methods require milligram-scale quantities of pure compounds or crystals obtained through isolation and purification processes. Although they offer the advantage of distinguishing isomers and determining stereochemistry, they are often time-consuming and labor-intensive.

Advances in analytical techniques have changed the landscape of CNP discovery. Liquid chromatography coupled to mass spectrometry (LC-MS) is one of the most widely used analytical methods, and it enables rapid, high-throughput screening for measuring thousands of metabolic features with a simple sample preparation, even for small quantities of natural extracts (*9*). With its high sensitivity and applicability to diverse molecules, MS serves as a basis for rapid structural elucidation. The nontargeted experiment focuses on analyzing all the components in specific complex mixtures. In general, the structural annotation of nontargeted experiments depends on matching experimental tandem mass spectrometry (MS/MS) spectra against public databases or in silico databases (e.g., ISDB) (*10*). Alternatively, molecular networking strategies such as Global Natural Products Social Molecular Networking (GNPS) based on dot product (cosine) similarity algorithms have gained considerable attention, which comprehensively and nonselectively calculates and clusters all metabolites depending on product ion spectra similarity and various in silico approaches [e.g., NAP (*11*), MS2LDA (*12*), and MolNetEnhancer (*13*)] were developed, facilitating further structural annotation (*14*). In addition, recent studies have incorporated machine learning for compound annotation; for example, LC-MS^2^Struct annotates small molecules using retention order and product ion spectra (*15*) and DeepCDM predicts MS/MS data using transfer learning for dansylated molecules (*16*). Despite the challenges of identifying structure through MS, such as the difficulty in differentiating structural isomers and resolving stereochemistry, these techniques facilitate high-throughput analysis by allowing rapid data acquisition from batches of samples.

Targeted profiling experiments for discovering specific bioactive CNPs have rarely been reported (*17*), but such experiments are a crucial and time-limiting step in rapid dereplication and target isolation. Several challenges have arisen in target experiments for CNP annotation using the general database matching described above or network-based approaches. Although many public databases containing NPs exist [e.g., MassIVE (*14*), AntiBase (*18*), FooDB (*19*), NIST (*20*), and MassBank (*21*)], most databases have high content redundancy and inconsistently acquired data. The publicly available experimental MS data for structural annotation covers less than 5% of the reported NPs, and this limits the scope of the molecules that can be accurately identified (*22*). Various in silico MS/MS prediction tools, such as CFM-ID (*23*), MetFrag (*24*), MS-FINDER (*25*), and SIRIUS (*26*), also exhibit low accuracy due to the structural complexity of the CNPs. Similarly, the limited availability of training data hinders the construction of highly accurate models for machine learning–based structural annotation (*27*). Moreover, CNPs having similar skeletons may exhibit low spectral similarity due to their diverse oxidation patterns, even between related analogs, and complete clustering using molecular networking strategies remains challenging (*17*). Extensive manual elucidation and experimental MS data are still needed for effective annotation.

**Fig. 1.**
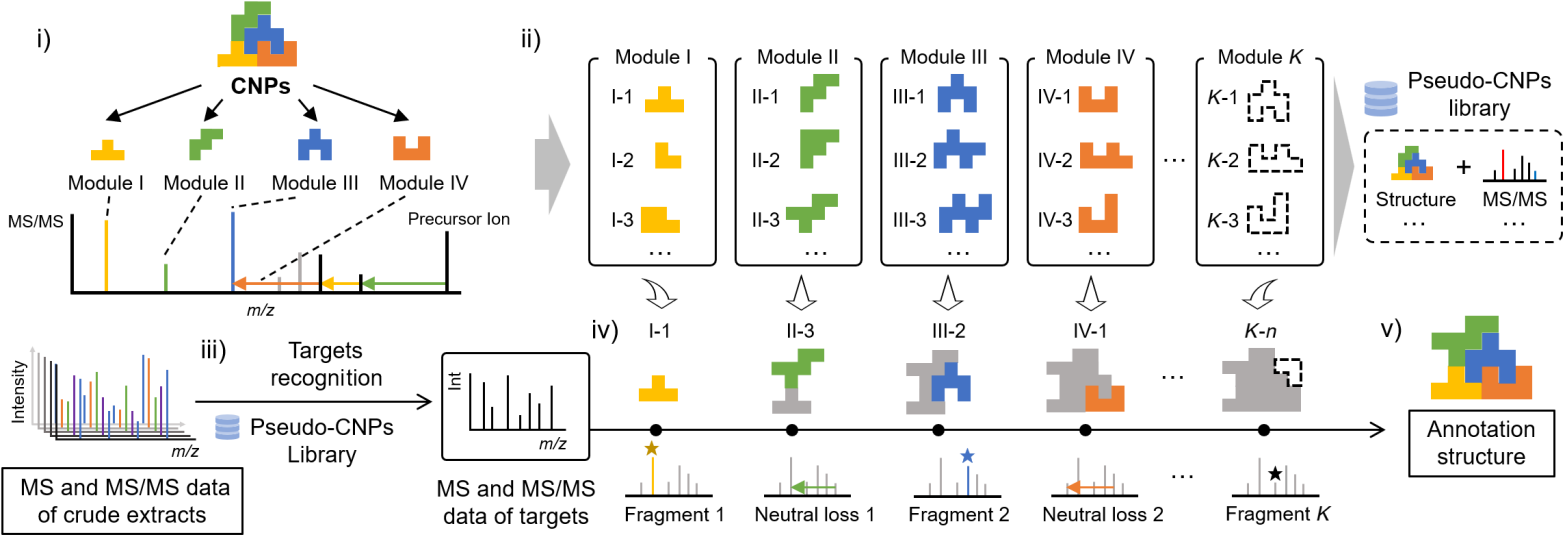
MFSA strategy for specific CNP target annotation. (**i**) Disassembling the target CNP structure into modules. (**ii**) Building a pseudo-CNP library. (**iii**) Recognizing the target CNP using the pseudo-CNP library. (**iv**) Annotating the module structures of the CNP. (**v**) Reassembling the annotated modules.

In this work, we propose a modular fragmentation–based structural assembly (MFSA) strategy in a target experiment for specific CNP structural annotation (Fig. 1). The MFSA strategy involves (i) disassembling the target CNP structure into modules based on fragmentation patterns, (ii) building a pseudo-CNP library encompassing all currently reported compounds, (iii) recognizing the target CNP using the characteristic product ion formulae predicted in the pseudo-CNP library, (iv) annotating the module structures of the CNP by labeling the characteristic product ions and neutral losses, and (v) reassembling the annotated modules to generate possible annotated candidates. The MFSA strategy enables breaking through the known chemical boundaries, covering all possible structures of the CNPs currently reported, predicting MS fragments with high structural dependence, and providing comprehensive recognition and annotation of targets in complex mixtures.

As the main proof of concept, we aim at daphnane-type diterpenoids, a kind of specific CNP from Thymelaeaceae plants, with a trans-fused 5/7/6-tricyclic ring system containing at least seven contiguous chiral centers; these are characterized by highly oxidized functionalities or macrocyclic rings spanning their skeleton. Approximately 215 daphnanes have been isolated from Thymelaeaceae plants as of March 2025 (*28*, *29*). Notably, several daphnanes, such as resiniferatoxin and gnidimacrin, have shown remarkable biological activities, including anticancer (*30*), anti-HIV (*31*), and analgesic effects (*32*), and have garnered notable attention in drug discovery research. The limited availability of the mass spectra of daphnanes in public databases was another reason a case study was conducted (*33*).

With the conception of the MFSA strategy, we developed a Python-based automated annotation application named CNPs-MFSA for the structural annotation of specific CNPs. CNPs-MFSA was benchmarked against SIRIUS, MS-FINDER, and MetFrag (integrated with a custom daphnane library) for the annotation of 58 in-house daphnane-type diterpenoids. The CNPs-MFSA application achieved the annotation of 822 daphnanes across 56 plant extracts from the Thymelaeaceae family. Furthermore, aconitine-type alkaloids, taxane-type diterpenoids, and A,D-*seco* limonoid-type triterpenoids, each of which are notable for their structural complexity and potent biological activity, were included as additional CNP classes to illustrate the extension workflow in selected experiments.

## RESULTS

### MFSA strategy for CNPs

#### Module design for CNPs

*Design principles.* NPs within the same structural class typically share similar carbon skeletons or substructures. Structurally related CNPs tend to undergo fragmentation at similar cleavage sites and exhibit comparable MS/MS fragmentation patterns driven by analogous fragmentation reaction mechanisms. Therefore, MS/MS fragmentation–based module design adheres to three key principles:

1) Common fragmentation sites: Same-class CNPs are disassembled into modules through the identification of common or similar fragmentation sites observed across the class, which serve as logical boundaries for defining reusable modules.

2) Ion- and loss-based modular definition: Substructures corresponding to a single reproducible diagnostic product ion or a well-defined group of such ions, characteristic neutral losses, or in-source fragments are defined as individual modules, which ensures a direct correlation between the structure and the MS features.

3) Fragmentation robustness: Acceptable variations within each module (e.g., alkyl substitutions and hydroxylation) are limited to those that do not markedly change the characteristic fragmentation behavior of the overall molecule; this ensures that the module remains robust and generalizable within the CNP class.

*Practical heuristics for module design.* To translate the above principles into practical workflows, the following heuristics are proposed: (i) Module boundaries are preferentially defined at fragmentation sites that are consistently observed across structurally related compounds. These sites typically correspond to unstable C─O bonds (e.g., ether, ester, or glycosidic linkages) or electronically favorable cleavage positions (e.g., α-cleavage adjacent to carbonyls or double bonds). These conserved fragmentation sites generate high-intensity and structure-informative product ions, which serve as reliable indicators of modular disassembly; (ii) variations within a module, such as the alkyl chain length, hydroxylation, or methoxylation, are allowed, provided that such modifications have no effect on the primary fragmentation behavior of the parent molecule; (iii) to maintain annotation efficiency, overfragmentation into excessively small structural units should be avoided unless the submodules generate unique and reproducible diagnostic ions; (iv) module construction follows a hierarchical logic, starting from larger core units (e.g., entire rings or aglycone motifs) and subdividing into smaller units only when necessary to improve classification or specificity; and (v) the selection and validation of modules is guided by biosynthetic logic, phytochemical literature, and known substructures as reported in NP databases.

*Possible generalization strategies.* To facilitate application of the MFSA strategy to other CNP families, we propose the following approaches: (i) For any CNP class, a small representative set of structures, preferably selected from compounds that occur with high abundance or high frequency in nature, can be analyzed to define initial module templates based on shared structural cores and fragmentation features; (ii) fragmentation behavior can be learned from the clustering of product ion spectra according to shared product ions or neutral losses as this enables the data-driven annotation of fragmentation motifs; (iii) frequent substructures in large-scale NP databases (e.g., sugar moieties, prenyl groups, and aromatic units) can serve as candidate transferable modules across different compound families; (iv) a reusable module-product ion library can be constructed using standardized formats [e.g., Simplified Molecular Input Line Entry System (SMILES) for structures and mass/charge ratio (*m/z*) values or element compositions for diagnostic ions], and this would facilitate adaptation to any NP class by matching known submodules; and (v) as for CNPs with fused or bridged ring systems, larger macromodules are defined according to fragmentation coherence rather than biosynthetic units alone.

#### Pseudo-CNP library construction

*Pseudo-CNP structure generation.* Initially, the structure of the CNP was disassembled at logical cleavage sites derived from its MS/MS fragmentation behavior. However, in many cases, the fragmentation sites were not consistent with the atoms shared between two substructures, such as adjacent rings. To enable a more efficient and accurate reconstruction of the structures, the atoms shared between adjacent rings during modular assembly were marked. In particular, shared atoms were labeled within each SMILES fragment, which allowed accurate merging of the substructures. For example, in a tetracyclic scaffold, a molecule may be fragmented into two modules based on its fragmentation patterns. Module I includes a recombined unit composed of rings A and B, whereas module II contains rings C and D. By sequentially assembling these labeled SMILES fragments, combinatorial two-dimensional pseudo-CNP structures are generated.

*Product ion formulae prediction.* To achieve an accurate correspondence between modules and product ions and to improve the precision of the structural annotation, the prediction strategy prioritizes the elemental formulae of the diagnostic product ions over their *m/z* and intensity values. The prediction of the product ion formulae proceeds as follows:

1) Establish a mapping between the modules and fragments: Each structural module was linked to specific MS/MS features based on a fragmentation analysis. For example, module I, which includes the A and C rings of a CNP, corresponds to a diagnostic product ion (ion *i*), whereas module II, which encompasses the B ring, was associated with a characteristic neutral loss (loss *i*). The module-fragment mapping forms the basis for the modular prediction.

2) Setting the initial product ion formula of the module templates: Because CNPs have polycyclic structures and high functionalities, two main types of product ion spectra were commonly observed. In many CNP cases, such as terpenoid cases, product ions originating from the core carbon skeleton appeared with high frequency and intensity. The composition formulae of highly repetitive, high-intensity carbon skeleton-derived product ions were selected as the initial product ion formula for the template modules. The MS/MS spectral data used for constructing module templates were obtained from three main sources: experimentally acquired MS/MS data of authentic reference standards, literature-derived fragmentation information, and public databases (e.g., MassBank, GNPS, etc.). In other cases, such as alkaloids, fragmentation of the carbon skeleton resulted in weaker ion signals, whereas neutral losses from labile substituents (e.g., acetyl, methoxy, and hydroxy groups) were more prominent. In such cases, the precursor ion formula was designated as the initial reference formula for the product ion prediction.

3) The mass shift matrix for submodule definition: The detailed derivation of the mathematical model for product ions prediction was provided in fig. S1 with the main notation given as follows. Supposing the target-CNP was divided into k types of module and defined as $M^{k}$ , each module consisted of j types submodules, defined as $M_{j}^{k}$ . The mass shifts Δm of elements carbon (C), hydrogen (H), oxygen (O), and nitrogen (N), generated by each submodule, were defined as $\Delta m_{M_{j}^{k},C},\Delta m_{M_{j}^{k},H},\Delta m_{M_{j}^{k},O},\text{and}\;\Delta m_{M_{j}^{k},N}$ , respectively. The mass shift matrix for the submodules of each module was defined as $\Delta m_{M_{j}^{k}}$

$$\Delta m_{M_{j}^{k}}=\left(\begin{array}{cccc} \Delta m_{M_{1}^{k},C} & \Delta m_{M_{1}^{k},H} & \Delta m_{M_{1}^{k},O} & \Delta m_{M_{1}^{k},N}\\ \Delta m_{M_{2}^{k},C} & \Delta m_{M_{2}^{k},H} & \Delta m_{M_{2}^{k},O} & \Delta m_{M_{2}^{k},N}\\ \vdots & \vdots & \vdots & \vdots \\ \Delta m_{M_{j}^{k},C} & \Delta m_{M_{j}^{k},H} & \Delta m_{M_{j}^{k},O} & \Delta m_{M_{j}^{k},N} \end{array}\right)$$

4) Prediction product ion formula for any combination: $m_{\text{predict},i}$ is any predicted mass vector $m_{i}$ of the generated product ion formula, $m_{0}$ is the mass vector of the product ion formula of the initial reference compound, and $\Delta m_{M_{i}^{k}}$ is the mass shift vector of any submodule $M_{i}^{k}$

$$m_{\text{predict},i}=m_{0}+\sum_{k=1}^{k}\;\Delta m_{M_{i}^{k}}$$

Both generated module structures represented by SMILES fragments and the characteristic product ions formulas were incorporated using SQLite to generate the pseudo-CNP library for subsequent targeted extraction and structural annotation.

#### CNPs-MFSA workflow

**Fig. 2.**
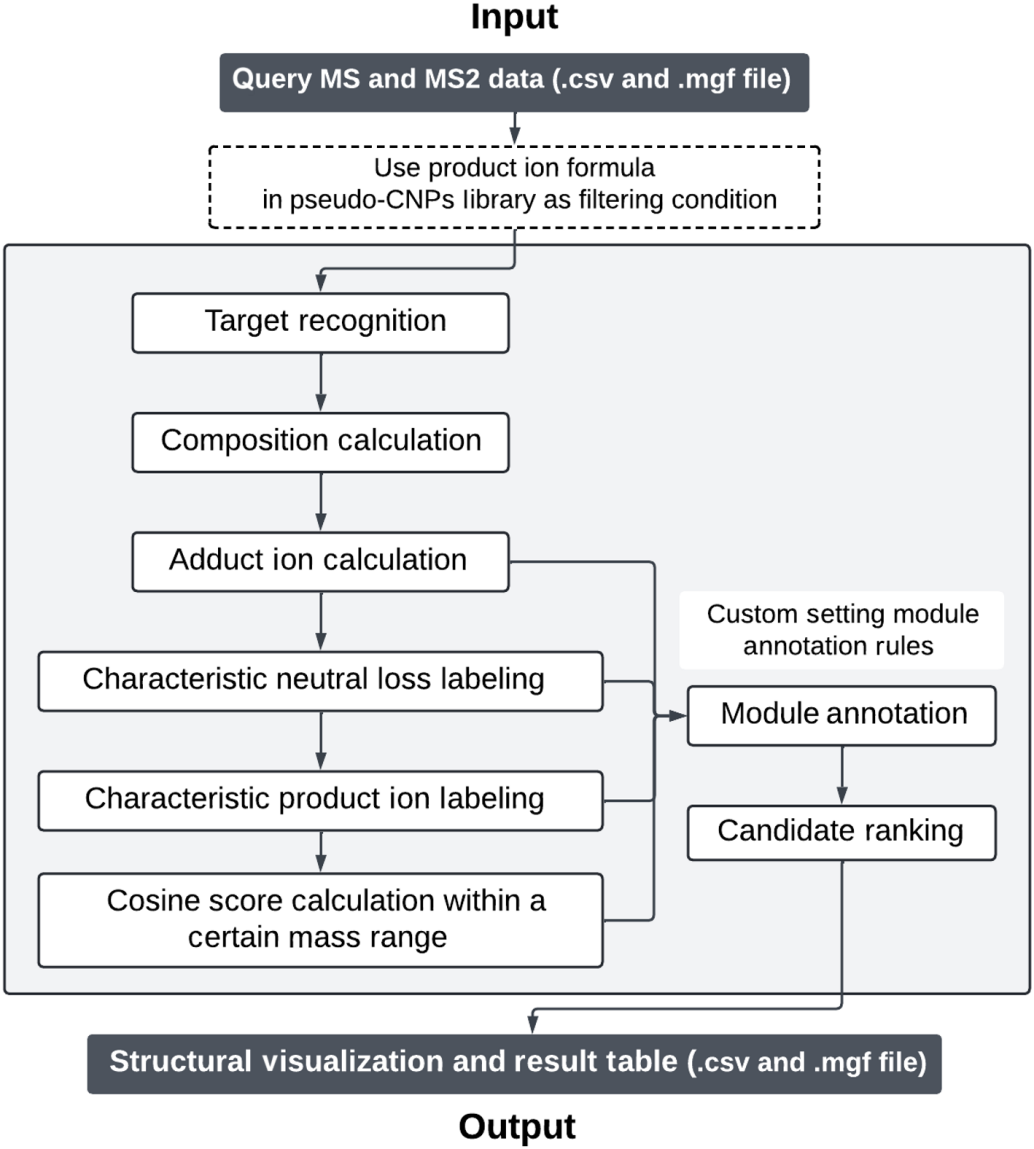
Overview of the CNPs-MFSA structural annotation workflow. The workflow starts with query MS and MS2 data in .csv or .mgf format. Product ion formulas from the pseudo-CNP library are used as filtering conditions for target recognition. The subsequent steps include composition calculation, adduct ion calculation, labeling of characteristic neutral losses and product ions, and cosine score calculation. Users can define custom module annotation rules for module assignment and candidate ranking. The final output includes structural visualizations and result tables in .csv and .mgf formats.

The fragmentation reaction mechanism, diagnostic ions, neutral losses with high structure dependence, and fragmentation site used for substructure annotation can be summarized according to an analysis of product ion spectra. The input files were generated by data preprocessing software such as MZmine (*34*). The workflow of the CNPs-MFSA construction is as follows (Fig. 2).

*Target recognition using the pseudo-CNP library.* The composition formula of the characteristic product ion formulae in the above pseudo-CNP ion library was used as the condition for target extraction and classification. The mass range of each characteristic product ion was calculated to within ±5 parts per million (ppm) when the charge was +1 in the positive-ion mode using the following formula for filtering and matching (*35*)

$$\begin{array}{c} \text{Mass tolerance}\;\left(\text{ppm}\right)=\left|\frac{\text{mass}\;\left(\text{exp}\right)-\text{mass}\;\left(\text{theo}\right)}{\text{mass}\;\left(\text{theo}\right)},\times ,10^{6}\right| \end{array}$$

The max-min normalization method was used to standardize the peak intensities of the product ions within a specified range of mass (e.g., *m/z* 200 to 400) in the positive-ion mode. Noncharacteristic product ions such as noise having a minimum relative intensity greater than 0.1 were excluded

$$I_{\text{norm},i}=\frac{I_{i}-I_{\min}}{I_{\max}-I_{\min}}$$

where $I_{i}$ is the intensity of *i*th product ion, $I_{\min}$ is the minimum intensity, $I_{\max}$ is the maximum intensity, and $I_{\text{norm},i}$ is the normalized intensity.

Product ion spectra having a number of matching ions greater than or equal to a specified threshold were considered to be valid matching spectra and added to the result list.

*De novo molecular formula and adduct ion determination.* The molecular formulae of the precursor ions and adduct ions were determined using a top-down approach. The element types (e.g., C, H, O, and N) and their numerical ranges were defined. A combinatorial algorithm was then used to generate all the formulae that were mathematically possible within the specified constraints, resulting in a comprehensive formula library. $\text{formula}\;\mathrm{DB}=C_{a}H_{b}O_{c}N_{d}$ , $\begin{array}{c} a\;\in \;[C_{\min},C_{\max}],b\in \left[H_{\min},H_{\max}\right],c\in \left[O_{\min},O_{\max}\right],d\in \left[N_{\min},N_{\max}\right] \end{array}$ . Specific adducts were considered based on ionization mode: Typical adducts were included in the positive-ion mode, such as [M + H]^+^, [M + NH_4_]^+^, [M + H − H_2_O]^+^, [M + CH_3_CN + H]^+^, and [M + CH_3_NH_2_ + H]^+^ whereas, in the negative mode, [M − H]^−^ and [M + HCOO]^−^. The resulting extended library could be systematically used to match the experimental precursor ion peaks, thereby ensuring comprehensive coverage of all possible molecular ions and adduct combinations. The theoretical mass was calculated from the composition formula to generate a range of theoretical mass within ±5 ppm. The measured mass in the query spectrum was matched with the theoretical mass range, and the composition formula having a hydrogen deficiency index range with the smallest error was determined to be the composition formula for the precursor ion.

*Neutral loss labeling of product ion pairs within a specific number of carbon atoms.* Labeling the precursor ions that exhibited specific characteristic neutral losses was essential for determining the module structures. Typically, neutral loss analysis involves calculating the mass differences between all ion pairs, including the precursor and product ions; this calculation was constrained by ion intensity and mass accuracy and led to low reliability (*36*). In our workflow, the neutral loss labeling of product ion pairs having a certain number of carbon atoms was proposed to precisely calculate the losses. The product ions in the specified mass range $\left[m/z_{\min},m/z_{\max}\right]$ were subjected to the max-min normalization method, and their formulae, $f_{i}$ , were calculated. A set of formulae $\left\{f_{i}\right\}$ from the above calculations was filtered to match the number of target carbon atoms, $C_{\text{target}}$ , $F_{C\;\text{target}}=\left\{f_{i}|C\left(f_{i}\right)=C_{\text{target}}\;\right\}$ . The number of carbon atoms in the formula pairs $\left(f_{i},f_{j}\right)$ , where $f_{i},f_{j}\in F_{C\;\text{target}}$ , calculated the differences in formula of their neutral losses. Last, labeling the precursor ions contained the target neutral losses whether Δf matched the target neutral losses. To prioritize informative neutral losses, a priority score was calculated by combining the ion pair intensity and balance. For the average intensity of the ion pair (IA), $\text{IA}=\frac{I_{1}+I_{2}}{2}$  ; and for the intensity difference factor (IDF), $\text{IDF}=\frac{1}{1+\frac{|I_{1}-I_{2}|}{\text{IA}}}$  ; neutral loss score=IA×IDF.

*Feature ion labeling of specific product ion sets.* The labeling of precursor ions containing the characteristic product ions was also required for module structure annotation. The product ions in the specified mass range $\left[m/z_{\min},m/z_{\max}\right]$ were subjected to the max-min normalization method, and their formulae, $f_{i}$ , were calculated. Among the formula sets $\left\{f_{i}\right\}$ , individual specified characteristic ions, such as the $\text{feature}=C_{a}H_{b}O_{c}$ , or multiple specified characteristic ions, such as $\text{features}=\left\{C_{a_{1}},H_{b_{1}},O_{c_{1}},,,C_{a_{2}},H_{b_{2}},O_{c_{2}},,,\;,\ldots ,\;\right\}$ , were filtered and ranked as $F_{\text{sorted}}$ according to the relative intensities of the characteristic ions. The precursor ions with the highest intensity were labeled; $F_{\text{Top}-N}=\left\{f_{i}\in F_{\text{sorted}}|i\leq N\;\right\}$ . The priority feature ion score was defined as the $\text{feature}\;\text{ion}\;\text{score}=\frac{I_{i}}{I_{\max}}$.

*Spectral similarity comparison within a certain mass range.* For modular structures without characteristic neutral losses or product ions, a similarity comparison of the partial product ion spectra was used to differentiate the module structures. After normalizing the spectra within a certain mass range, we compared the cosine score between the query spectra and the standard spectra within the specified mass range $\left[m/z_{\min},m/z_{\max}\right]$ to determine the substructure of modules. The formula for the calculation of similarity within the specified mass range was modified as follows: R was the index set of matched peak pairs within the specified mass range; $Q_{i}$ and $S_{i}$ denoted the intensities of the query spectrum Q and the standard spectrum S , respectively, after the *i*th matching peak was normalized in the specified range; and $\sum_{i\in R}\;Q_{i}S_{i}$ indicated the sum of the product of the intensities of the query spectra Q and the standard spectra S within the specified mass range

$$\begin{array}{c} {\text{cosine score}}_{\left[m/z_{\min},m/z_{\max}\right]}=\frac{\sum_{i\in R}\;Q_{i}S_{i}}{\sqrt{\sum_{i\in R}\;Q_{i}^{2}}\cdot \sqrt{\sum_{i\in R}\;S_{i}^{2}}} \end{array}$$

*Module assembly and candidate ranking.* On the basis of the diagnostic features extracted above and the elucidation of the MS/MS fragmentation of the target CNPs, the conditions for structural classification and module-level annotation were established. Users can define custom sets of diagnostic product ions or characteristic neutral losses and then specify the module selection and combination rules accordingly.

User-defined rules guided the generation of the Top-*N* candidate module sets, representing the most probable substructure combinations. Each candidate module set was then searched against the pseudo-CNP module-product ion library. Matching substructures were retrieved and assembled into complete pseudo-CNP structures, which were subsequently ranked according to the annotation criteria. The priority score of each candidate was calculated as follows

$$\text{Priority score}=\sum_{\text{ions}\;}\;w_{i}\cdot s_{i}+\sum_{\text{losses}\;}\;w_{j}\cdot s_{j}$$

where $s_{\text{ion}}^{\left(i\right)}$ is the score of the *i*th diagnostic product ion, $s_{\text{loss}}^{\left(j\right)}$ is the score of the *j*th characteristic neutral loss, and w is the binary indicator (0 or 1) denoting whether the ion/loss was required by the module annotation of the candidates.

The CNPs-MFSA application with a graphical user interface was coded in Python, and it included the following functions for the target experiments: target recognition, determination of molecular formula and adduct ion, neutral losses labeling, feature ion labeling, comparison of spectra, and user-defined rules to guide the generation of the Top-*N* candidates.

### Daphnane-type diterpenoids as proof-of-concept target CNPs

Daphnane-type diterpenoids were selected as target CNPs for a proof-of-concept study for the following reasons. Daphnanes have highly complex structures that comprise more than three rings, 8 to 11 contiguous chiral centers, and 7 to 14 functionalities (*37*). Our previous study has demonstrated that daphnanes, such as gnidimacrin, exhibited potent anti-HIV activities (*31*). Meanwhile, of the 215 reported daphnanes derived from Thymelaeaceae plants, only three daphnanes, namely, yuanhuacine (CCMSLIB00005725194), montanin (CCMSLIB00000846324), and pimelea factor P_4_ (CCMSLIB00005724873), had MS/MS data that could be accessed through a SMILES search in GNPS public spectral libraries, which meant that structural annotation by library matching was challenging.

#### LC-MS analysis of in-house daphnanes

The elucidation of modular fragmentation of daphnane-type diterpenoids was conducted using an in-house daphnane library containing 58 previously isolated compounds (figs. S4 and S5 and table S1). A Q Exactive quadrupole-Orbitrap mass spectrometer coupled with ultrahigh-performance LC was used for the analysis of the in-house daphnanes. MS and MS/MS data were acquired in both the positive-ion and negative-ion modes. Because the product ion spectra obtained in the positive-ion mode provided more information for structure elucidation, the modular analysis mainly used the positive-ion mode. In addition, high-resolution electrospray ionization (HR-ESI) MS was used with the higher-energy collision dissociation (HCD) method in an investigation of the fragmentation patterns, with a focus on identifying characteristic product ions and neutral losses that corresponded to specific structural modules within the daphnane-type diterpenoids.

#### Representative modular fragmentation elucidation

**Fig. 3.**
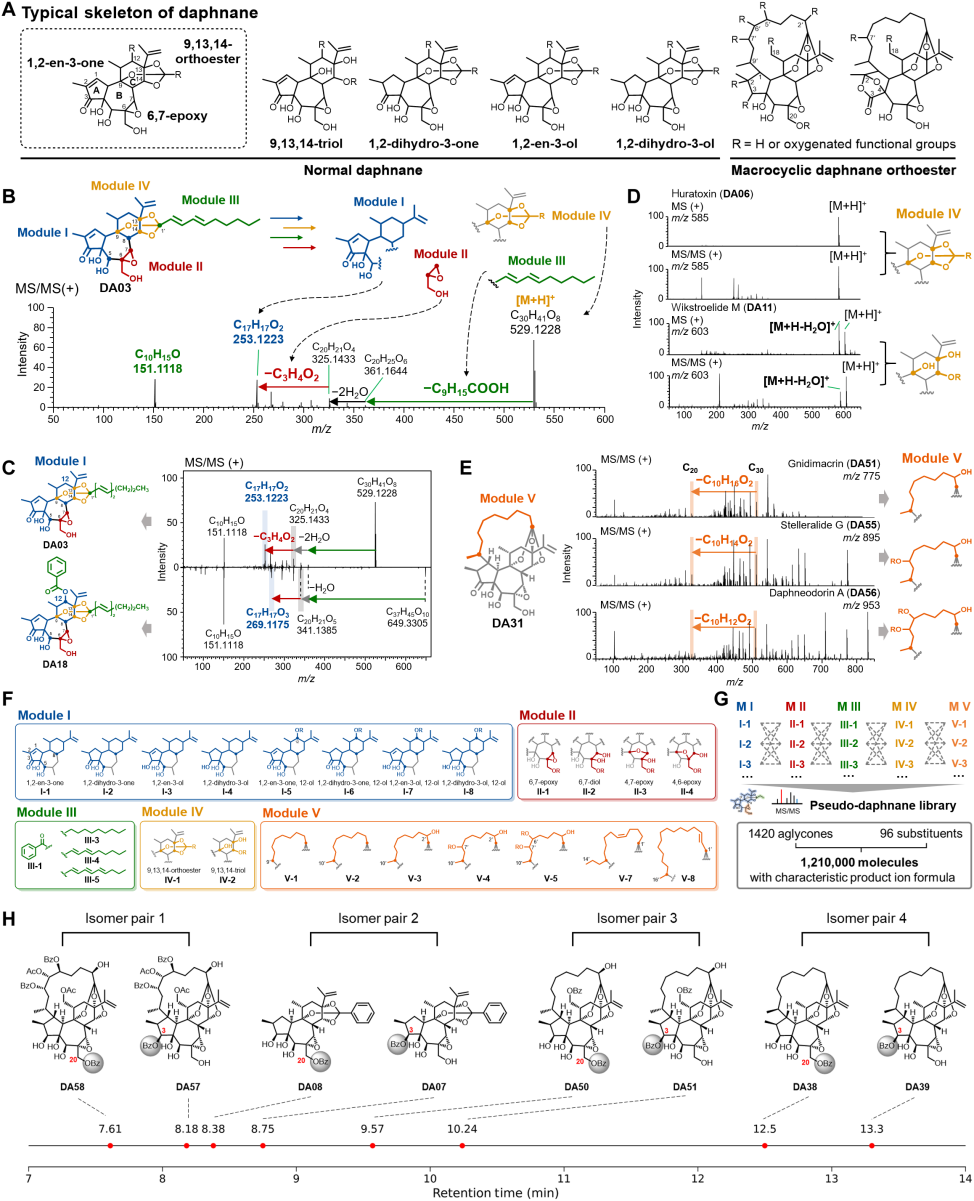
Modular fragmentation–based structural disassembly of daphnane-type diterpenoids. (**A**) Main structures of daphnane-type diterpenoids, containing normal daphnane and macrocyclic daphnane orthoester. (**B**) Modular fragmentation–based structural disassembly of typical daphnane-type diterpenoids to obtain modules I to IV, as exemplified for excoecariatoxin (**DA03**). (**C**) Example of the definition of different submodules of module I via comparison of product ion spectra between excoecariatoxin (**DA03**) (top) and yuanhuacine (**DA18**) (bottom). (**D**) Example of the definition of submodules of module IV via mass and product ion spectra. (**E**) Modular fragmentation–based structural disassembly of macrocyclic daphnane othroesters to give module V. (**F**) Representative modules I to V of daphnane-type diterpenoids. (**G**) The pseudo-daphnane library contained 1420 aglycones together with 96 acyl substituents constructed by modular assembly and product ion prediction. (**H**) Four pairs of structural isomers: **DA58** versus **DA57**, **DA08** versus **DA07**, **DA50** versus **DA51**, and **DA38** versus **DA39**, each consisting of C-20 and C-3 substituted daphnane diterpenoids.

Structurally, the 215 reported Thymelaeaceae daphnanes were mainly divided into normal daphnanes (which accounted for 70%) and macrocyclic daphnane orthoesters (which accounted for 30%), including the oxidative skeletons shown in Fig. 3A and figs. S4 and S5.

Among normal daphnanes, the typical skeleton with the 1,2-en-3-one structure in the A ring, the 6,7-epoxy moiety in the B ring, together with the 9,13,14-orthoester in the C ring is the most common oxidation pattern, accounting for 52% (fig. S4). The representative module disassembly of typical daphnanes was described in detail using excoecariatoxin (**DA03**), yuanhuacine (**DA18**), and simplexin (**DA04**) as model compounds. Typical daphnanes, such as excoecariatoxin (**DA03**), generated a product ion spectrum using a protonated molecule as the precursor ion (Fig. 3B). According to our previous investigation (*38*), the acyl substituents attached to the orthoester moieties on the C ring were cleaved in the product ion spectra of typical daphnanes. This cleavage resulted from simultaneous remote hydrogen rearrangement at C-9, C-13, and C-14. Subsequent continuous dehydration led to the loss of 2H_2_O and the seven-membered ring contraction resulted in the C_3_H_4_O_2_ loss derived from the 6,7-epoxide moiety on the B ring (fig. S5). Therefore, a characteristic product ion of *m/z* 253 (C_17_H_17_O_2_) was observed for the highest intensity, and the C_17_ product ions were defined as module I corresponding to the A─C rings of the daphnane skeleton. The characteristic neutral loss of C_3_H_4_O_2_ was defined as module II, which was associated with the substructure of the B ring. In addition, the neutral losses between the precursor ions and the initial C_20_ product ions, such as *m/z* 361 (C_20_H_25_O_6_) in excoecariatoxin (**DA03**), as well as the product ions that appeared in the low-mass range, were defined as module III, corresponding to the substructure associated with the acyl substituents. Furthermore, yuanhuacine (**DA18**) was structurally similar to excoecariatoxin (**DA03**), with the main difference being that a benzoyloxy group was attached to C-12, and they shared a common fragmentation pattern (Fig. 3C). Given their structural variations, the product ion defined as module I in yuanhuacine (**DA18**) was observed at *m/z* 269 (C_17_H_17_O_3_), which represented another submodule type of module I. To further verify that the C_17_ product ion variation defines all the substructures of module I related to the A─C rings, a reduction reaction was conducted on typical daphnane. Simplexin (**DA04**) was used as a standard to reduce the 1,2-en-3-one structure in ring A and the isopropenyl group in ring C, which yielded the derivatives **DA04a**, **DA04b**, and **DA04c** (fig. S9). The product ions **DA04a** to **DA04c** underwent mass shifts corresponding to the amount of hydrogen added during the reduction relative to **DA04**, suggesting that the structure of the A ring and the isopropenyl group had no influence on the value of the characteristic neutral losses, and the specific elimination of C_3_H_4_O_2_ occurred in the B ring. The reaction further confirmed that the structural modifications altered the formula of the C_17_ product ion, which was defined as module I (fig. S10). Differences in the oxidative modifications of the C ring were defined by the fragment ions observed in the mass spectra. Although 9,13,14-orthoester moieties were formed on the C ring, the protonated molecules [M + H]^+^ were detected with the highest intensity in the mass spectrum. However, as was exemplified by daphnanes, such as wikstroelide M (**DA11**), which featured a 9,13,14-triol structure, the mass spectrum showed that the dehydration fragment ion [M + H − H_2_O]^+^ exhibited higher intensity than the protonated molecule [M + H]^+^. It was suggested that, because of the cleavage of the orthoester moiety, the presence of a hydroxy group at the tertiary carbon (C-13) was unstable and, before any fragmentation, underwent dehydration in the mass spectrum. The protonated molecules [M + H]^+^ and the dehydration fragment ion [M + H − H_2_O]^+^ were observed both in the mass and product ion spectra of daphnanes having the 9,13,14-triol structure in the C ring, which were used to define module IV (Fig. 3D).

Macrocyclic daphnane orthoesters, another type of daphnanes, are characteristic of a C_10_─C_16_ aliphatic chain attached with zero to four oxidative substituents spanning the daphnane skeleton through the 9,13,14-orthoester moiety and linking to C-1 of the A ring (*37*). Representative disassembly modules of this type were described using gnidimacrin (**DA51**), stelleralide G (**DA55**), and daphneodorin A (**DA56**) as model compounds (Fig. 3E). Macrocyclic daphnane orthoesters with varied oxidative moieties were all characterized by the abundance of C_30_ to C_27_ product ions with high intensity that were observed in the range of *m/z* 400 to 550 (*38*). Furthermore, the continuous dehydration and retro-ene reaction led to the generation of C_20_ product ions derived from the daphnane skeleton. In the product ion spectra, the neutral losses of C_10_H_16_O_2_, C_10_H_14_O_2_, and C_10_H_12_O_2_ were associated with the substructures of the macrocyclic rings with one, two, and three substituents, which were assigned to gnidimacrin (**DA51**), stelleralide G (**DA55**), and daphneodorin A (**DA56**), respectively. The above specific neutral losses correspond to the macrocyclic moieties, which allows us to define the structure of module V (Fig. 3F). This module was associated with the macrocyclic structures of the macrocyclic daphnane orthoesters.

Four pairs of structural isomers (**DA58** versus **DA57**, **DA08** versus **DA07**, **DA50** versus **DA51**, and **DA38** versus **DA39**), each consisting of C-20 and C-3 substituted daphnane diterpenoids, respectively, were used as an example to demonstrate the isomer differentiation based on retention behavior. For macrocyclic daphnane orthoesters, an increase in the number of polar substituents leads to higher overall polarity and consequently shorter retention times (RTs). In all four cases, the C-20 substituted daphnanes consistently exhibited shorter RTs than their C-3 substituted counterparts (*Rt*_C-20_ < *Rt*_C-3_), as observed in the total ion chromatograms. The retention pattern provides a practical basis for distinguishing positional isomers during annotation for other isomeric compounds with similar core structures. The specific substitution pattern can be inferred by comparing their retention behavior with that of known standards. Such retention-based inference, combined with diagnostic MS/MS features, enables reliable structural assignment of structural isomers (Fig. 3H).

#### Construction of the pseudo-daphnane library

On the basis of the module definition established through the above fragmentation elucidation and the structure variations summarized in the literature, a pseudo-daphnane module-product ion was constructed using SQLite (*39*) that contained 1420 aglycones and 96 acyl substituents (Fig. 3G). After the further combination of aglycone and substituents, 1,210,000 molecules and corresponding characteristic product ion molecular formulae were generated, which covered all possible substructure combinations of Thymelaeaceae family. The generated product ion molecular formulae containing in the pseudo-daphnane library was used as the filtering condition for recognizing target daphnanes in complex mixtures and performing module annotation.

### Method evaluation of CNPs-MFSA

#### Targeted annotation beyond molecular network clustering

**Fig. 4.**
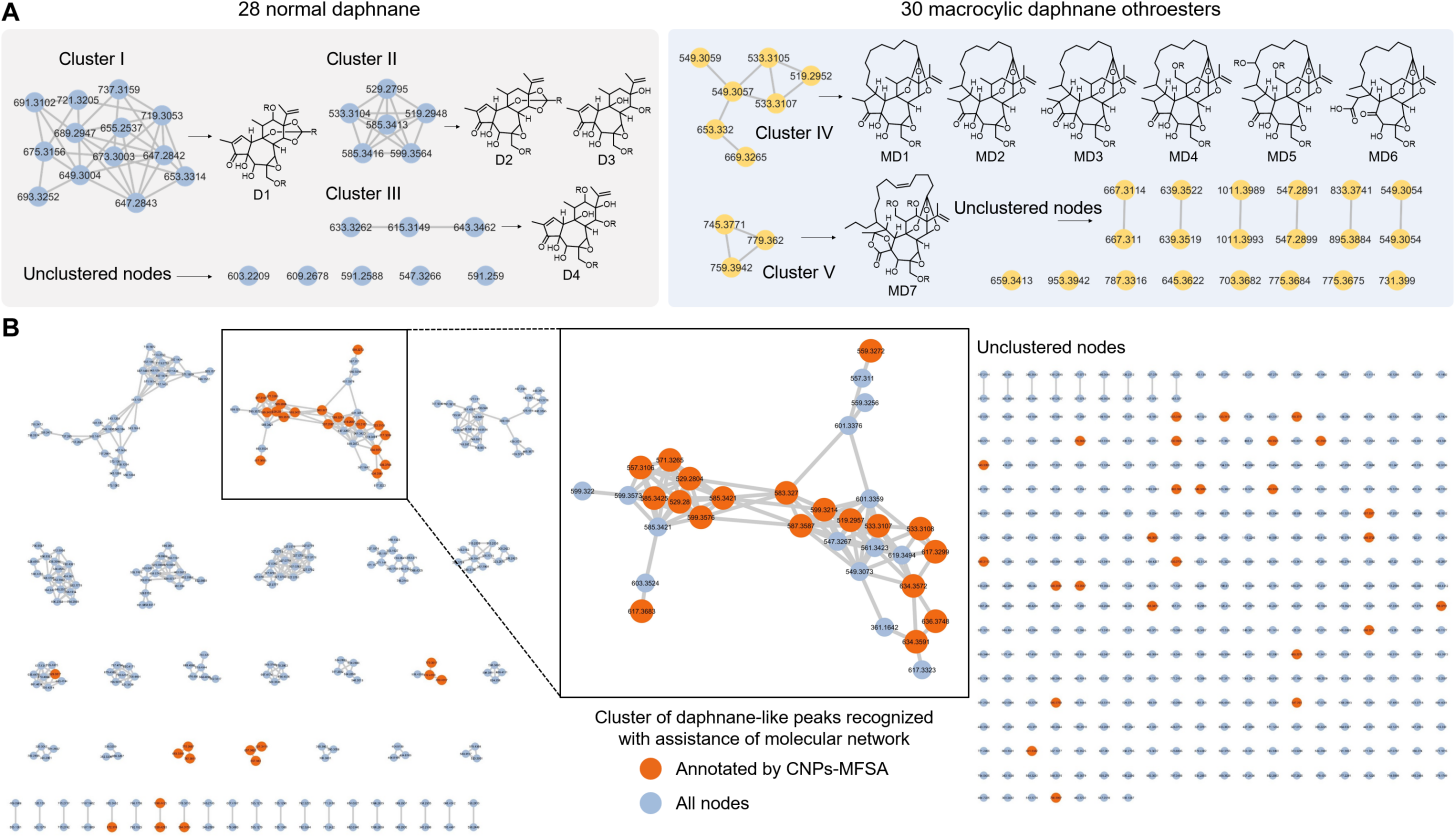
Targeted annotation beyond molecular network clustering. (**A**) Molecular network analysis of 58 in-house daphnanes. (**B**) Molecular network analysis of the methanol extract from the stem of *W. indica*, with orange nodes recognized by CNPs-MFSA.

Classical molecular network analysis was conducted using an in-house daphnane library by MetGem (*40*) (Fig. 4A). The cluster threshold was set using a cosine score of 0.70 or higher to group the nodes by spectral similarity. The molecular network of 58 in-house daphnanes contained five clusters (I to V), among which half of the daphnanes (28/58) were nonclustered due to low spectral similarity and high structural complexity. As for the normal daphnanes, the structural similarity was inconsistent with the spectral similarity, such as clusters I to III corresponding to structures D1 to D4, thereby preventing the clustering of nodes within the network. Although, in macrocyclic daphnane orthoesters, six structure types, MD1 to MD6, were clustered in one subnetwork IV, they generated high similarity spectra and accurate substructure differentiation of these compounds was challenging. In addition, a spectra library search after the molecular network analysis revealed that one node, yuanhuacine, was annotated. The above problem raised the risk of missing many nodes of target compounds during molecular networking analysis on plant extracts. As exemplified by *Wikstroemia indica*, 34 daphnane-like peaks were clustered and recognized with the assistance of a molecular network. Using CNPs-MFSA, we annotated 67 daphnanes from 617 valid peaks, including the extra annotation of 30 daphnanes from the unclustered nodes (Fig. 4B).

#### Evaluation CNPs-MFSA against SIRIUS, MS-FINDER, and MetFrag

**Fig. 5.**
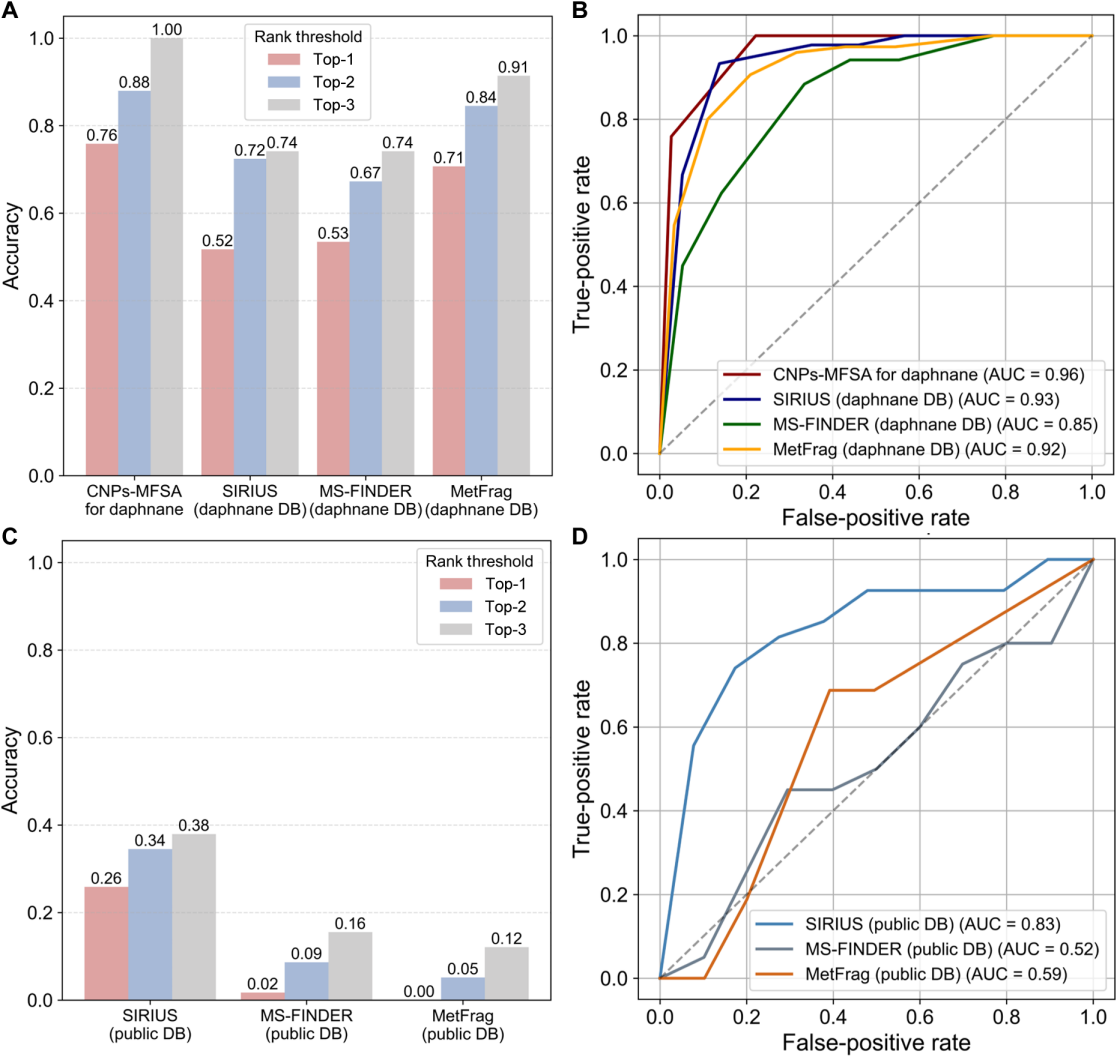
Comparison of CNPs-MFSA with SIRIUS, MS-FINDER, and MetFrag. (**A**) Ranking accuracy (Top-1 to Top-3) and (**B**) Receiver operating characteristic (ROC) curves analysis of 58 in-house daphnane dataset (including normal daphnanes and macrocyclic daphnane orthoesters) both using a custom known Thymelaeaceae daphnanes library as candidate pool. (**C**) Ranking accuracy (Top-1 to Top-3) and (**D**) ROC curves analysis of 58 in-house daphnane dataset (including normal daphnanes and macrocyclic daphnane orthoesters) both using a default public NP database.

The evaluation of CNPs-MFSA was done on the in-house daphnane library (including 58 compounds), which was processed by MZmine to generate a standard dataset. The annotation accuracy was evaluated using three commonly used annotation tools, SIRIUS, MS-FINDER, and MetFrag, under two distinct conditions: (i) using the reported 215-daphnane library (Fig. 5, A and B) and (ii) using default public compound databases (Fig. 5, C and D). Under the daphnane-specific library setting (Fig. 5, A and B), CNPs-MFSA achieved the best accuracy (76%) and overall area under the curve (AUC) (0.96) when compared with SIRIUS (Top-1: 52%, AUC: 0.93), MS-FINDER (Top-1: 67%, AUC: 0.85), and MetFrag (Top-1: 71%, AUC: 0.92). This result confirms that, even with equivalent candidate pools, CNPs-MFSA delivers substantially better prioritization of correct structures, particularly at the Top-1 and Top-2 thresholds. CNPs-MFSA consistently outperformed alternatives across all the Top-*k* thresholds. When it was evaluated on the public databases (Fig. 5, C and D), its performance across all methods declined sharply due to the larger and more structurally diverse search spaces. Nevertheless, CNPs-MFSA remained robust in its targeted setting. In contrast, SIRIUS, MS-FINDER, and MetFrag exhibited substantial drops in the Top-3 accuracy (38%, 16%, and 12%, respectively) and overall AUC, highlighting the challenge of accurately identifying CNPs on large-scale public libraries. The above results demonstrated that CNPs-MFSA offers a distinct advantage for the targeted annotation of CNPs, especially when it is applied in a constrained, chemically relevant candidate space.

#### Statistical analysis

A sensitivity analysis was simulated incomplete or suboptimal 1420 module sets. Specifically, we randomly reduced the coverage of the original module set to 50, 33, and 10%, generating 10 pseudo-daphnane libraries for each coverage level to mimic varying degrees of design incompleteness (fig. S34). The analysis revealed that, when the module set was reduced to one-third of the original size, a noticeable drop in identification accuracy of reference compounds was observed. This degradation became more pronounced when the module set was reduced to one-tenth, indicating that incomplete or poorly constructed module sets can noticeably impair annotation performance. These findings underscore the critical role of rational and comprehensive module design in ensuring the robustness and accuracy of the CNPs-MFSA workflow.

Tiglianes feature a 5/7/6/3-tetracyclic carbon skeleton that is structurally similar to daphnane-type diterpenoids and exhibits comparable product ion spectra. A mixed dataset of 58 in-house daphnanes and 16 tigliane-type diterpenoids was used for statistical significance analysis to evaluate the ability of CNPs-MFSA to distinguish structurally similar compounds (fig. S33). During the initial step of structural annotation, targeted recognition, CNPs-MFSA incorporates around 9% of tiglianes into the candidate pool for daphnanes due to their spectral similarity. Nevertheless, these candidates were entirely excluded in the downstream structural annotation workflow, ensuring high specificity. This evaluation is visualized in fig. S35, where the performance of the target recognition module and the downstream module annotation process was assessed using precision, accuracy, recall, and F1-score metrics. Although spectral overlap between daphnanes and tiglianes led to minor inclusion of tiglianes during the initial candidate retrieval stage (true-positive rate: 78.4%; false-positive rate: 9.5%), none of these structurally similar but incorrect candidates passed the full scoring and filtering criteria in the final annotation step. This is reflected by a false-positive rate of 0.0% in the module annotation output.

### Application of CNPs-MFSA to annotation daphnanes in Thymelaeaceae extracts

**Fig. 6.**
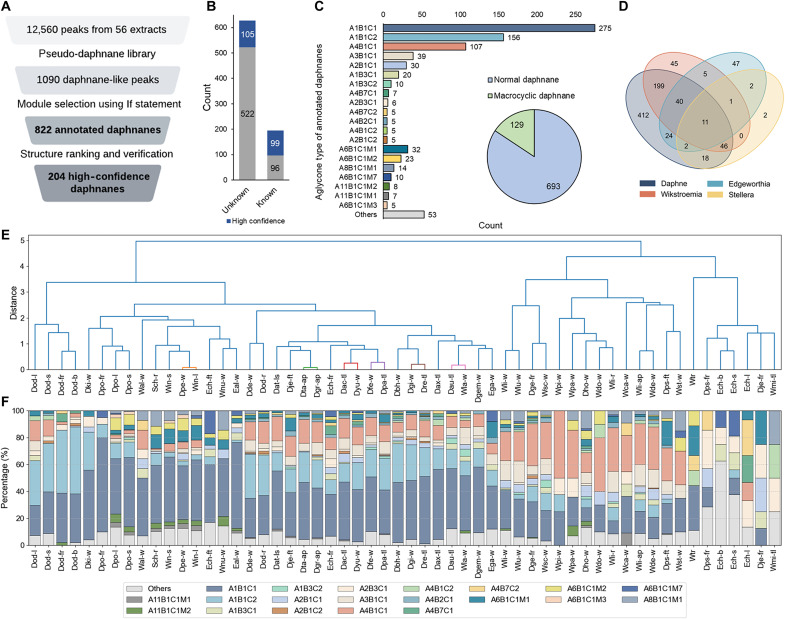
Comprehensive annotation of daphnanes in Thymelaeaceae extracts using CNPs-MFSA. (**A**) Procedure of daphnane identification in 56 Thymelaeaceae extracts. (**B**) Count of known and undescribed daphnanes with high-confidence results. (**C**) Aglycone-type classification of 822 annotated daphnanes. (**D**) Distribution of annotated daphnanes across four genera. (**E**) Hierarchical clustering of daphnane profiles across 56 extracts. (**F**) Proportional distribution of annotated daphnane skeleton types in 56 extracts.

The Thymelaeaceae family comprises over 800 species across 53 genera. The 215 reported Thymelaeaceae daphnanes were distributed across 54 species within 16 genera, among which the genera *Daphne*, *Wikstroemia*, and *Stellera* contained the greatest number of reported daphnanes (fig. S16). Therefore, 56 extracts from Thymelaeaceae plants, including *Daphne* (30 extracts), *Wikstroemia* (18 extracts), *Stellera* (1 extract), and *Edgeworthia* genera (6 extracts), were used to evaluate the applicability of CNPs-MFSA (table S3). After the LC-MS data were processed with MZmine, 12,560 dereplicated peaks were extracted from the 56 datasets. A total of 1090 daphnane-like peaks were filtering using the pseudo-daphnane library constructed as aforementioned. Furthermore, 822 daphnanes were identified by module selection and annotation, among which 204 daphnanes were annotated with high-confidence results, including 105 compounds that were putatively identified as previously undescribed candidates (Fig. 6, A and B). Structurally, the 822 annotated daphnane were divided into 693 normal daphnane (D) and 129 macrocylic daphnane orthoesters (MD), together with 21 subclasses with different oxidation skeleton, among which skeleton A1B1C1 with 1,2-en-3-one in the A ring, 6,7-epoxy in the B ring, as well as 9,13,14-orthoester in the C ring was the most common pattern counted for 33%, suggesting a prevalence of specific structural subclasses within the Thymelaeaceae extracts (Fig. 6C and fig. S18). Intergeneric differences among four genera were shown in Fig. 6D, 412 specific compounds from *Daphne*, 45 specific compounds from *Wikstroemia*, 47 specific compounds from the *Edgeworthia* genus, reinforced *Daphne* as a chemically prolific genus within Thymelaeaceae. Hierarchical clustering based on structures types and proportion of annotated daphnane revealed distinct chemotypic variations between the 56 extracts (Fig. 6E). Plant samples that were closely clustered (distance < 0.5), as exemplified by *Daphne pedunculata* (Dpe-w) and *W. indica* (Win-l), *D. tangutica* (Dta-ap), and *D. grueningiana* (Dgr-ap), indicated high metabolic similarity. Clusters aligned strongly with genera, particularly in *Daphne* and *Wikstroemia*, indicating genus-specific metabolite profiles. A proportional analysis of daphnane subclasses showed that A1B1C1, A1B1C2, and A4B1C1 were dominant across most samples, especially in *Daphne* and *Wikstroemia* (Fig. 6F). Less abundant subclasses (e.g., A6B1C1M7) were restricted to the specific genus *Edgeworthia*, underscoring potential taxonomic markers for these genera.

**Fig. 7.**
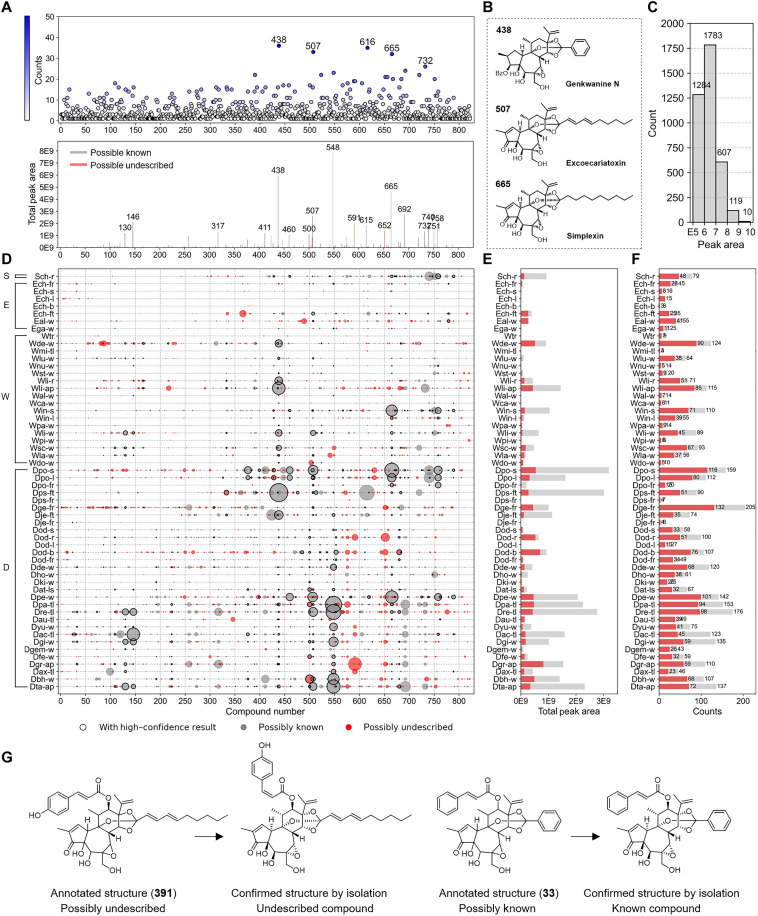
Comparative distribution of daphnanes in Thymelaeaceae extracts. (**A**) Frequency of the occurrence of 822 annotated daphnanes (top), and total peak area of 822 annotated daphnanes including known and undescribed compounds (bottom). (**B**) Structures of top 3 common and high-content daphnanes in the 56 Thymelaeaceae extracts. (**C**) Peak area distribution of annotated daphnane compounds. (**D**) Distribution of known and undescribed daphnanes across 56 plant samples with high-confidence results. (**E**) Total peak area of annotated daphnanes across 56 plant samples including known and undescribed compounds. (**F**) Frequency and distribution of annotated daphnanes across 56 plant samples including known and undescribed compounds. (**G**) Annotated structures of undescribed compounds **391** and known compound **33**, together with NMR-based elucidated structures.

A comprehensive distribution of annotated daphnanes, including previously known and undescribed, was shown in Fig. 7. Genkwanine N (**438**), excoecariatoxin (**507**), and simplexin (**665**) were the Top-3 common daphnanes in the 56 Thymelaeaceae extracts [Fig. 7, A (top) and B]. Meanwhile, yuanhuajine (**548**), genkwanine N (**438**), and simplexin (**665**) were the Top-3 high content in the 56 extracts [Fig. 7, A (bottom), and B]. The aerial part of *D. grueningiana* (Dgr-ap) highlighted the most intense content of daphnanes and the most possibly plant sample to obtain undescribed daphnanes for further phytochemical investigation, followed by the bud and root of *D. odora* (Dod-b and Dod-r) (Fig. 7E). In total, 3803 daphnane-derived peaks (contain replicated peaks) were annotated from 56 Thymelaeaceae plant extracts, among which 3067 daphnane-derived peaks were trace amount components within the peak area range of 1 × 10^5^ to 1 × 10^7^, and 129 daphnanes were over peak area of 1 × 10^8^, indicating the high annotation capability from low to high content (Fig. 7C). The detailed daphnanes distribution across 56 Thymelaeaceae plant extracts was shown in Fig. 7 (D to F), with bubble sizes corresponding to relative peak areas. The flowers of *D. genkwa* were the plant samples containing the highest amount of daphnanes. On the basis of the annotation results, we selected two plant extracts, *D. genkwa* (flower) and *D. odora* (bud), which exhibited a relatively high number and abundance of possibly undescribed compounds, for targeted isolation and NMR-based structural elucidation (figs. S29 to S32). Possibly undescribed compound **391** was annotated as normal daphnane with the 1,2-en-3-one in the A-ring, 6,7-epoxy in the B-ring, and 9,13,14-orthoester in the C-ring, together with coumaroyloxy and decadienoyl moieties, which was further verified in the phytochemical investigation of *Daphne odora* (bud). The previously undescribed structure of **391** was revised as 12-(*Z*)-coumaroyloxy-6,7-epoxy-5-hydroxyresiniferonol-9,13,14-ortho-(2*E*,4*E*)-decadienoate (Fig. 7G). Possibly known compound **33**, which was isolated from *D. genkwa* (flower), was further validated as gnidicin. Although there are still challenges in distinguishing the geometric isomers, both **391** and **33** were consistent with the annotated planar structure.

Publicly available MS/MS data downloaded from GNPS were also used as a test dataset and processed through the CNPs-MFSA workflow. A total of 6192 features were analyzed, resulting in the targeted annotation of 77 daphnane-type diterpenoids. Among these, 24 compounds were identified as high-confidence matches, including 8 previously unreported structures. These results demonstrate the applicability and scalability of the method on public datasets, further highlighting its utility in discovering both known and previously undescribed daphnane-type compounds from CNP mixtures (table S14).

### Extension of the MFSA workflow for targeted annotation of other CNPs

To demonstrate the generalizability and modularity of the MFSA workflow beyond daphnane-type compounds, our strategy was applied to three additional structurally distinct classes of CNPs: aconitine analogs (aconitine-type alkaloids), paclitaxel analogs (taxane-type diterpenoids), and obakunone analogs (A,D-*seco* limonoid-type triterpenoids) (figs. S19 to S28). The above three classes were selected based on their structural complexity, well-documented bioactivities, and chemical diversity, including nitrogen-containing alkaloids and low-abundance components in medicinal plant materials. For aconitine analogs, C_19_-diterpenoid alkaloids (aconitine, jesaaconitine, mesaconitine, and hypaconitine) were used to construct a pseudo-library of 144 compounds (*41*–*43*). Diagnostic fragmentation features were defined using MS/MS data of 10 standards from the GNPS database. Application of MFSA to Aconiti Radix Processa extract led to the recognition of 68 aconitine-liked peaks from 748 peaks, demonstrating that the workflow accommodates nitrogen-containing scaffolds. For paclitaxel analogs, a pseudo-library of 620 compounds was generated from 34 known taxane-type structures (*44*–*46*). Fragmentation modules were built from seven standards downloaded from GNPS. The MFSA strategy enabled the recognition of 39 paclitaxel-liked peaks from the *Taxus brevifolia* dataset containing 2561 peaks (table S6). For obakunone analogs, A,D-*seco* limonoid scaffolds were used to generate 141 pseudo-compounds (*47*). Fragmentation templates were derived from three in-house standards and 34 literature-curated structures. Despite their low abundance in medicinal plant extracts, MFSA successfully annotated 23 obakunone analogs from extracts from Citri Unshiu Pericarpium, Phellodendri Cortex, and Aurantii Fructus Immaturus (table S6). These findings collectively underscore the broad adaptability of MFSA across structurally diverse and chemically challenging CNP classes, including alkaloids, diterpenoids, and triterpenoids, thereby supporting its role as a scalable and structure-guided strategy for targeted annotation in CNP matrices.

## DISCUSSION

The MFSA strategy proposed in this study provided a transformative approach for the automated annotation of CNPs. By disassembly of CNPs into modular units based on fragmentation patterns, generating a pseudo-CNP library, and systematically reassembling structures, the MFSA strategy overcomes fundamental limitations inherent to conventional annotation approaches. Unlike conventional techniques such as NMR or x-ray crystallography (*8*), which are time-consuming and require large quantities of pure compounds, CNPs-MFSA enabled high-throughput, accurate structural annotation using minimal sample amounts, as evidenced by its application to daphnane-type diterpenoids, further extended to other classes of structurally CNPs, including aconitine analogs (aconitine-type alkaloids), paclitaxel analogs (taxane-type diterpenoids), and obakunone analogs (A,D-*seco* limonoid-type triterpenoids).

However, CNPs-MFSA exhibits aspects that could benefit from further exploration. For instance, low-intensity signals can produce suboptimal MS/MS spectra, increasing the risk of false-positive annotations. In the current workflow, isomer differentiation is partially achieved through the integration of RT information derived from standard datasets representing major daphnane subclasses. The RT-based inference system is currently implemented manually and depends on empirical matching with locally curated datasets, limiting both its reproducibility and scalability. Moreover, the MFSA strategy currently relies primarily on heuristic methods for identifying module boundaries and mapping diagnostic ions, but emerging machine learning–based prediction models offer promising avenues for improving this strategy. ICEBERG leverages neural networks to simulate molecular fragmentation graphs and provides a more accurate model for compound identification (*48*). In future iterations of MFSA, we anticipate integrating predictive algorithm–based fragmentation models trained on large datasets, aiming to enhance predictive accuracy, facilitate isomer differentiation, and broaden the applicability of automated annotation across diverse classes of structurally CNPs.

Nevertheless, CNPs-MFSA could speed up the discovery and structural elucidation of NPs and offer a scalable and adaptable framework for target experiments of other CNPs. Moreover, the open-source and user-friendly GUI provides researchers with a flexible platform for further customization and optimization.

## MATERIALS AND METHODS

### Materials

All 58 daphnane standards (**DA01** to **DA58**; figs. S4 and S5) were isolated in our previous phytochemical investigation. Fifty-six plant species of the Thymelaeaceae family were collected from People’s Republic of China, Japan, and Turkey (table S3). LC-MS–grade methanol, distilled water, distilled water with 0.1% formic acid and acetonitrile with 0.1% formic acid were purchased from Kanto Chemical Co. Inc. (Japan). Sep-Pak C_18_ Plus Long Cartridge used in sample preparation of plant materials was purchased from Waters Associates (Milford, MA). Sodium borohydride (NaBH_4_) and palladium 10% on carbon (Pd/C, wetted with ~55% water) were purchased from Tokyo Chemical Industry Co. Ltd. (Japan). Amberlite IR-120 (H^+^) was purchased from Merck KGaA (Darmstadt, Germany).

### Preparation of analogues DA04a to DA04c of simplexin (DA04)

(i) Preparation of **DA04a**: A solution of NaBH_4_ (1.0 mg) in methanol (500 μl) was added to a solution of **DA04** (2.0 mg) in methanol (500 μl), and the reaction mixture was stirred at room temperature for 30 min. Strongly acidic cation exchange resin IR-120 (H^+^) (1.0 g) was added in the reaction solution to neutralize and stop the reaction. The neutralized reaction solution was extracted with ethyl acetate and water (1:1, *v/v*). The ethyl acetate fraction was purified by reversed-phase high-performance liquid chromatography (RP-HPLC) with CH_3_CN:H_2_O (8:2, *v/v*) to give **DA04a** (1.2 mg). (ii) Preparation of **DA04b**: Pd/C (10 mg) was added into a solution of **DA04** (2.0 mg) in methanol (1.0 ml), and then hydrogen was introduced into the sealed reaction vessel and stirred for 2 hours. The reaction solution was filtered through a syringe filter (0.45 μm) and purified by RP-HPLC with CH_3_CN:H_2_O (8:2, *v/v*) to give **DA04b** (1.4 mg). (iii) Preparation of **DA04c**: Pd/C (10 mg) was added into a solution of **DA04** (3.0 mg) in methanol (1.0 ml), and then hydrogen was introduced into the sealed reaction vessel and stirred for 2 hours. The reaction solution was filtered through a syringe filter (0.45 μm). After removal of solvent in vacuo, the residue was treated in the same procedure as (i) to give **DA04c** (1.0 mg).

### Sample preparation for standards and plant extracts

All 58 daphnane standards were dissolved in LC-MS–grade methanol at a concentration of 1 ppm. The air-dried plant materials of the Thymelaeaceae family were cut into small pieces and were extracted with 95% EtOH at room temperature to give the ethanol extracts and a residue. The ethanol extracts (100 mg per sample) were suspended in distilled water and then partitioned with ethyl acetate. The ethyl acetate fractions (5 mg per sample) were subjected to Sep-Pak C_18_ Plus Long Cartridge, eluted with LC-MS–grade methanol:water [5:5 and 10:0 (*v*/*v*), 10 ml for each solution] to afford two eluted solutions for each sample. The 100% methanol eluted solution for each sample was subjected to LC-MS analysis. Each sample solution was filtered through a syringe filter (0.22 μm) before LC-MS analysis.

LC-MS analysis for standards and plant extracts. LC was conducted using a Vanquish UHPLC system (Thermo Fisher Scientific, Waltham, MA, USA). Chromatographic peaks were separated on a YMC-Triart C_18_ column (150 mm by 2.1 mm, 1.9 μm) at a flow rate of 0.4 ml/min at 40°C in a column temperature oven. The mobile phase consisted of eluent A (distilled water with 0.1% formic acid) and B (acetonitrile with 0.1% formic acid) programmed as follows: 0 to 15 min, linear gradient 50 to 100% B, 15 to 18 min, 100% B, and then the column was reequilibrated at 50% B for 10 min before the next injection. The injection volume was 2.0 μl for analysis.

A Q Exactive hybrid quadrupole-Orbitrap high-resolution accurate mass spectrometer system (Thermo Fisher Scientific, Waltham, MA, USA) with an ESI source was operated in the positive-ion and negative-ion modes. The calibration solutions were used to calibrate the ESI-MS to increase mass accuracy. The optimized parameters of MS are listed as follows: spray voltage, +3.5 kV (for positive-ion mode) or −2.5 kV (for negative-ion mode); capillary temperature, 262.5°C; sheath gas flow rate, 50 U; auxiliary (AUX) gas flow rate, 12.5 U; sweep gas flow rate, 2.5 U; S-lens radio frequency level, 50 U; and probe heater temperature, 425°C. Data were collected in the full MS and full MS/data-dependent (dd) MS/MS modes. The in-source CID (collision-induced dissociation) was set at 0 eV, and the resolution was 70,000 for full MS and 35,000 for full MS/dd-MS/MS.

For standards, the scan range was set at *m/z* 150 to 2000 for full MS, whereas data-dependent scanning was performed using HCD with the normalized collision energy (NCE) for positive-ion and negative-ion modes at 15 eV. The automatic gain control (AGC) was set at 1.0 × 10^6^ for full MS and 2.0 × 10^5^ for dd-MS/MS.

For plant extracts, the scan range was set at *m/z* 300 to 1500 for full MS, whereas data-dependent scanning was performed using HCD with the stepped NCE for positive-ion mode at 15 and 20 eV and negative-ion mode at 10 eV. The AGC was set at 1.0 × 10^6^ for full MS and 1.0 × 10^5^ for dd-MS/MS. The maximum ion time (IT) was set for standards and plant extracts at 200 or 100 ms for full MS and 50 ms for dd-MS/MS.

### Data processing

The LC-MS raw data of standards and plant extracts were confirmed by FreeStyle 1.6. The raw data were further processed by MZmine 2.53 (*34*) to extract the features, and the detailed information was summarized as follows:

The mass detections were performed for standards with the noise level set at 1.0 × 10^5^ for MS1 and 5.0 × 10^2^ for MS2. Chromatogram building was carried out using the ADAP chromatogram builder module (*48*) for positive-ion mode with a minimum group size of 5, group intensity threshold of 1.0 × 10^5^, minimum height intensity of 1.0 × 10^5^, and *m/z* tolerance of 5 ppm. The built chromatograms were deconvoluted with a baseline cutoff algorithm using the following settings: minimum peak height of 2.0 × 10^6^, peak duration range of 0.00 to 5.00 min, and baseline level of 1.0 × 10^6^. Chromatograms were deisotoped using an isotopic peak grouper algorithm, with an *m/z* tolerance of 5 ppm and RT tolerance of 0.15 min. To align features, the join aligner was used with an *m/z* tolerance of 5 ppm, weight for *m/z* of 75, RT tolerance of 0.1 min, and weight for RT of 25. The precursor ions of 58 standards (table S1) were marked and selected using a feature list row filter with text in comment and keeping rows that match all criteria. Last, the features table of standards was exported as a quant table (.csv) and an MS^2^ spectral summary file (.mgf).

As for plant extracts (100% MeOH eluted solution for each plant material), the mass detections were performed with the noise level set at 1.0 × 10^4^ for MS^1^ and 1.0 × 10^3^ for MS^2^. Chromatogram building was carried out using the ADAP chromatogram builder module (*49*) with a minimum group size of 5, group intensity threshold of 1.0 × 10^3^, minimum height intensity of 1.0 × 10^4^, and *m/z* tolerance of 5 ppm for positive-ion mode and negative-ion mode, respectively. The built chromatograms were deconvoluted with a baseline cutoff algorithm using the following settings: minimum peak height of 1.0 × 10^5^, peak duration range of 0.01 to 3.00 min, and baseline level of 5.0 × 10^4^. Chromatograms were deisotoped using an isotopic peak grouper algorithm, with an *m/z* tolerance of 5 ppm and RT tolerance of 0.15 min. The feature list row filter was used to keep only peaks with MS^2^ scan and reset the peak number ID. Last, the features table was exported as .csv and .mgf files. The .csv and .mgf files obtained in positive-ion mode were input in the program to recognize diterpenoids-liked peaks and further automatic annotation.

## Supplementary Materials

### The PDF file includes:

Supplementary Text Figs. S1 to S35 Tables S1 to S9 Legends for tables S10 to S14 LC-MS dataset of standards and extracts
