# Supplementary material for: Automated annotation of complex natural products using a modular fragmentation–based structure assembly (MFSA) strategy
Source: Sci Adv. 2025 Aug 15;11(33):eadw4693. doi: 10.1126/sciadv.adw4693 (PMC12356265; doi:10.1126/sciadv.adw4693)
Supplement: Supplementary file 1 — Supplementary Text Figs. S1 to S35 Tables S1 to S9 Legends for tables S10 to S14 LC-MS dataset of standards and extracts [file sciadv.adw4693_sm.pdf]

Science Advances
AAAS

Supplementary Materials for

Automated annotation of complex natural products using a modular fragmentation–based structure assembly (MFSA) strategy

Mi Zhang *et al.*

Corresponding author: Kouharu Otsuki, kouharu.otsuki@phar.toho-u.ac.jp; Wei Li, liwei@phar.toho-u.ac.jp

Sci. Adv. **11**, eadw4693 (2025)
DOI: 10.1126/sciadv.adw4693

The PDF file includes:

Supplementary Text
Figs. S1 to S35
Tables S1 to S9
Legends for tables S10 to S14
LC-MS dataset of standards and extracts

Other Supplementary Material for this manuscript includes the following:

Tables S10 to S14

Supplementary Text

Pseudo-CNPs Library Construction

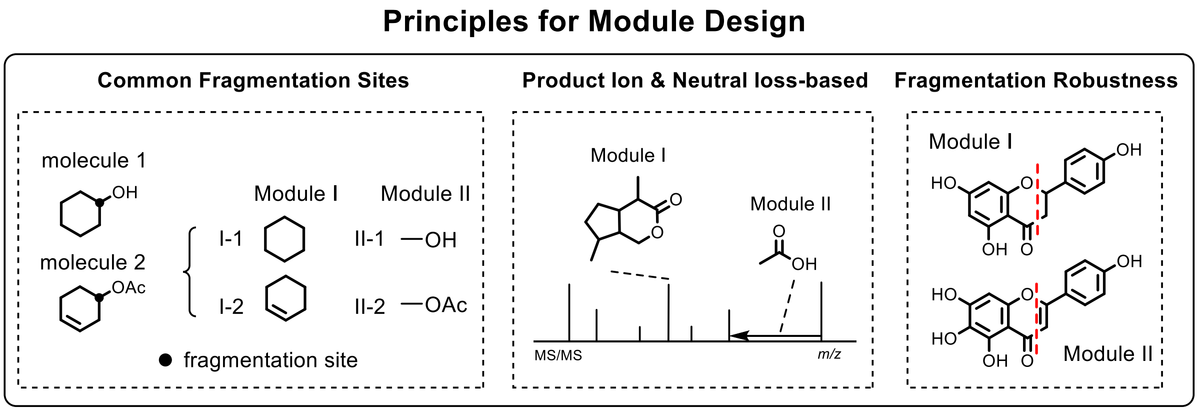

Figure S1. Module design for CNPs.

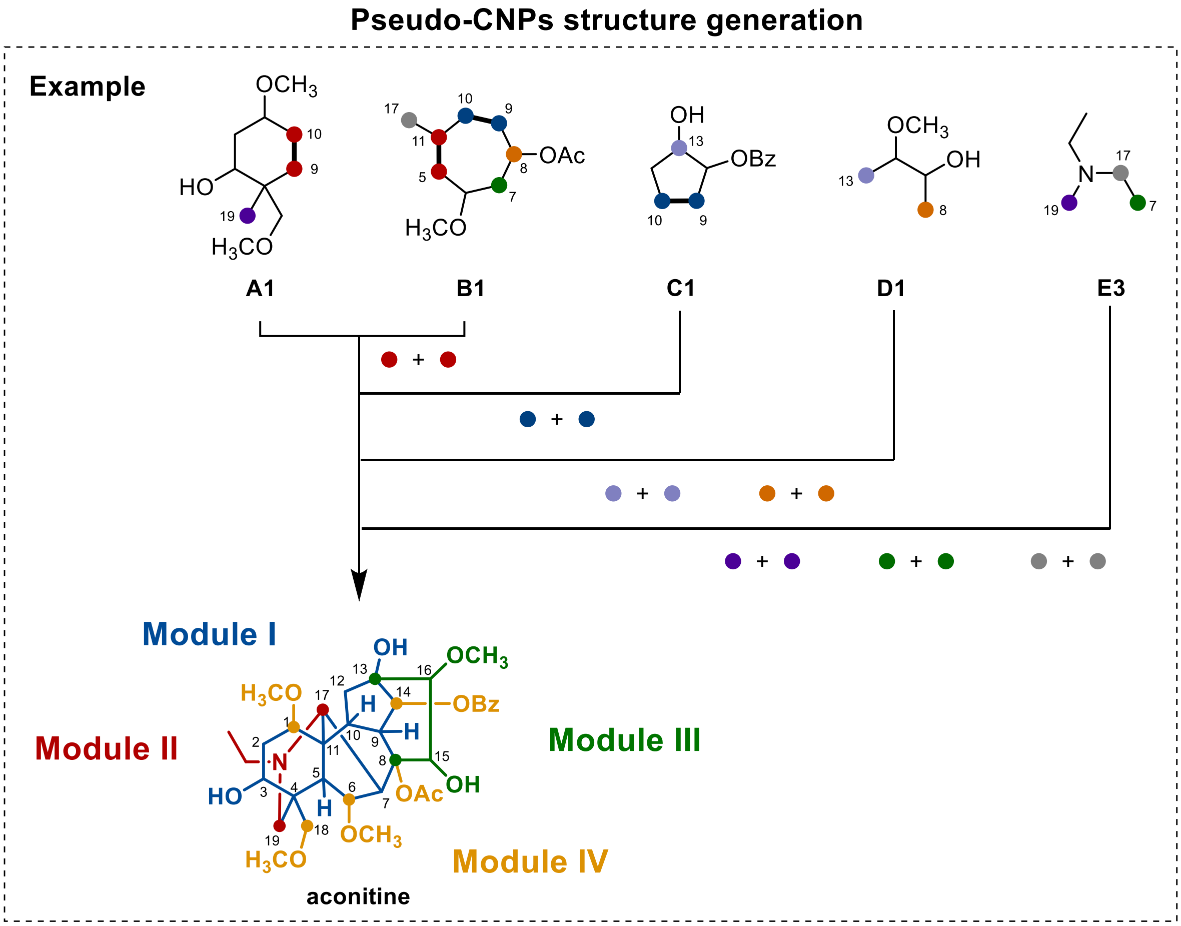

Figure S2. Example of pseudo-CNPs structure generation.

<u>**Product Ion Formulae Prediction of pseudo-CNPs**</u>

a). Establish a mapping between modules and fragments

Each structural module was linked to specific MS/MS features based on fragmentation analysis. For example, module I, which includes the A and C rings of a CNP, corresponds to a diagnostic product ion (ion i), while Module II, encompassing the B ring, was associated with a characteristic neutral loss (loss i). The module-fragments mapping form the basis for modular prediction.

b). Setting initial product ion formula of module templates

Since CNPs with polycyclic structures and highly functionalities, two main types of product ion spectra were commonly observed. In many CNPs cases, such as terpenoids, product ions originating from the core carbon skeleton appear with high frequency and intensity. The composition formulas of highly repetitive, high-intensity carbon skeleton-derived product ions were selected as the initial product ion formula for template modules. In other cases, such as alkaloids, fragmentation of the carbon skeleton results in weaker ion signals, while neutral losses from labile substituents (e.g., acetyl, methoxyl, hydroxyl groups) are more prominent. In such cases, the precursor ion formula was designated as the initial reference formula for product ion prediction.

c). Definition of the mass shift matrix for submodules

Module I

$M^{(1)}$

Type	n_C	n_{II}	n_O
$M_1^{(1)}$	0	0	0
$M_2^{(1)}$	+2	+1	-1
$M_3^{(1)}$	0	+2	0
...	...	...	...
$M_j^{(1)}$	$\Delta m_{M_j^{(1)},C}$	$\Delta m_{M_j^{(1)},H}$	$\Delta m_{M_j^{(1)},O}$

Module II

$M^{(2)}$

Type	n_C	n_{II}	n_O
$M_1^{(2)}$	0	0	0
$M_2^{(2)}$	+1	+2	-2
$M_3^{(2)}$	0	+2	+1
...	...	...	...
$M_j^{(2)}$	$\Delta m_{M_j^{(2)},C}$	$\Delta m_{M_j^{(2)},H}$	$\Delta m_{M_j^{(2)},O}$

Module III

$M^{(3)}$

Type	n_C	n_{II}	n_O
$M_1^{(3)}$	0	0	0
$M_2^{(3)}$	0	+4	-1
$M_3^{(3)}$	0	+2	-1
...	...	...	...
$M_j^{(3)}$	$\Delta m_{M_j^{(3)},C}$	$\Delta m_{M_j^{(2)},H}$	$\Delta m_{M_j^{(3)},O}$

...

Module k

$M^{(k)}$

Type	n_C	n_{II}	n_O
$M_1^{(k)}$	$\Delta m_{M_1^{(k)},C}$	$\Delta m_{M_1^{(k)},H}$	$\Delta m_{M_1^{(k)},O}$
$M_2^{(k)}$	$\Delta m_{M_2^{(k)},C}$	$\Delta m_{M_2^{(k)},H}$	$\Delta m_{M_2^{(k)},O}$
$M_3^{(k)}$	$\Delta m_{M_3^{(k)},C}$	$\Delta m_{M_3^{(k)},H}$	$\Delta m_{M_3^{(k)},O}$
...	...	...	...
$M_j^{(k)}$	$\Delta m_{M_j^{(k)},C}$	$\Delta m_{M_j^{(k)},H}$	$\Delta m_{M_j^{(k)},O}$

Figure S3. Definition of the mass shift matrix for all $\boldsymbol{Module\ k}$.

Supposing the target-CNP was divided into k types of module, and defined as M^k, each module consisted of j types submodules, defined as M_j^k. The mass shifts Δm of elements carbon (C), hydrogen (H), oxygen (O), and nitrogen (N) which generated by each submodule were defined as $\Delta m_{M_j^k,C}, \Delta m_{M_j^k,H}, \Delta m_{M_j^k,O}, \Delta m_{M_j^k,N}$, respectively. The mass shift matrix for the submodules of each module was defined as $\Delta m_{M_j^k}$:

$$\Delta m_{M_j^k} = \begin{pmatrix} \Delta m_{M_1^k,C} & \Delta m_{M_1^k,H} & \Delta m_{M_1^k,O} & \Delta m_{M_1^k,N} \\ \Delta m_{M_2^k,C} & \Delta m_{M_2^k,H} & \Delta m_{M_2^k,O} & \Delta m_{M_2^k,N} \\ \vdots & \vdots & \vdots & \vdots \\ \Delta m_{M_j^k,C} & \Delta m_{M_j^k,H} & \Delta m_{M_j^k,O} & \Delta m_{M_j^k,N} \end{pmatrix}$$

(a) All combinations of M_j^k

Each M^k can select any submodule M_j^k and all possible combinations formed the Cartesian product $\mathcal{C}$:

$$\mathcal{C} = \prod_{k=1}^{k} M_j^k$$

which can be expanded as: $\mathcal{C} = M_j^1 \times M_j^2 \times \cdots \times M_j^k$

The total number of combinations was: Total number of combinations $= \prod_{k=1}^{k} j$

(b) Total mass shift vector for any combination:

For any combination $\mathcal{C}_i$ can be expressed as,

$$\mathcal{C}_i = \left(M_i^1, M_i^2, \ldots, M_i^k\right), i \in \{1,2,\ldots,\prod_{k=1}^{k} j\}$$

the total mass shift vector $\Delta m_{total,i}$:

$$\Delta m_{total,i} = \sum_{k=1}^{k} \Delta m_{M_i^k}$$

Expanding as:

$$\Delta m_{\text{total},i} = \begin{bmatrix} \sum_{k=1}^{k} \Delta m_{M_i^{(k)},C} \\ \sum_{k=1}^{k} \Delta m_{M_i^{(k)},H} \\ \sum_{k=1}^{k} \Delta m_{M_i^{(k)},O} \\ \sum_{k=1}^{k} \Delta m_{M_i^{(k)},N} \end{bmatrix}$$

(c) Predicted mass vector for each combination:

Assuming the initial mass vector of the characteristic product ion was m_0

$$m_0 = \begin{bmatrix} m_{C,0} \\ m_{H,0} \\ m_{O,0} \\ m_{N,0} \end{bmatrix}$$

For any combination $\mathcal{C}_i$, the predicted mass vector $m_{predict,i}$ was given by:

$$m_{predict,i} = m_0 + \Delta m_{total,i}$$

Expanding as:

$$m_{predict,i} = m_0 + \sum_{k=1}^{k} \Delta m_{M_i^k}, i \in \{1,2,\ldots,\prod_{k=1}^{k} j\}$$

Further expanding as:

$$m_{\text{predict},i} = \begin{bmatrix} m_{C,0} + \sum_{k=1}^{k} \Delta m_{M_i,C} \\ m_{H,0} + \sum_{k=1}^{k} \Delta m_{M_i,H} \\ m_{O,0} + \sum_{k=1}^{k} \Delta m_{M_i,O} \\ m_{N,0} + \sum_{k=1}^{k} \Delta m_{M_i,N} \end{bmatrix}$$

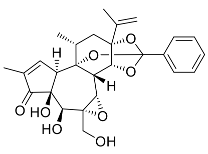

DA01

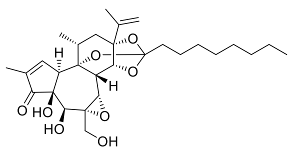

DA02

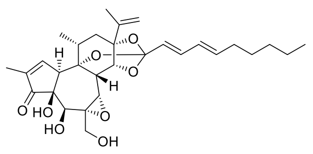

DA03

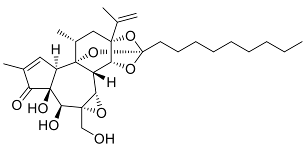

DA04

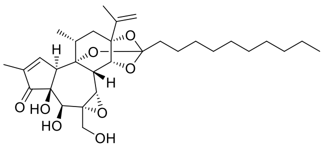

DA05

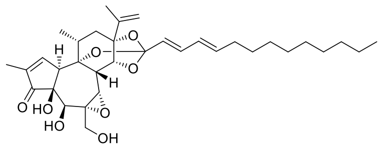

DA06

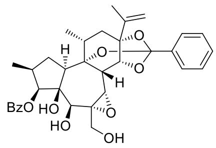

DA07

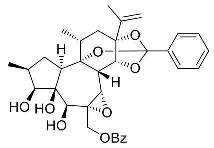

DA08

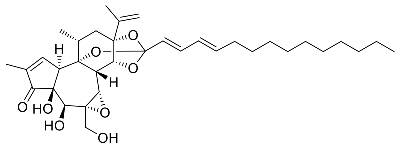

DA09

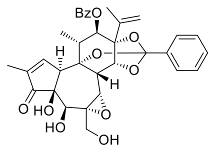

DA10

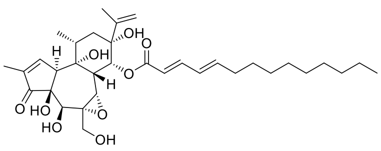

DA11

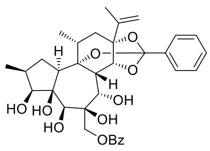

DA12

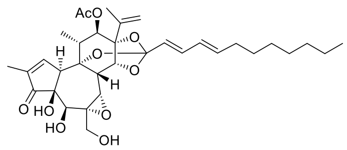

DA13

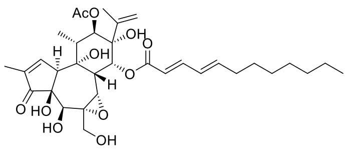

DA14

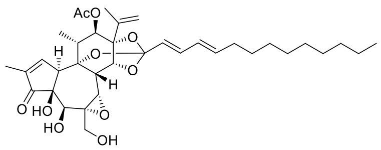

DA15

Figure S4. **Structures of 28 normal daphnane-type diterpenoids.**

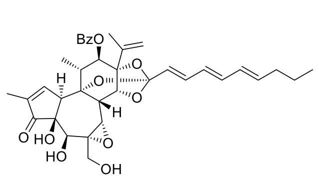

DA16

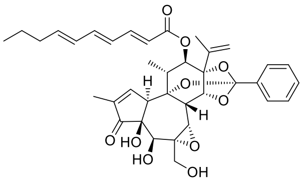

DA17

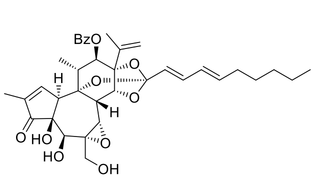

DA18

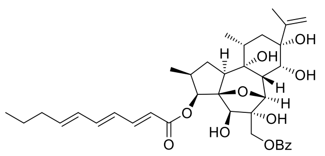

DA19

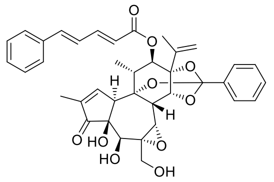

DA20

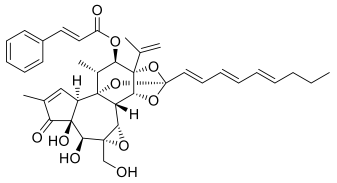

DA21

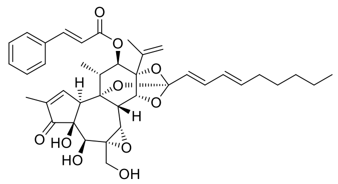

DA22

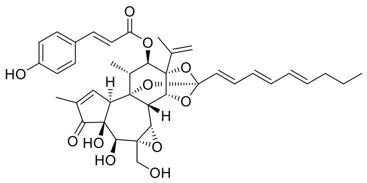

DA23

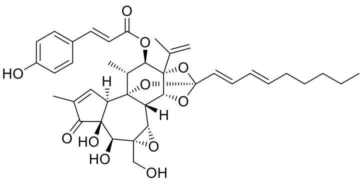

DA24

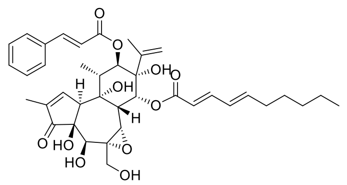

DA25

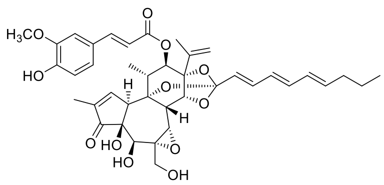

DA26

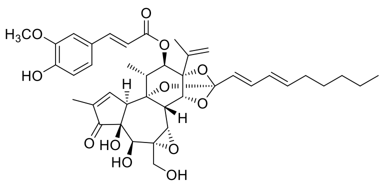

DA27

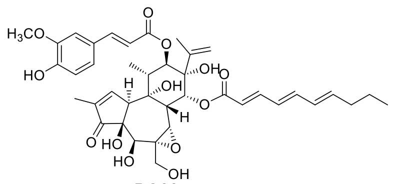

DA28

Figure S4. **Structures of 28 normal daphnane-type diterpenoids (continued).**

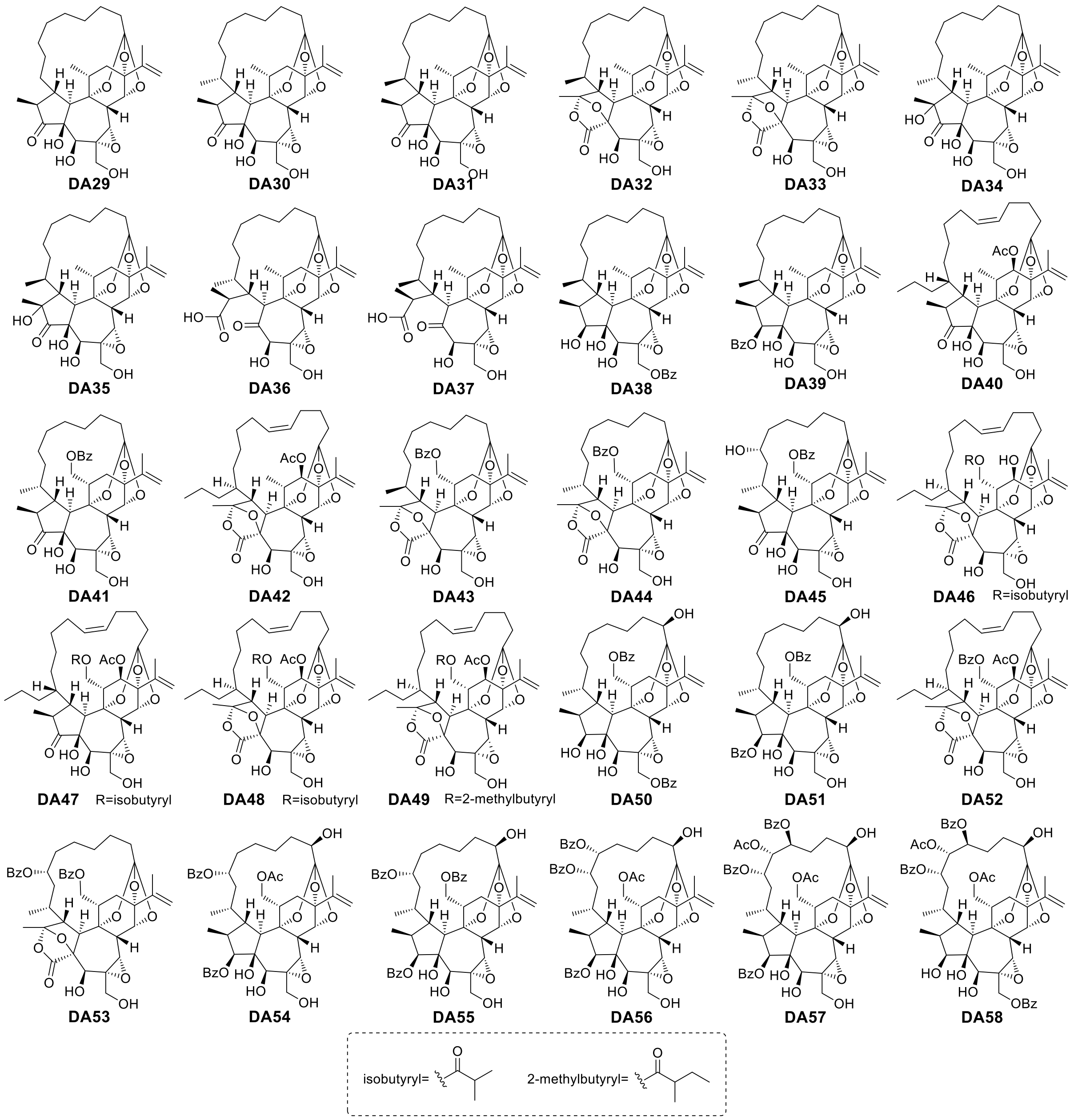

Figure S5. **Structures of 30 macrocyclic daphnane othroesters.**

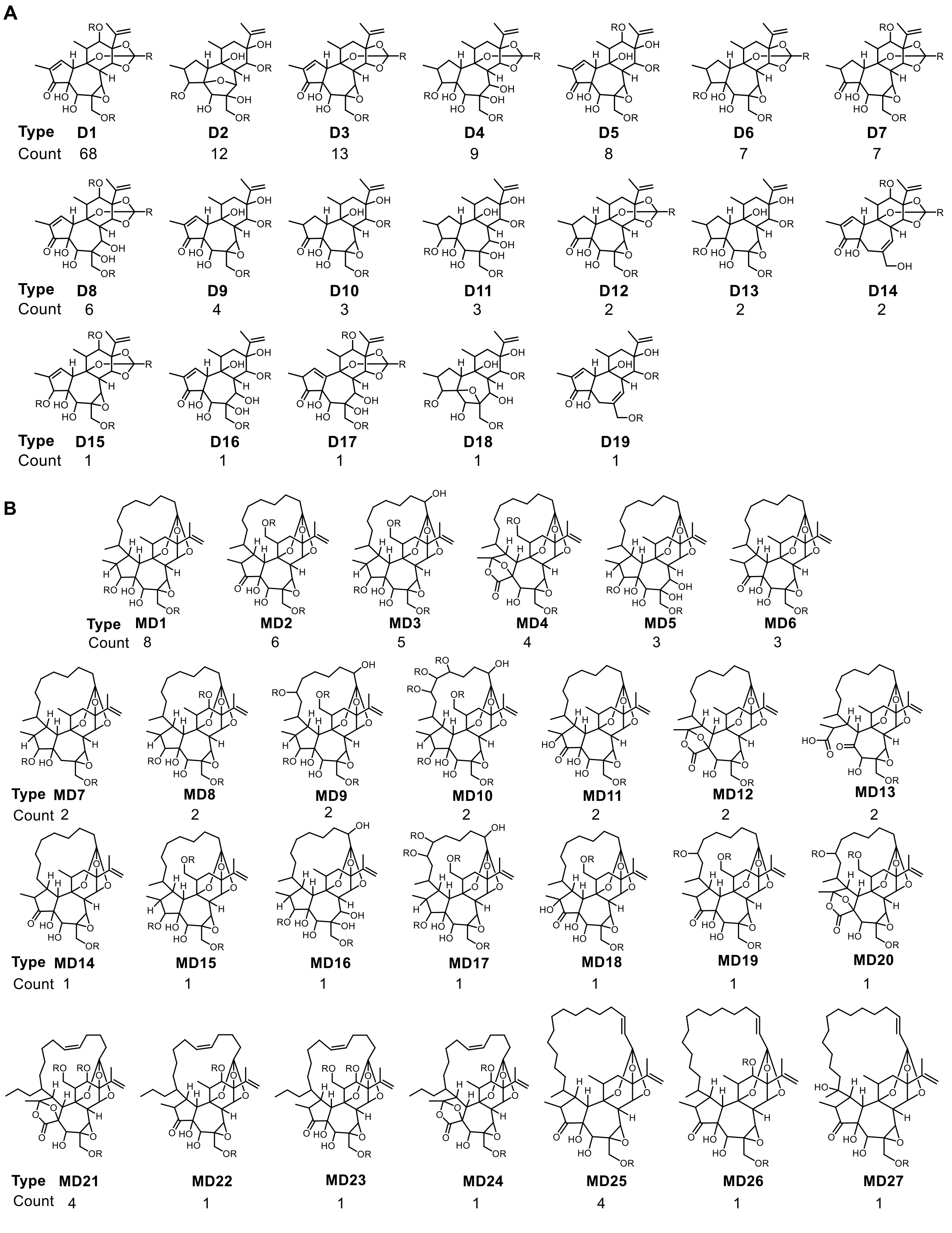

Figure S6. **Oxidation skeletons of daphnanes in Thymelaeaceae family.** (A) 151 Normal daphnane with 19 oxidation skeletons and number of reported compounds in Thymelaeaceae family; (B) 63 macrocyclic daphnane othroesters with 27 oxidation skeletons and number of reported compounds in Thymelaeaceae family (Until Feb 2025).

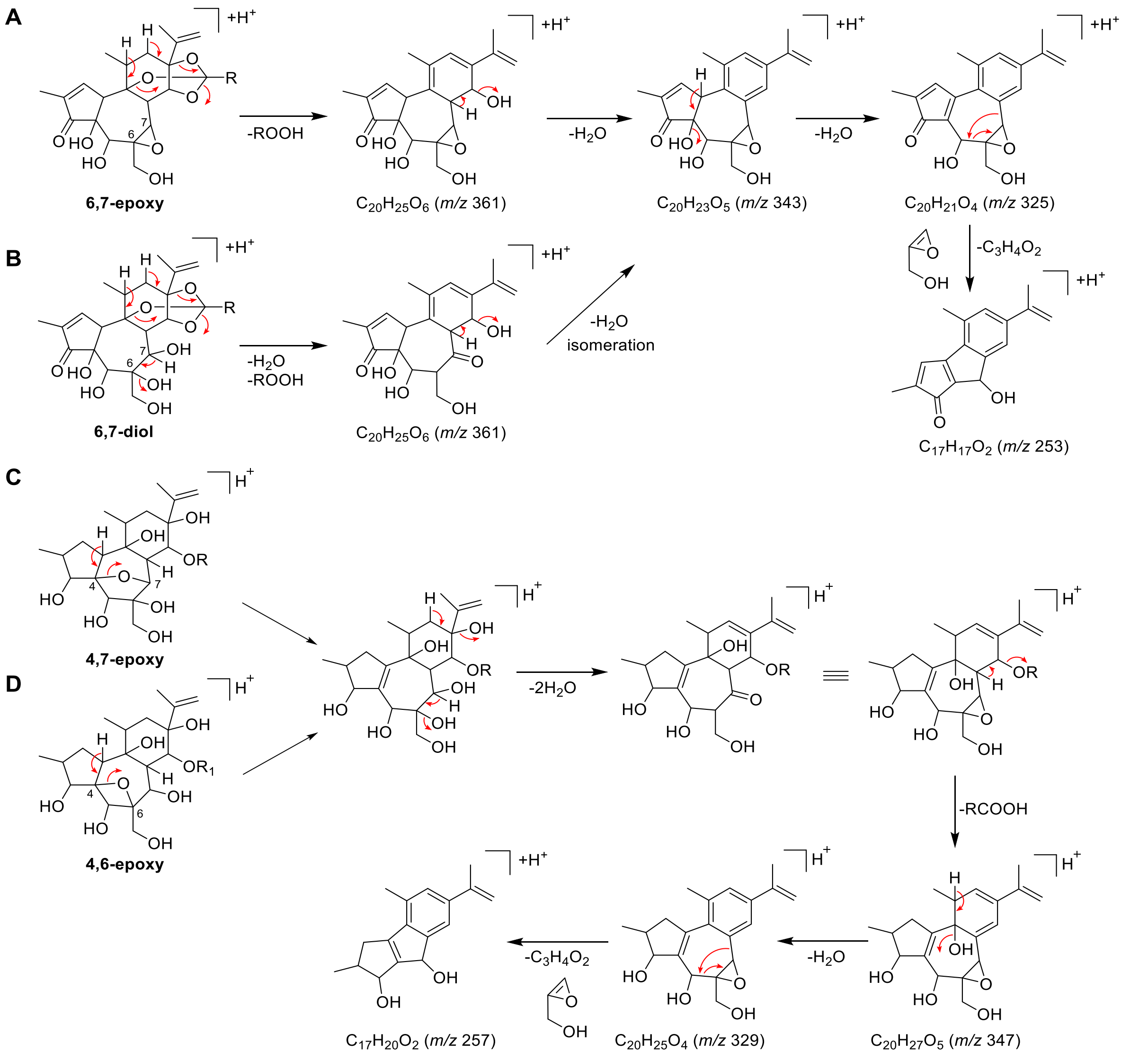

Figure S7. Proposal characteristic ESI-MS/MS fragmentation pathway of daphnanes with different B ring structure. (A) 6,7-epoxy (B) 6,7-diol (C) 4,7-epoxy (D) 4,6-epoxy.

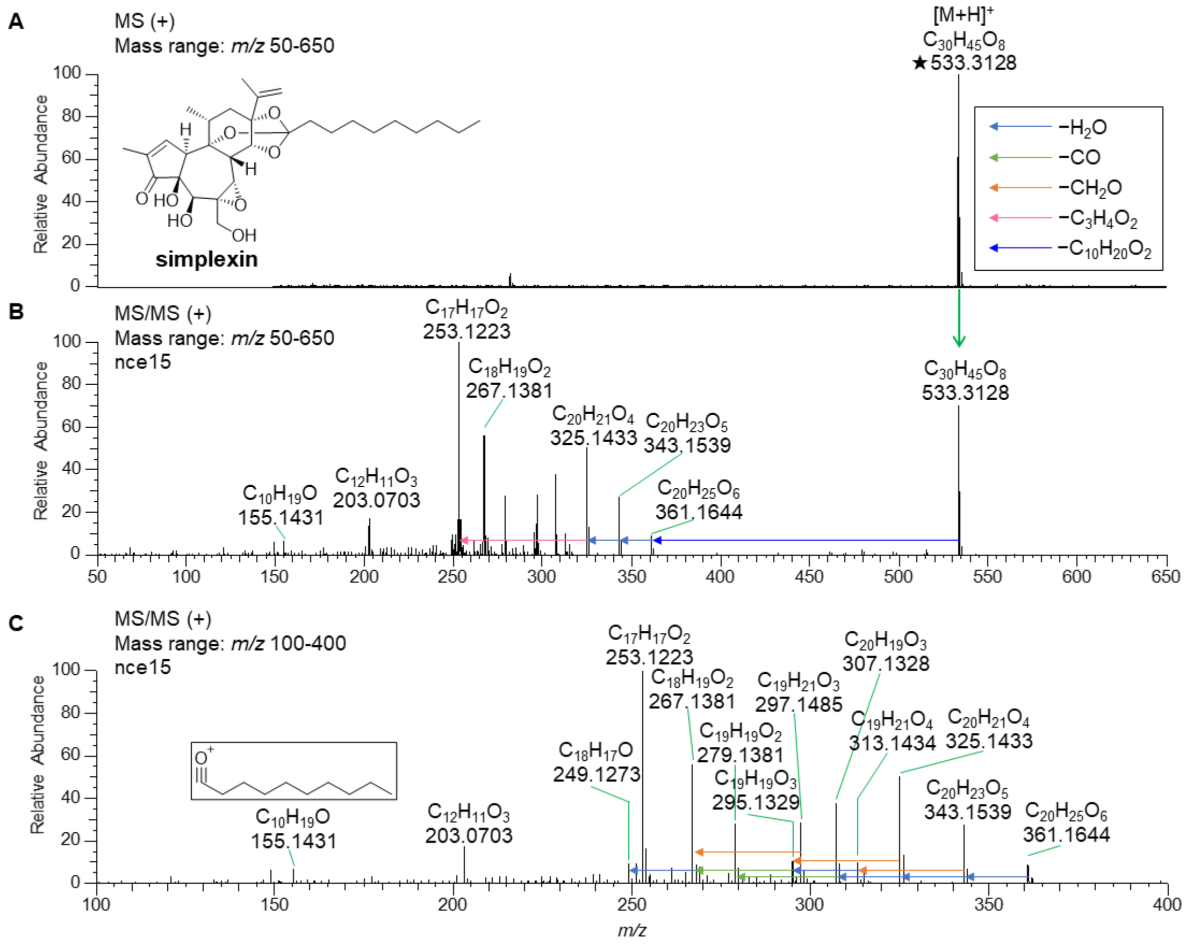

Figure S8. **MS spectra of simplexin.** (A) MS and (B) MS/MS spectra with mass range *m/z* 50–650 (C) MS /MS spectra with mass range of *m/z* 100–400 of simplexin (**DA04**) obtained from the protonated molecular ion peak in positive ion mode.

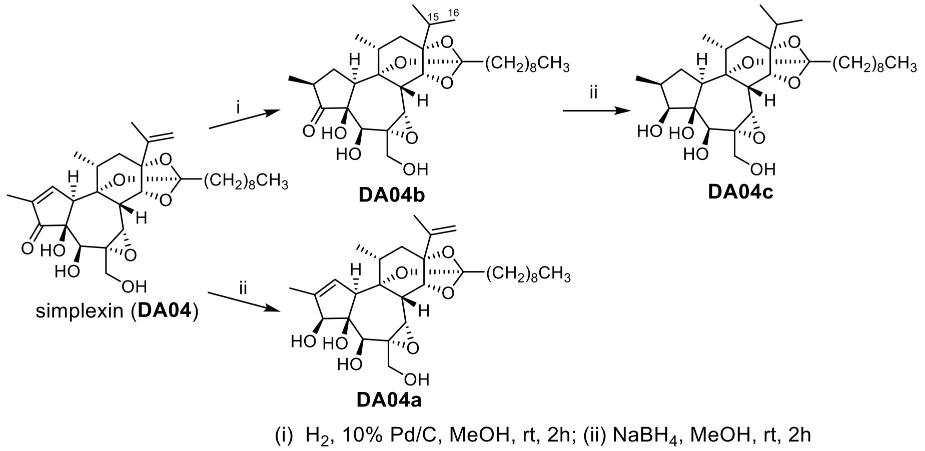

Figure S9. **Synthesis of simplexin derivatives DA04a−DA04c with different A ring structures.**

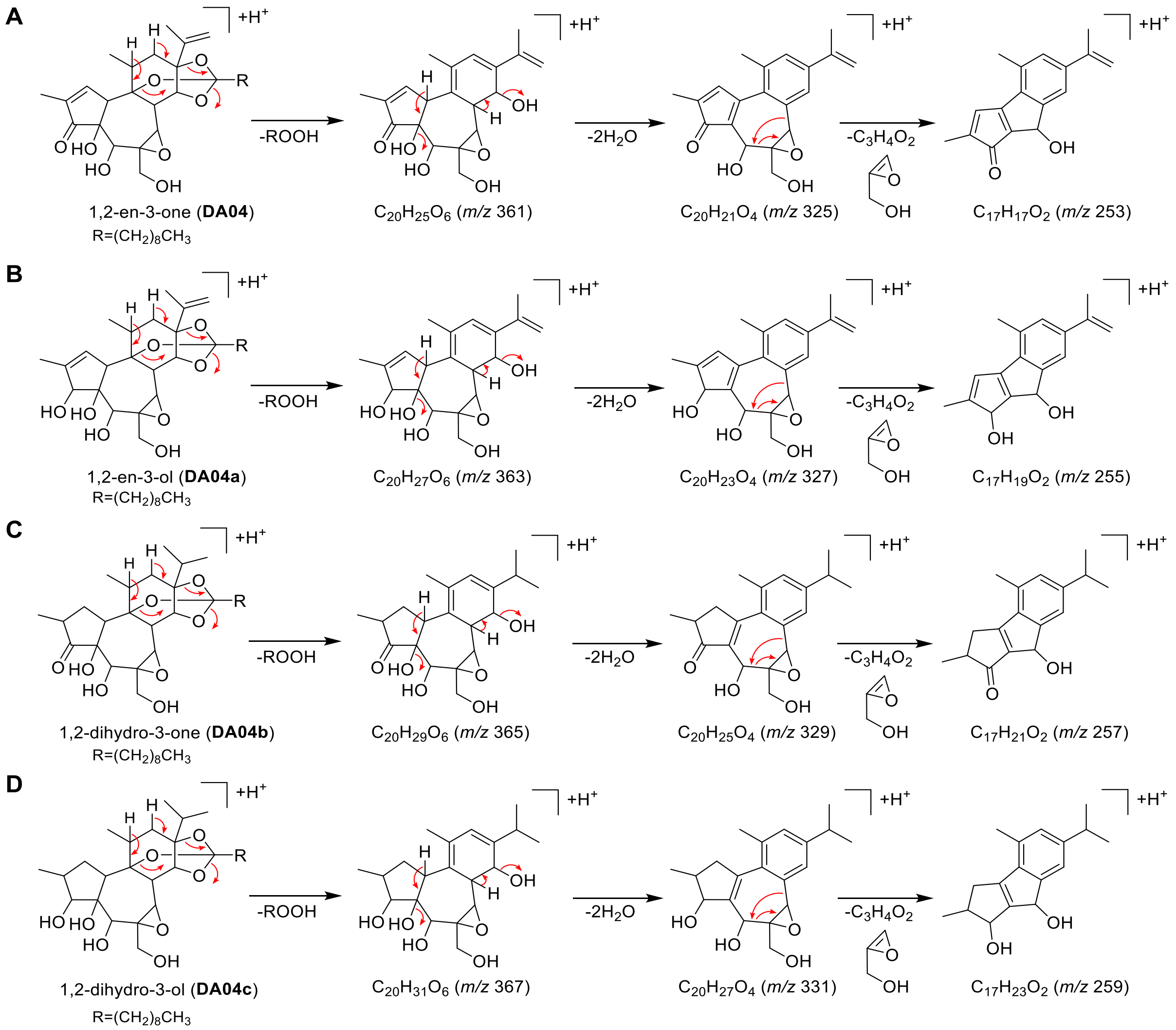

Figure S10. **Proposal characteristic ESI-MS/MS fragmentation pathway of non-macrocyclic daphnanes with different A ring structure.** (A) 1,2-en-3-one (B) 1,2-en-3-ol (C) 1,2-dihydro-3-one (D) 1,2-dihydro-3-ol.

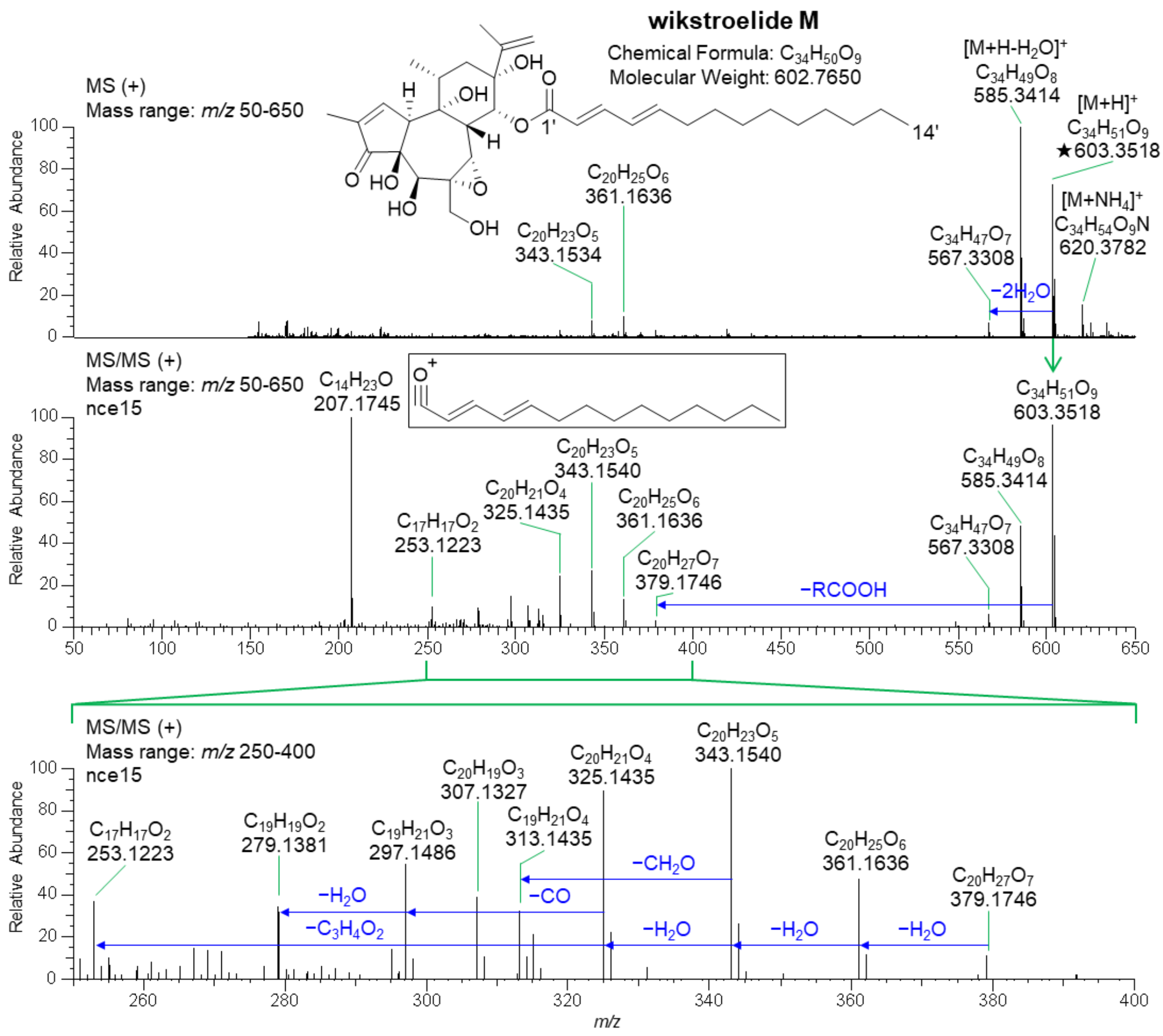

Figure S11. **MS and MS/MS spectra of wikstroelide M (DA11) obtained from the protonated molecular ion peak in positive ion mode.**

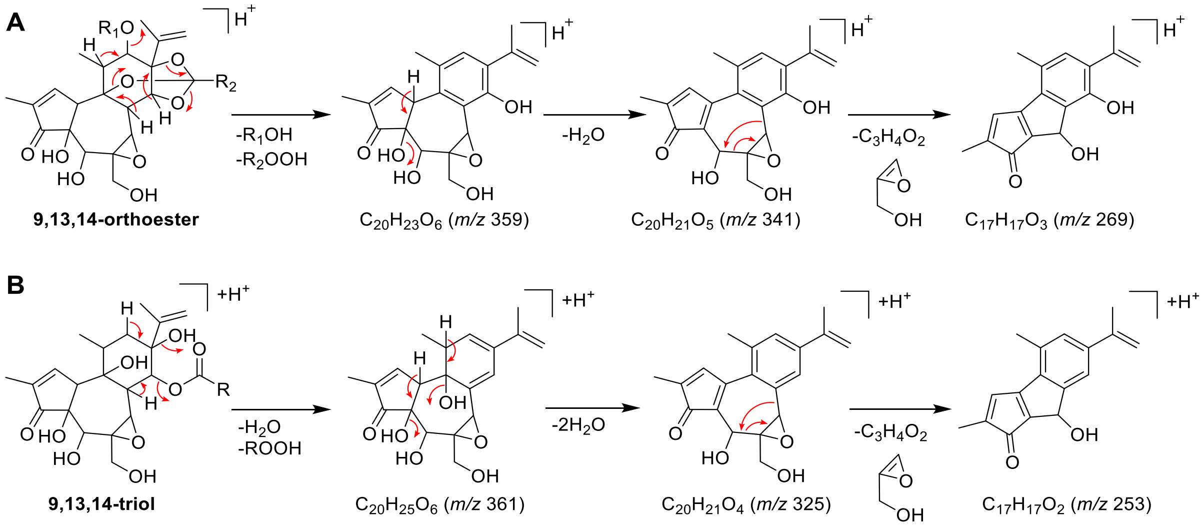

Figure S12. **Proposal characteristic ESI-MS/MS fragmentation pathway of normal daphnanes with different C ring structures.** (A) 9,13,14-orthoester and (B) 9,13,14-triol.

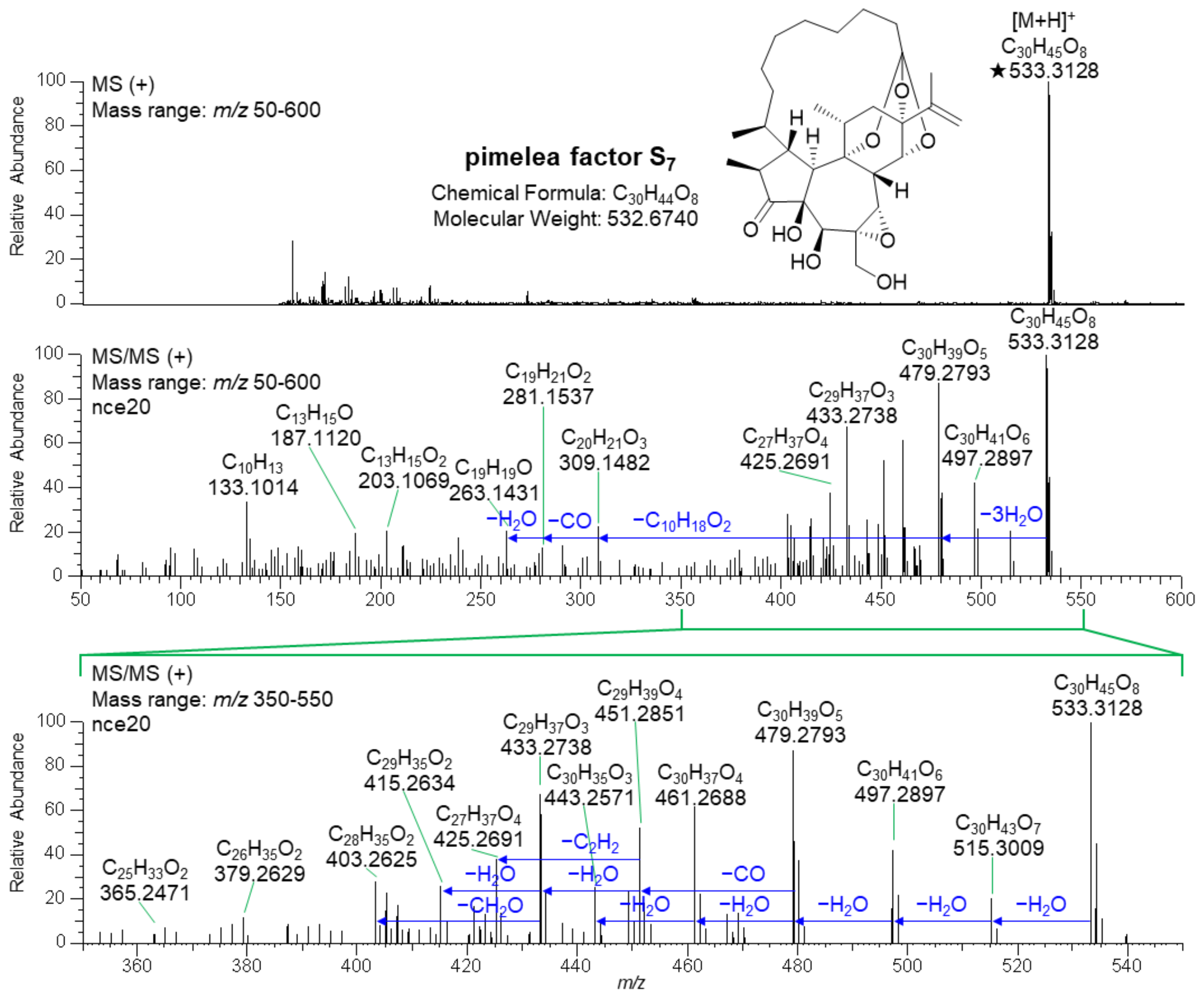

Figure S13. **MS and MS/MS spectra of pimelea factor S7 (DA30) obtained from the protonated molecular ion peak in positive ion mode.**

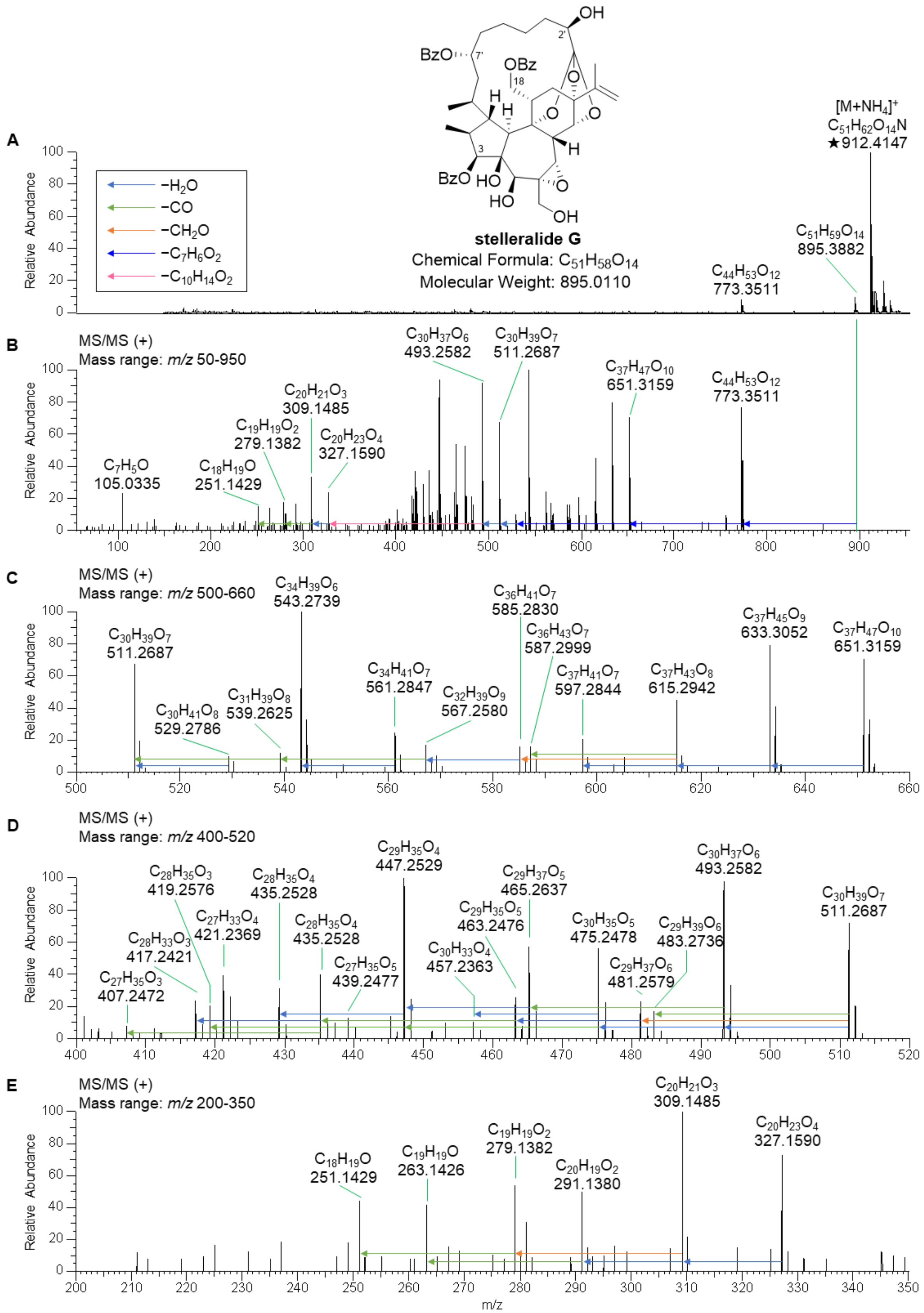

Figure S14. MS and MS/MS spectra of stelleralide G (DA55) obtained from the protonated molecular ion peak in positive ion mode.

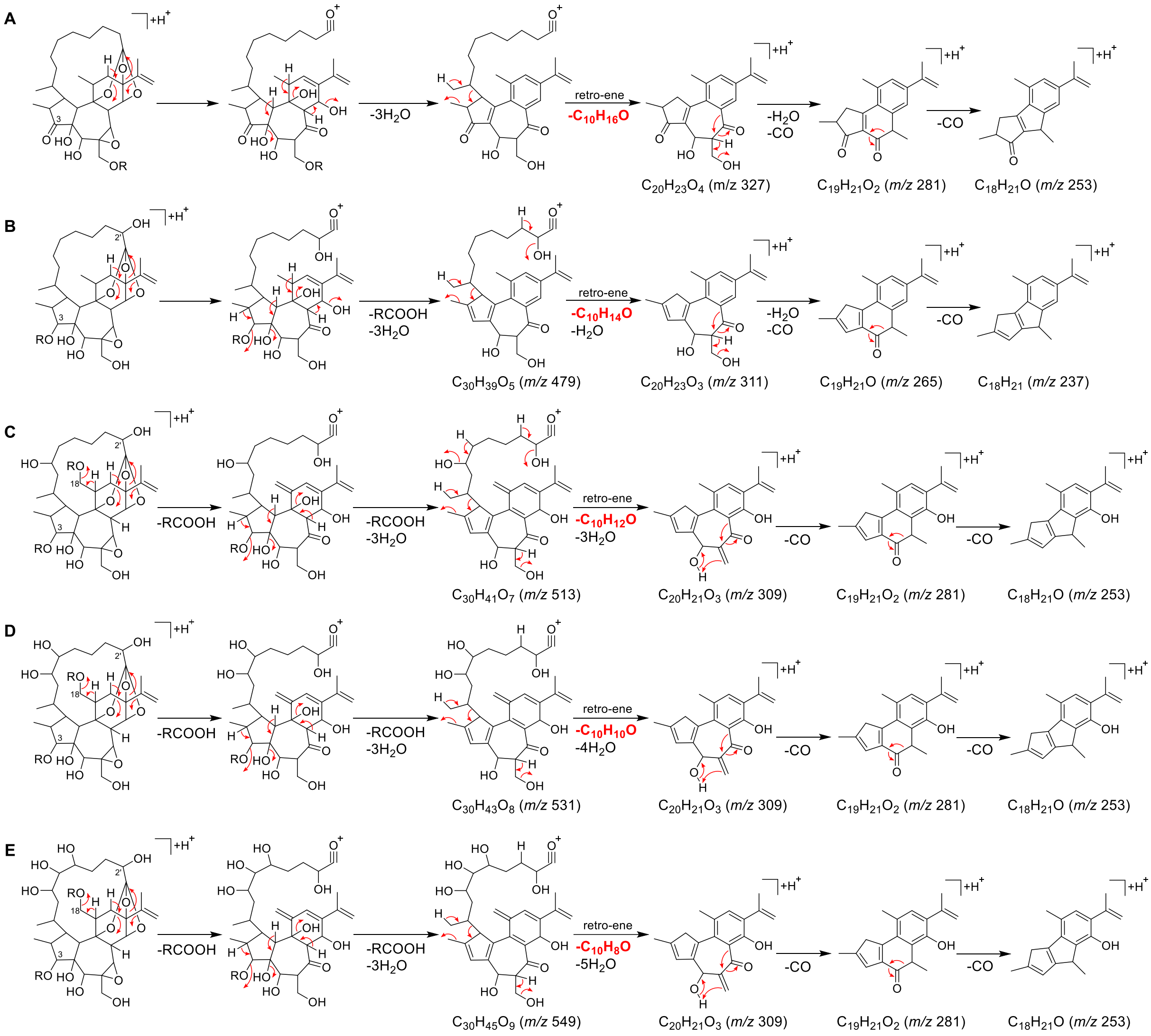

Figure S15. **Proposal characteristic ESI-MS/MS fragmentation pathway of macrocyclic daphnanes with different C_{10} neutral loss.** (A) $C_{10}H_{16}O$ (B) $C_{10}H_{14}O$ (C) $C_{10}H_{12}O$ (D) $C_{10}H_{10}O$ (E) $C_{10}H_{8}O$.

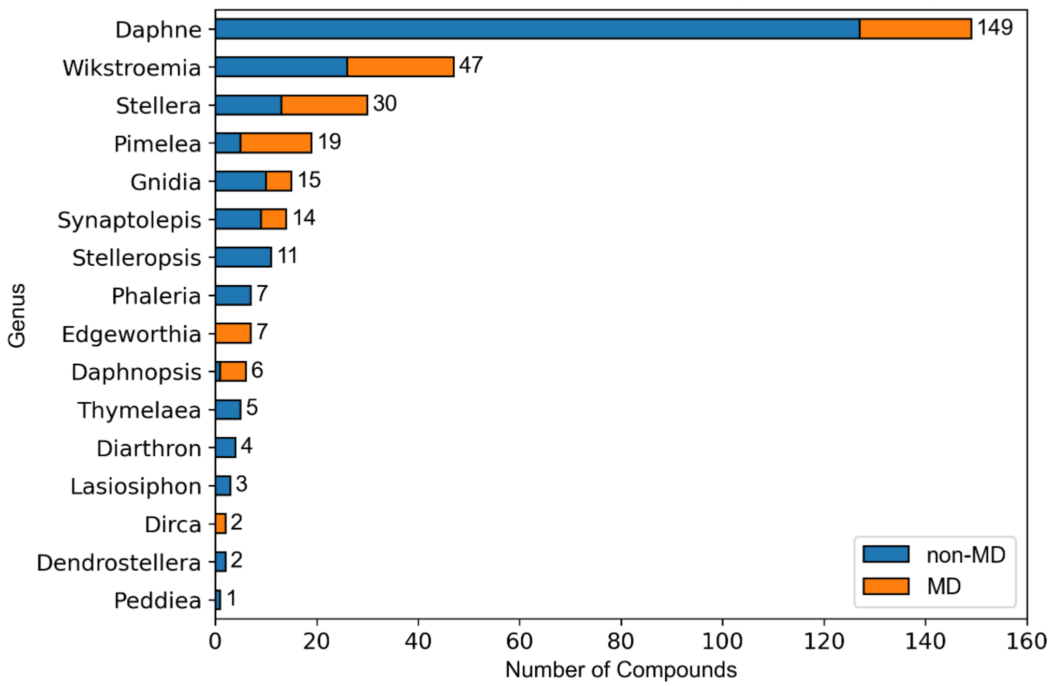

Figure S16. **Distribution of daphnane diterpenoids in Thymelaeaceae family.**

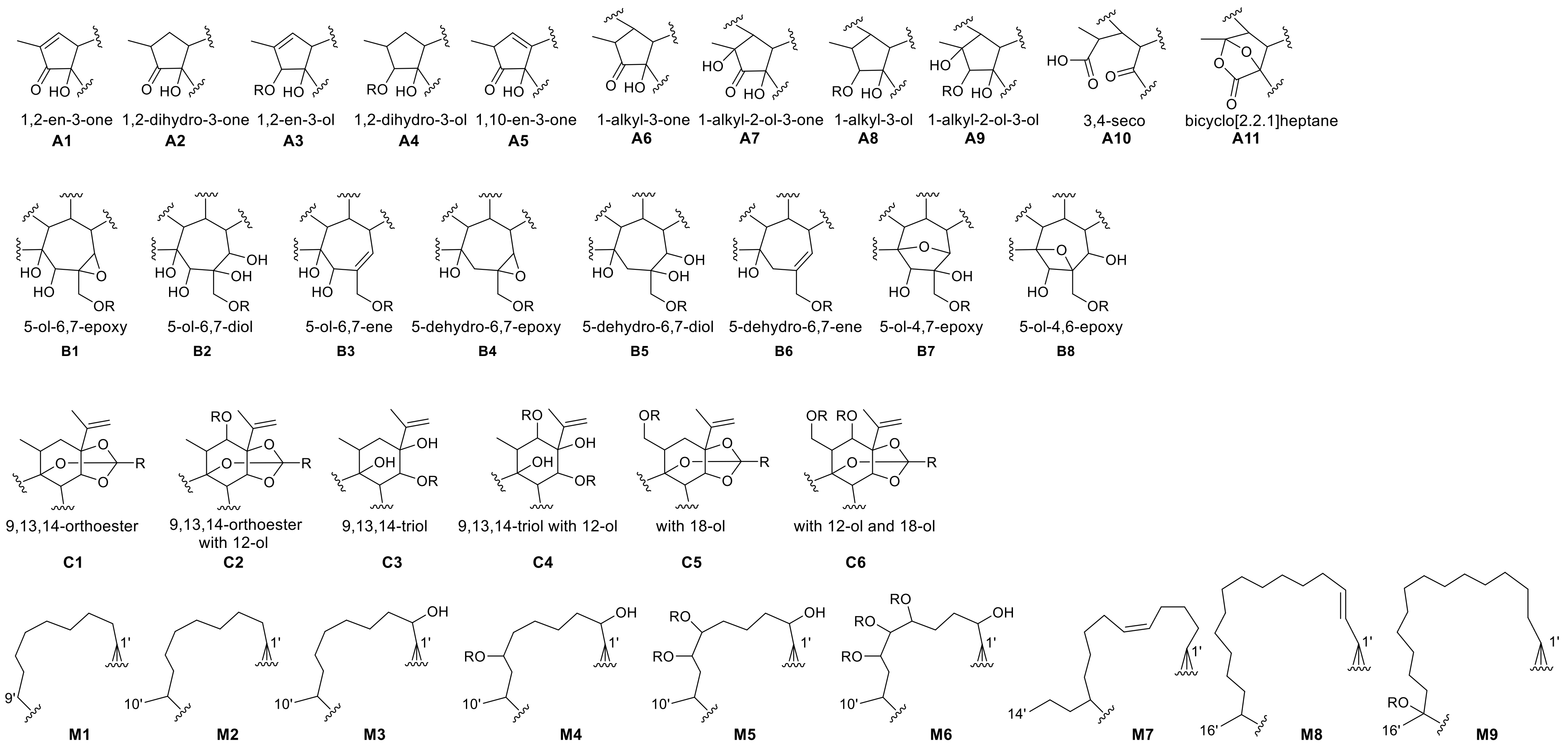

Figure S17. **Skeleton substructure types of daphnane diterpenoids.**

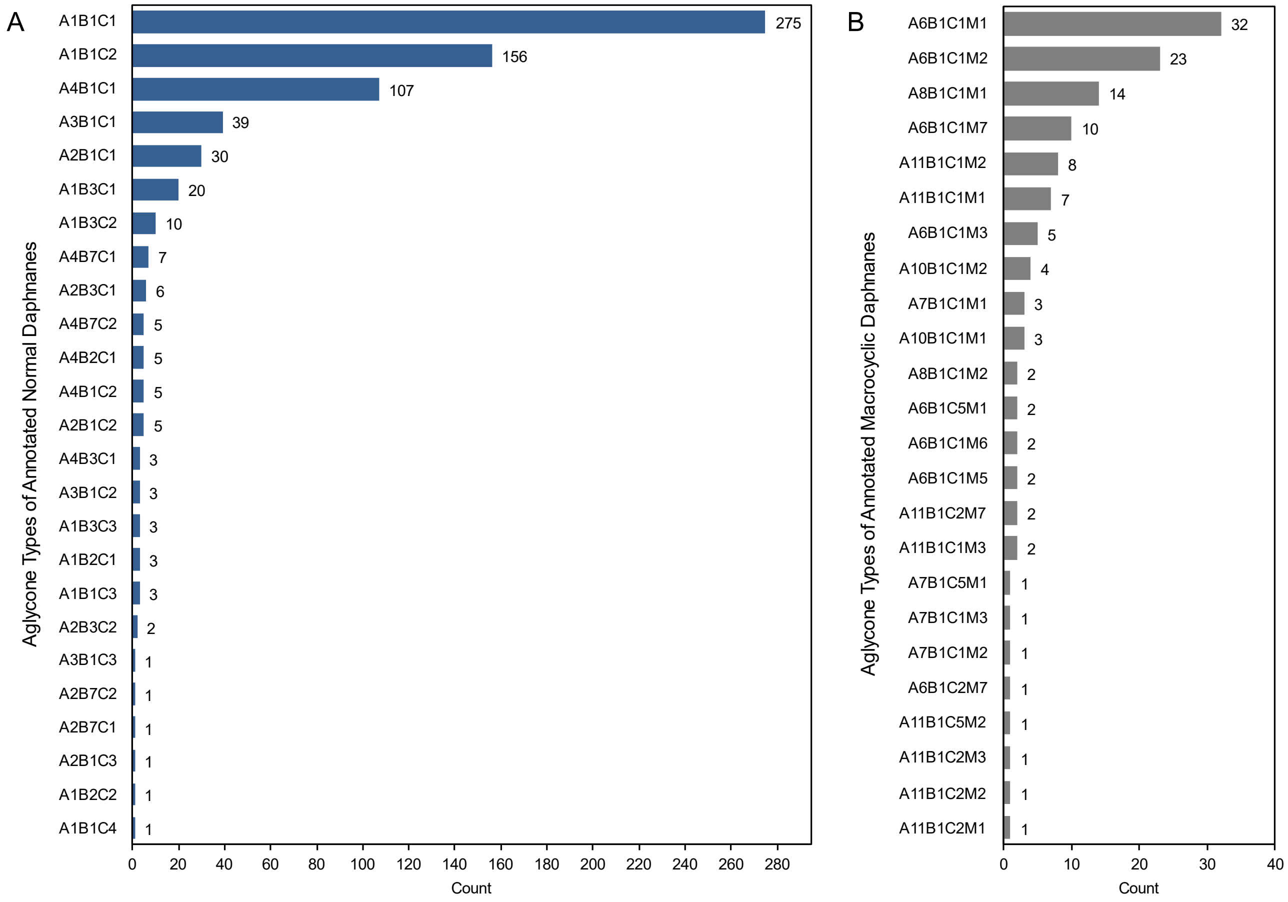

Figure S18. Aglycone types of annotated daphnanes. (A) Normal daphnane-type diterpenoids and (B) macrocyclic daphnane orthoesters.

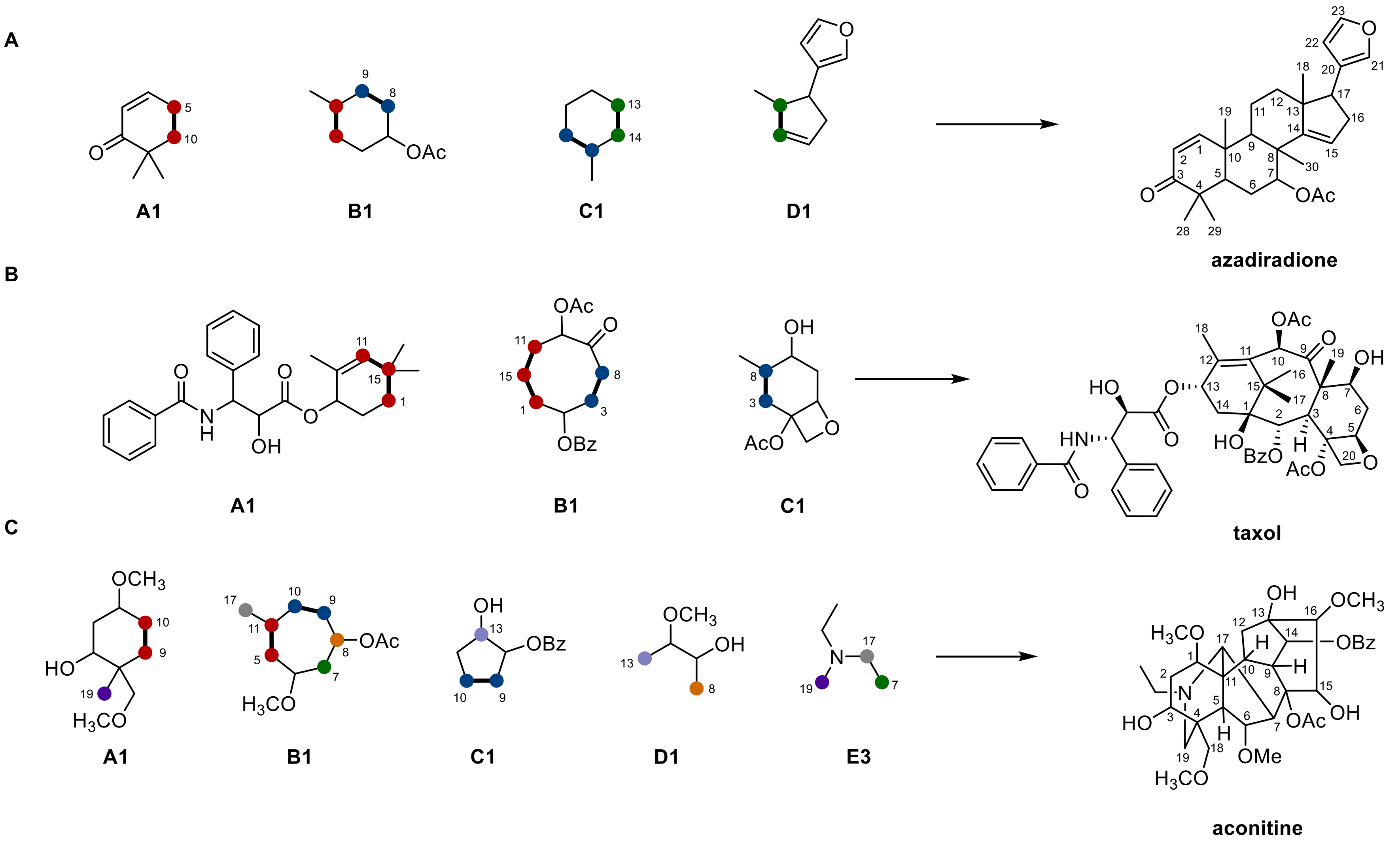

Figure S19. Skeleton substructure types of azadiradione (A), taxol (B), and aconitine (C).

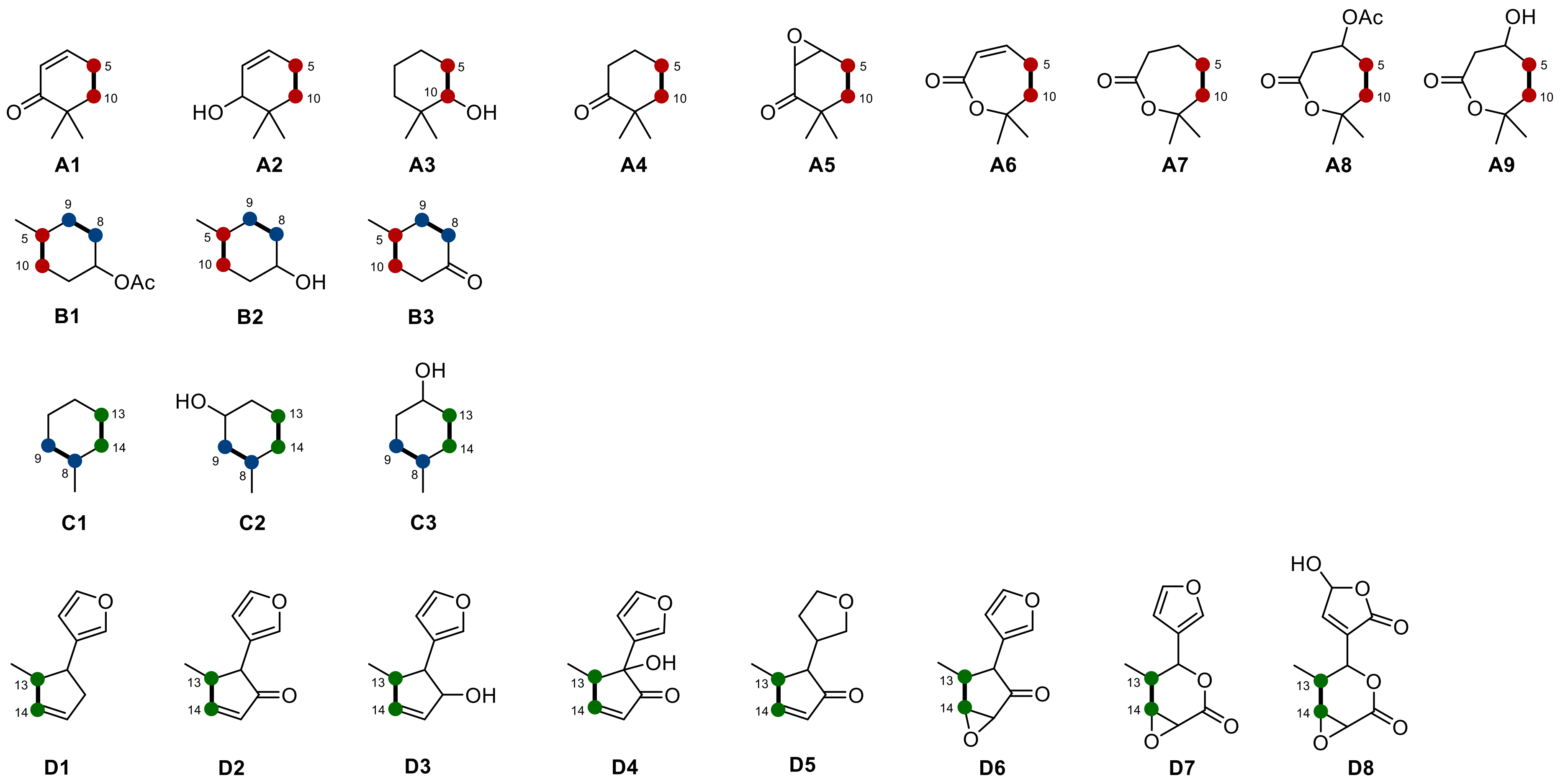

Figure S20. Skeleton substructure types of A,D-seco limonoid-type triterpenoids.

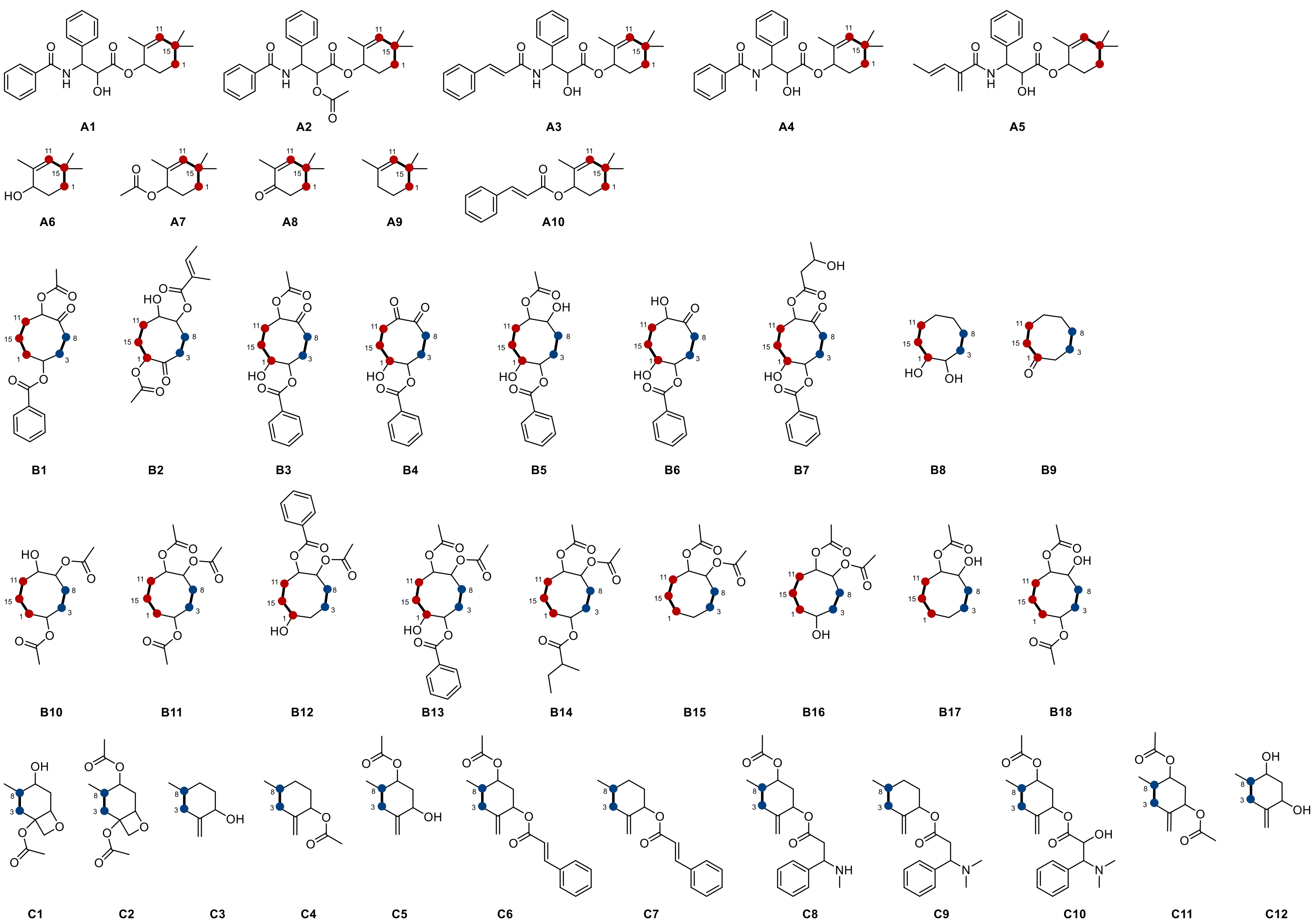

Figure S21. Skeleton substructure types of taxane-type diterpenoids.

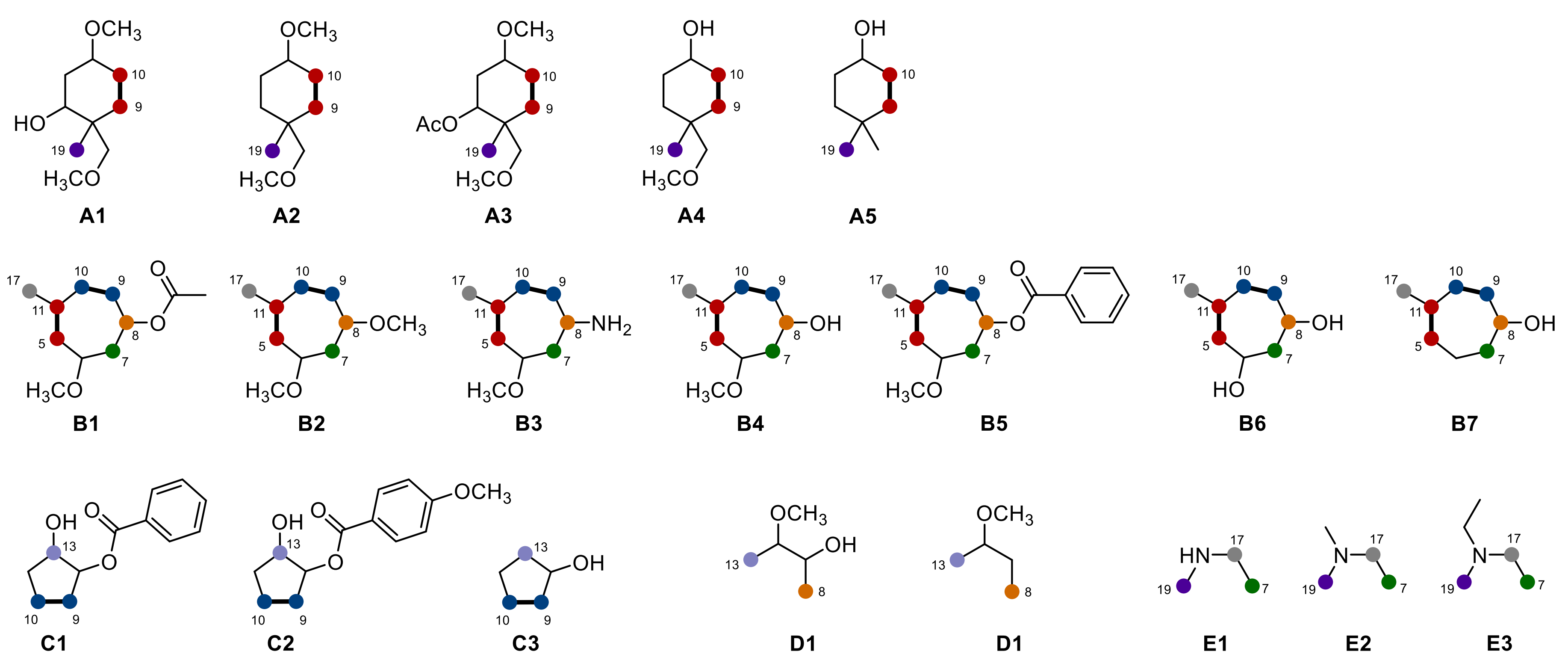

Figure S22. Skeleton substructure types of aconitine-type alkaloids.

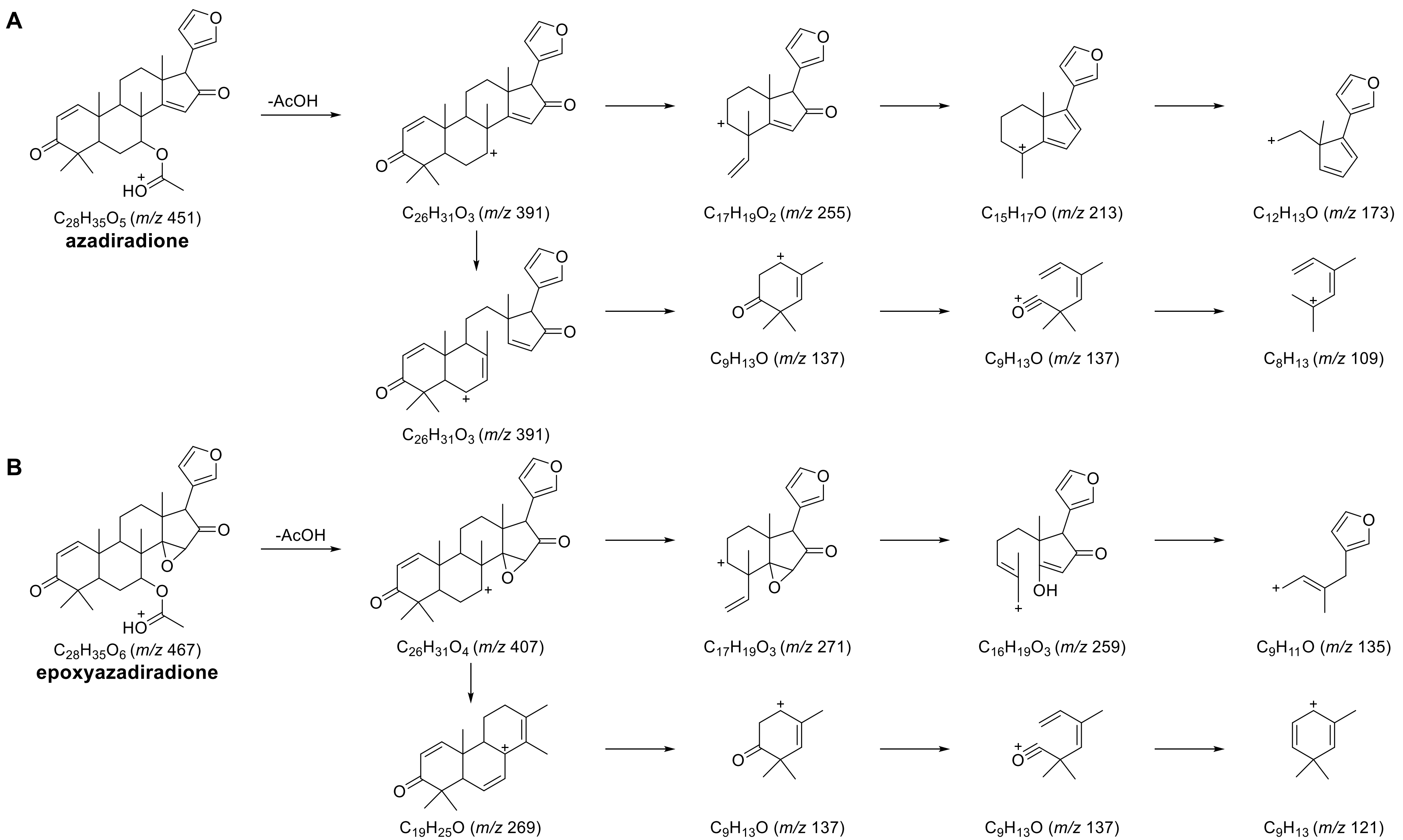

Figure S23. **Proposal characteristic ESI-MS/MS fragmentation pathway of A,D-*seco* limonoid-type triterpenoids.** (A) Azadiradione and (B) epoxyazadiradione.

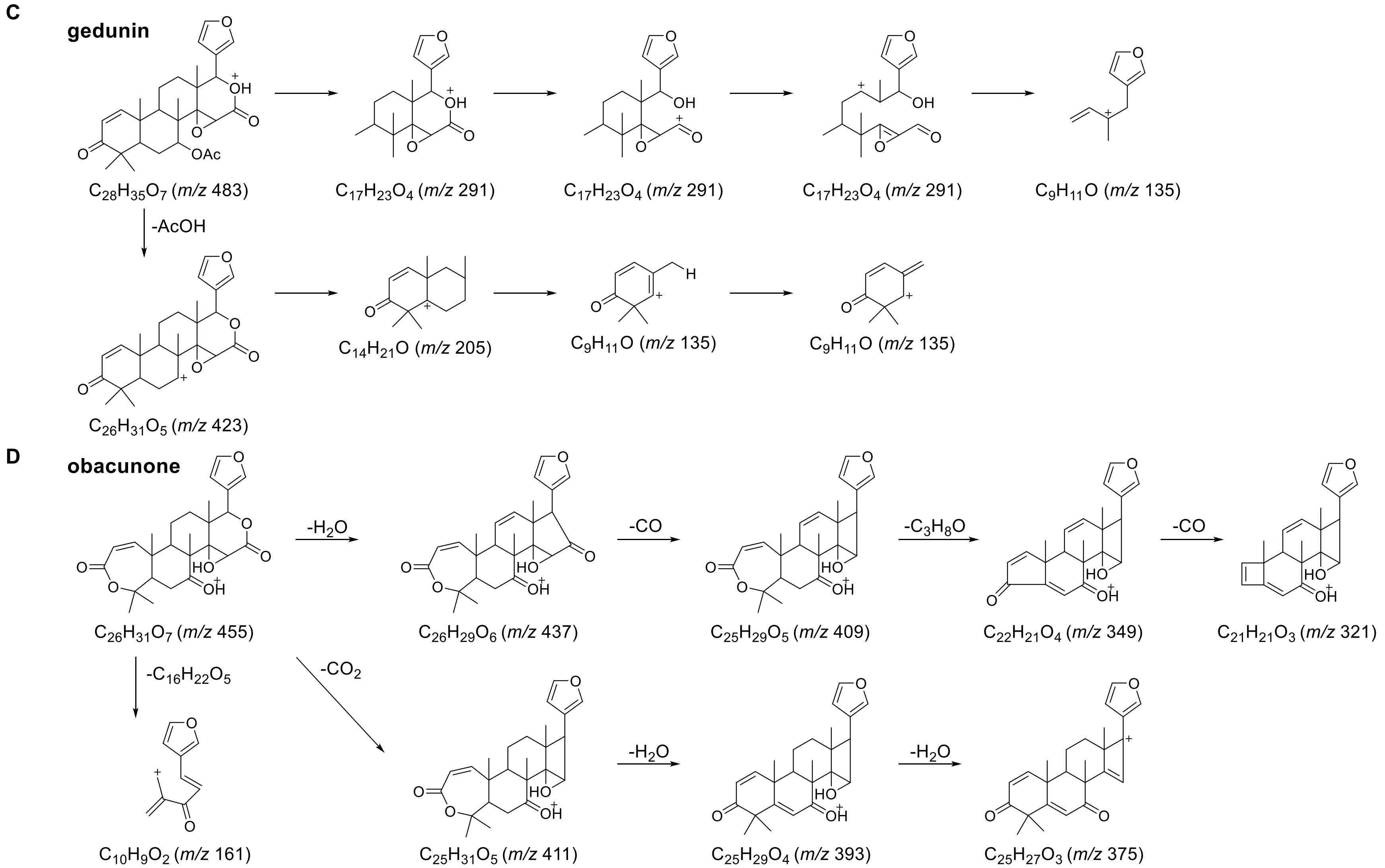

Figure S24. **Proposal characteristic ESI-MS/MS fragmentation pathway of A,D-*seco* limonoid-type triterpenoids.** (C) Gedunin and (D) obacunone.

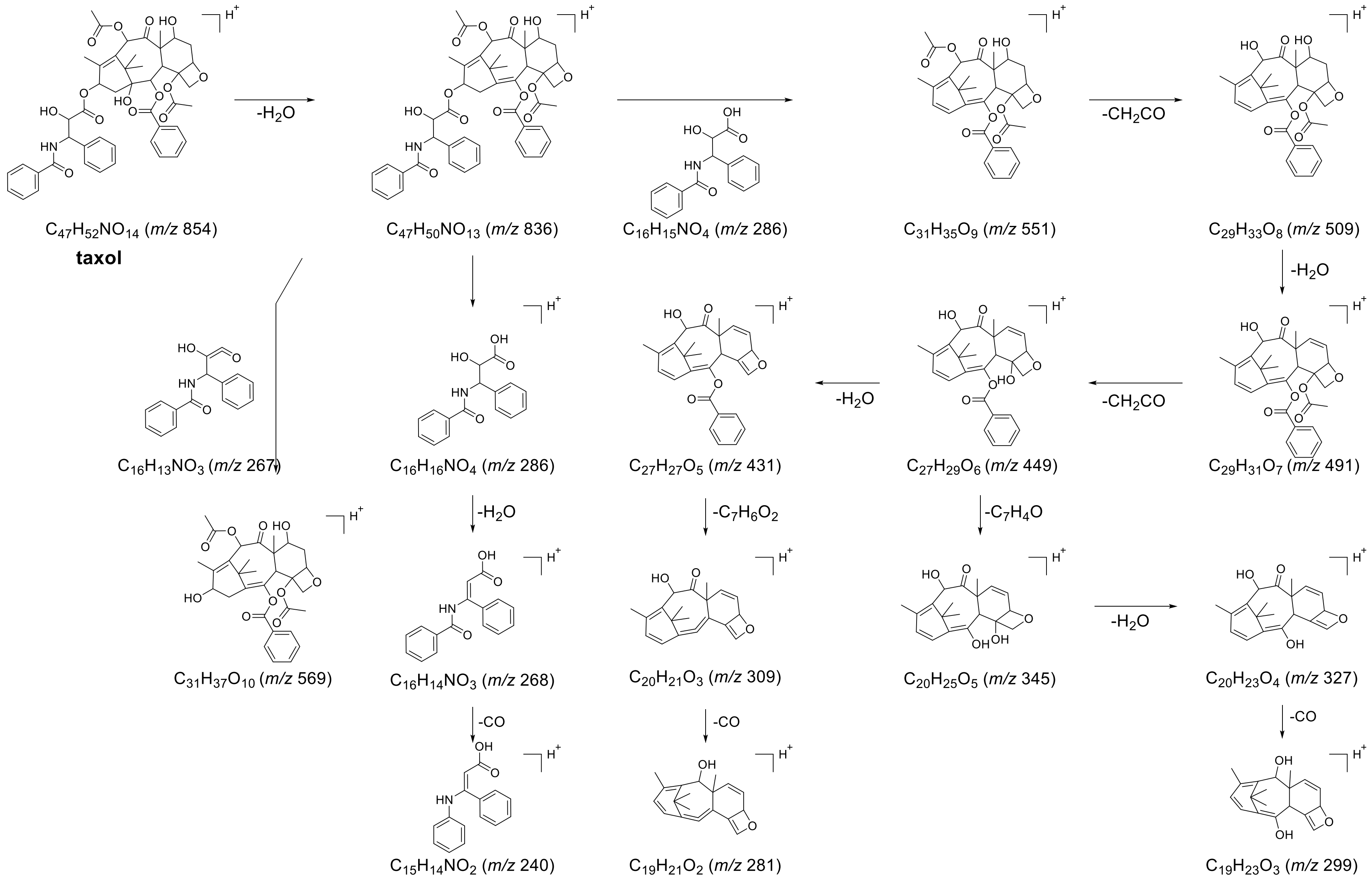

Figure S25. Proposal characteristic ESI-MS/MS fragmentation pathway of taxol.

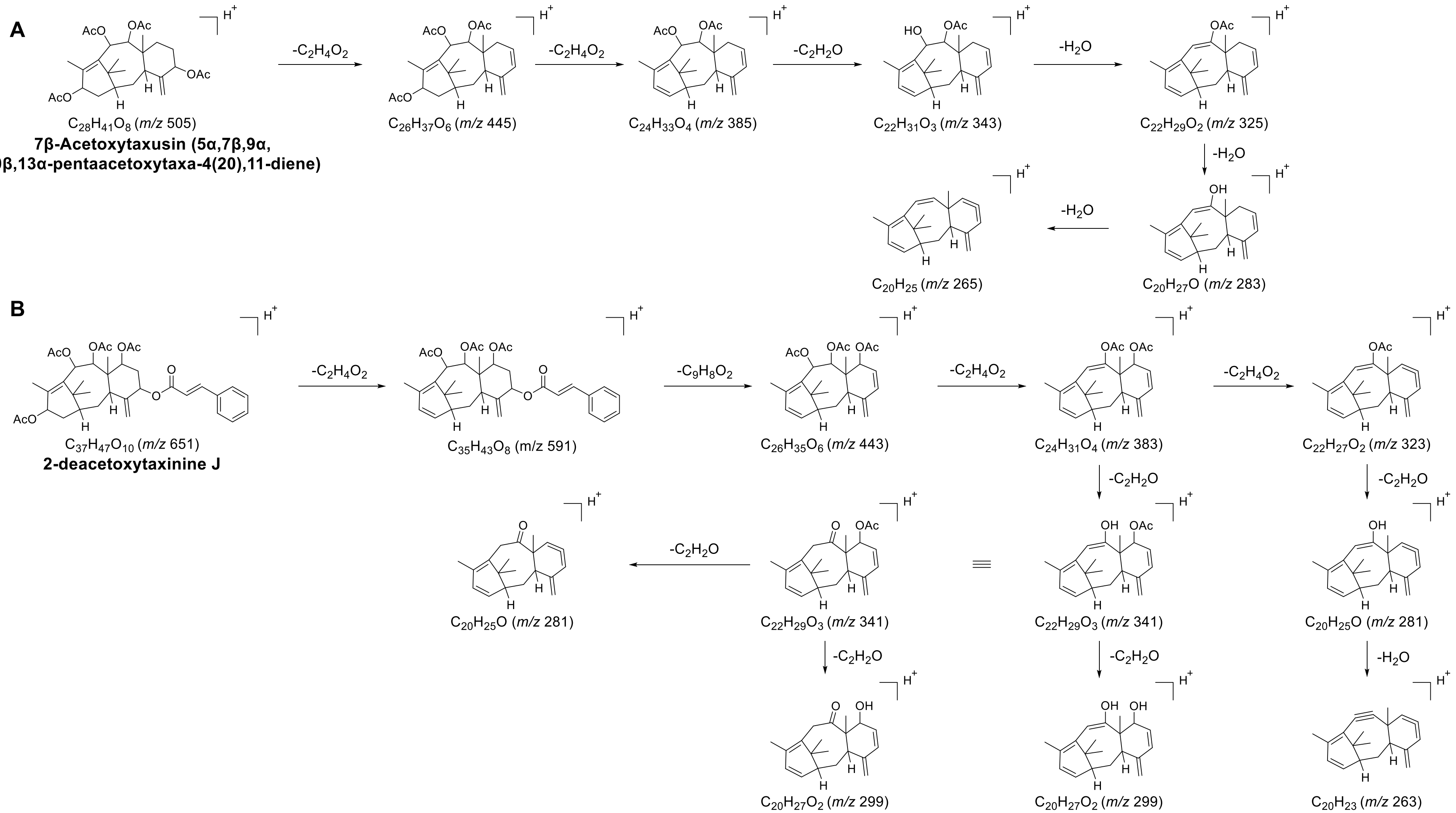

Figure S26. Proposal characteristic ESI-MS/MS fragmentation pathway of taxane-type diterpenoids. (A) 7*β*-Acetoxytaxusin (5*α*,7*β*,9*α*,10*β*,13*α*-pentaacetoxytaxa-4(20),11-diene) and (B) 2-deacetoxytaxinine J.

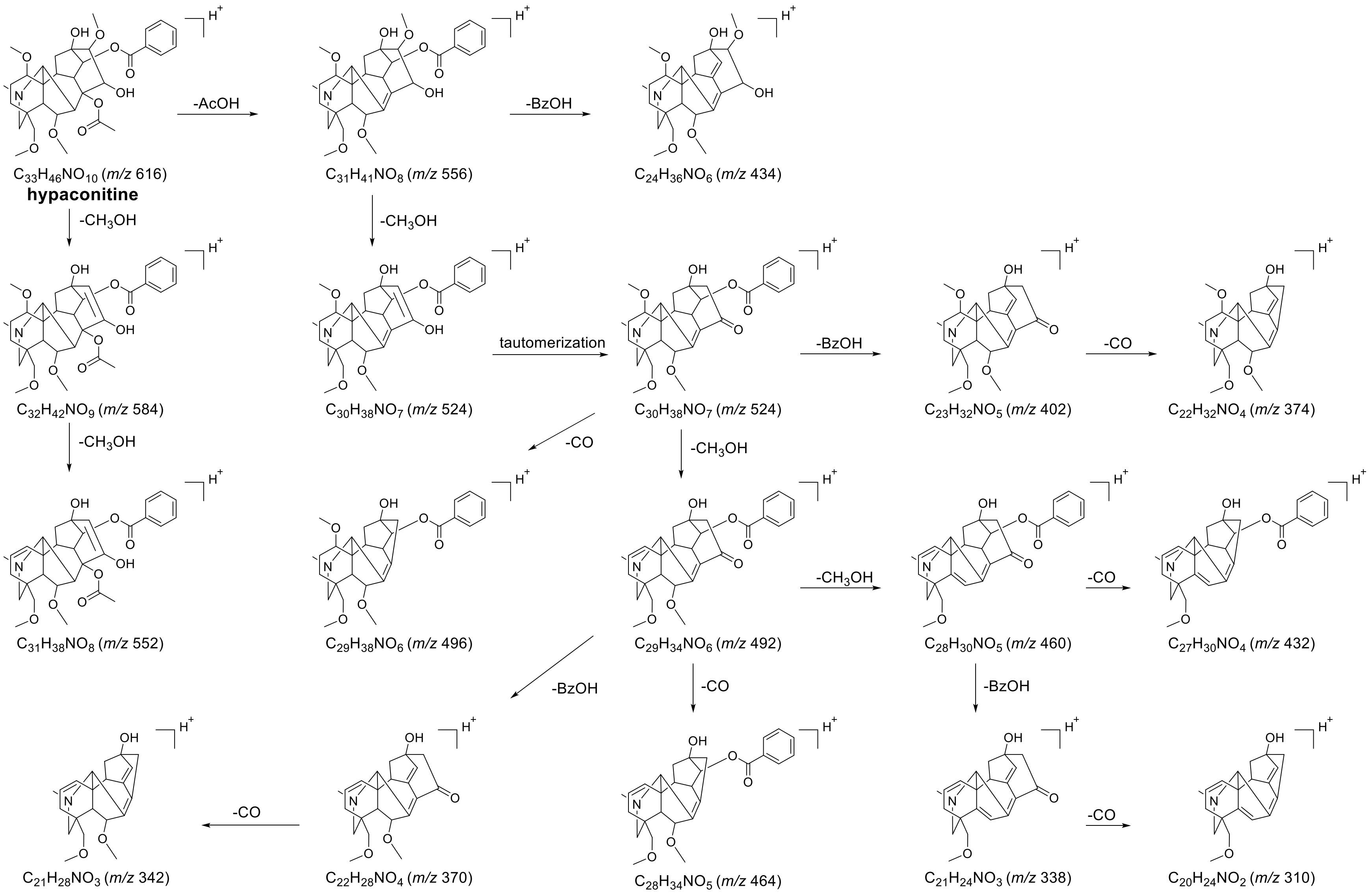

Figure S27. Proposal characteristic ESI-MS/MS fragmentation pathway of hypaconitine.

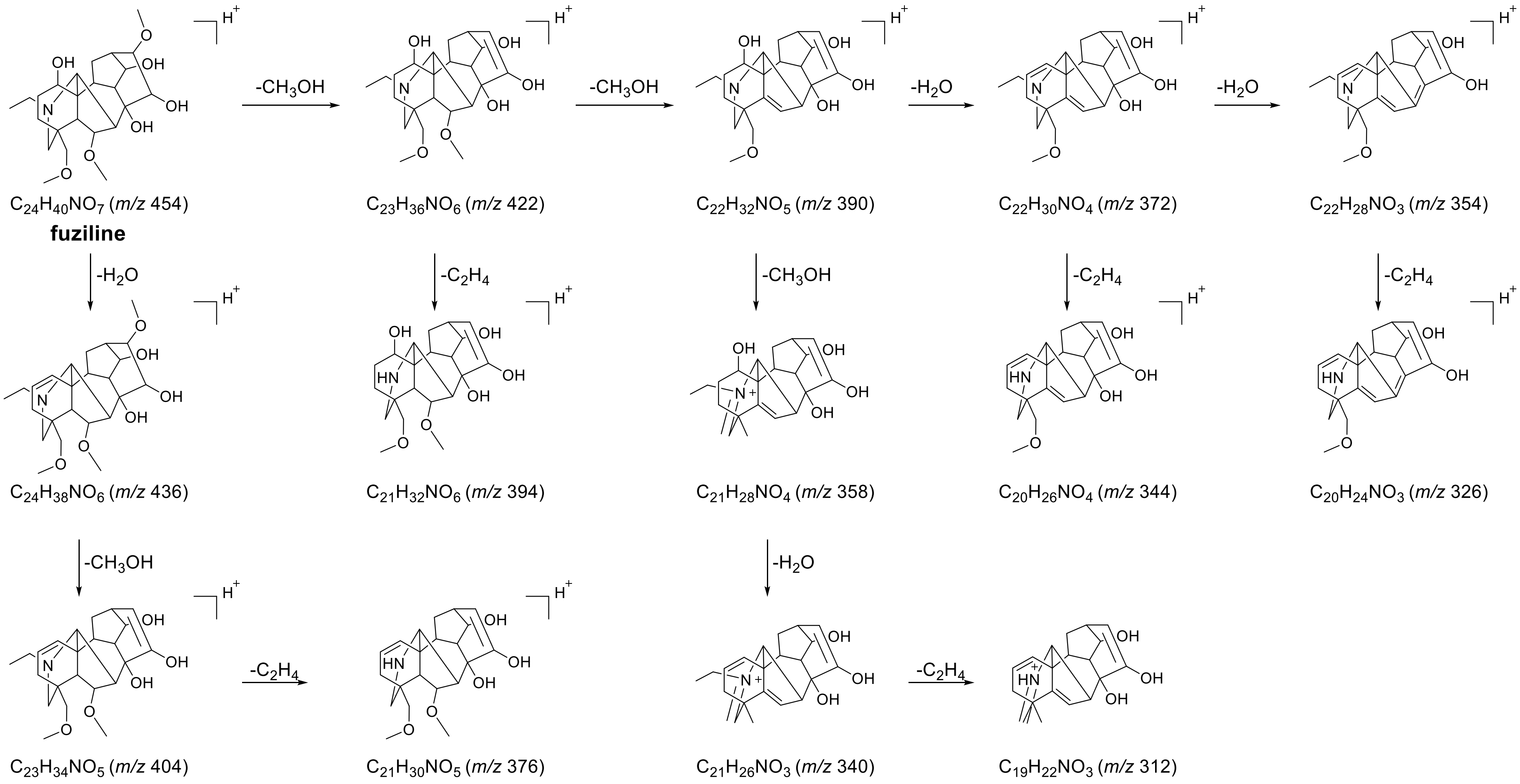

Figure S28. Proposal characteristic ESI-MS/MS fragmentation pathway of fuziline.

NMR data and spectra of compound 391 and 33

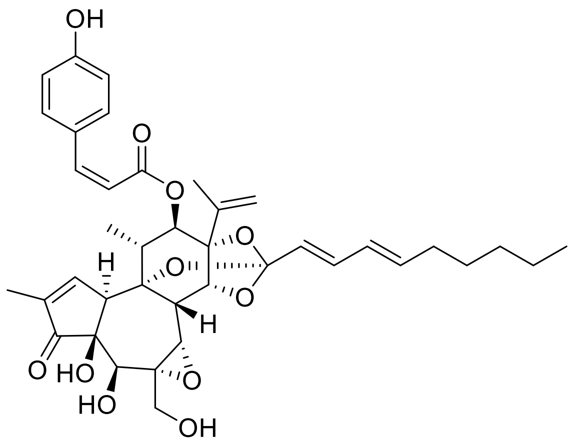

12-(*Z*)-Coumaroyloxy-6,7-epoxy-5-hydroxyresiniferonol-9,13,14-ortho-(2*E*,4*E*)-decadienoate (**391**): colorless solid; ^{1}H-NMR (500 MHz, $CDCl_3$) *d* 7.57 (1H, m), 7.15 (2H, d, 8.4), 7.05 (1H, d, 12.2), 6.89 (2H, d, 8.4), 6.57 (1H, dd, 15.4, 10.9), 5.78 (1H, d, 12.2), 5.99 (1H, dd, 15.2, 10.9), 5.80 (1H, dt, 15.2, 7.2), 5.56 (1H, d, 15.4), 4.92 (1H, brs), 4.91(1H, s), 4.74 (1H, brs), 4.40 (1H, d, 12.6), 4.27 (1H, s), 4.23 (1H, d, 2.5), 3.73 (1H, t, 2.6), 3.36 (1H, d, 12.6), 3.23 (1H, s), 2.88 (1H, d, 2.5), 2.31 (1H, q, 7.4), 2.06 (2H, q, 7.2), 1.78 (3H, m), 1.69 (3H, brs), 1.35 (2H, quin, 7.2), 1.28 (3H, d, 7.2), 1.28 (2H, m), 1.25 (2H, m), 0.86 (3H, t, 7.0) (**Figure S29**); ^{13}C-NMR (125 MHz, $CDCl_3$) *d* 209.6, 165.9, 161.3, 157.2, 144.6, 142.7, 139.2, 136.6, 134.9, 130.7, 128.6, 127.7, 122.3, 119.4, 116.6, 115.0, 113.7, 83.7, 79.9, 79.1, 77.9, 74.1, 72.1, 68.2, 66.3, 58.6, 47.8, 44.5, 35.8, 32.6, 31.3, 28.7, 22.5, 18.8, 18.4, 14.0, 9.8 (**Figure S30**).

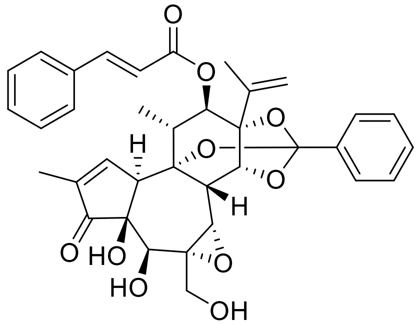

Gnidicin (**33**): colorless solid; ^{1}H-NMR (500 MHz, $CDCl_3$) *d* 7.71 (2H, dd, 7.7, 2.0), 7.64 (1H, d, 15.7), 7.58 (1H, dq, 2.6, 1.5), 7.53 (2H, dd, 7.4, 2.3), 7.39 (4H, m), 7.38 (2H, m), 6.38 (1H, d, 15.7), 5.17 (1H, s), 5.04 (1H, br s), 5.03 (1H, br s), 4.94 (1H, d, 2.6), 4.26 (2H, m), 3.92 (2H, m), 3.67 (1H, d, 2.6), 3.63 (1H, br s), 2.55 (1H, q, 7.2), 1.89 (3H, br s), 1.77 (3H, dd, 2.9, 1.5), 1.40 (3H, d, 7.2) (**Figure S31**); ^{13}C-NMR (125 MHz, $CDCl_3$) *d* 209.4, 165.8, 160.4, 146.0, 142.9, 137.0, 135.3, 134.1, 130.7, 129.7, 128.9, 128.3, 128.1, 126.0, 117.9, 117.3, 113.8, 84.3, 80.7, 78.6, 78.6, 72.2, 65.4, 64.5, 60.5, 47.6, 44.2, 35.7, 18.9, 18.3, 9.9 (**Figure S32**).

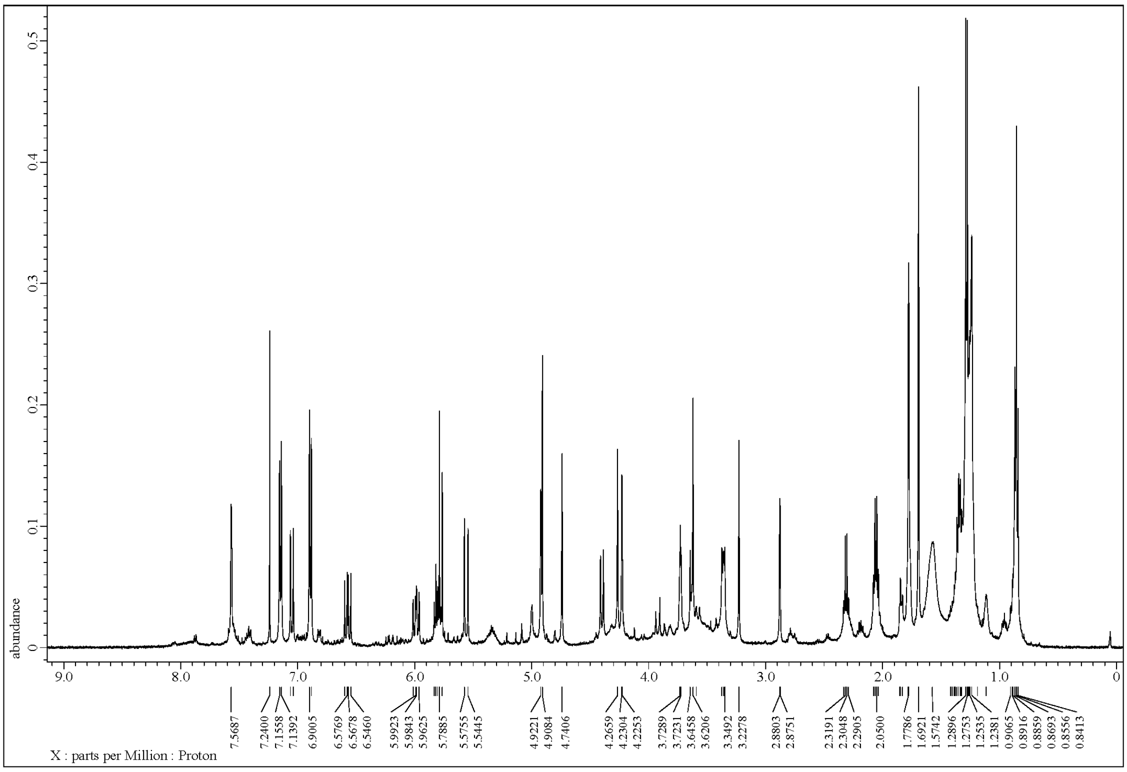

Figure S29. **^{1}H-NMR spectrum of 12-(*Z*)-coumaroyloxy-6,7-epoxy-5-hydroxyresiniferonol-9,13,14-ortho-(2*E*,4*E*)-decadienoate (391).**

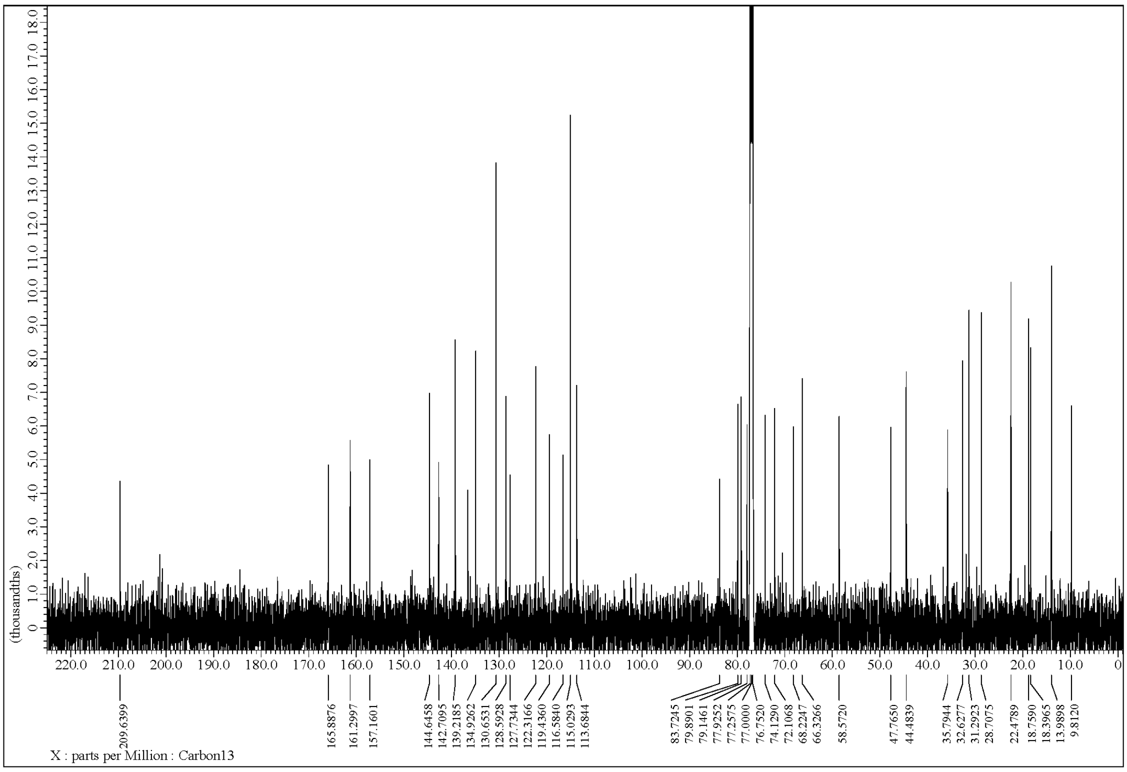

Figure S30. **^{13}C-NMR spectrum of 12-(*Z*)-coumaroyloxy-6,7-epoxy-5-hydroxyresiniferonol-9,13,14-ortho-(2*E*,4*E*)-decadienoate (391).**

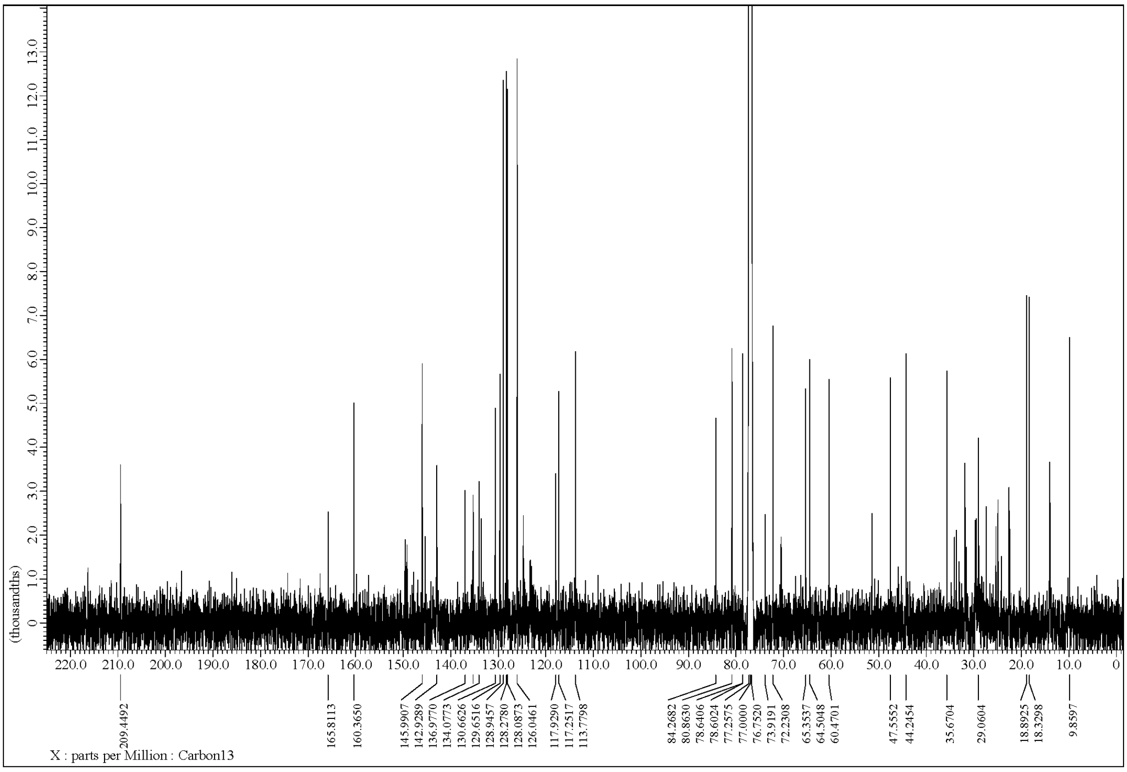

Figure S31. **^{1}H-NMR spectrum of gnidicin (33).**

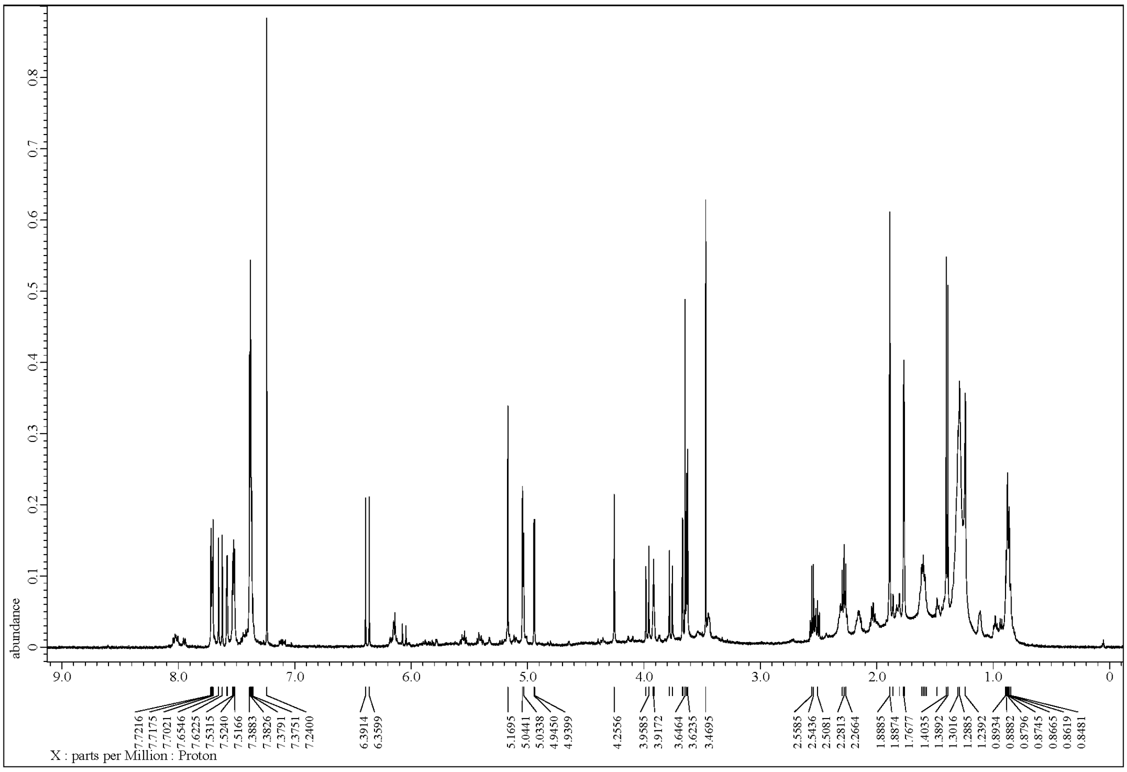

Figure S32. **^{13}C-NMR spectrum of gnidicin (33).**

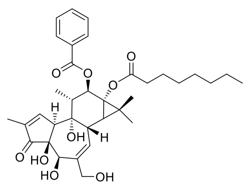

stelleracin C

Chemical Formula: $C_{35}H_{46}O_{9}$
Molecular Weight: 610.7440

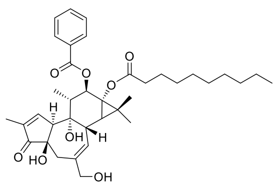

stellerarin

Chemical Formula: $C_{37}H_{50}O_{8}$
Molecular Weight: 622.7990

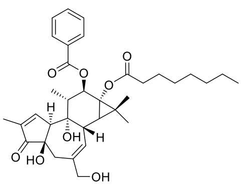

12-*O*-benzoylphorbol 13-octanoate

Chemical Formula: $C_{35}H_{46}O_{8}$
Molecular Weight: 594.7450

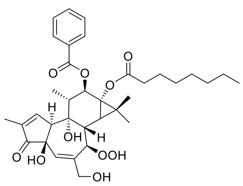

stelleracin E

Chemical Formula: $C_{35}H_{46}O_{10}$
Molecular Weight: 626.7430

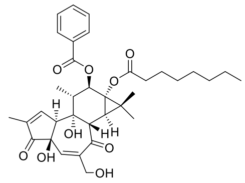

dapholosericin A

Chemical Formula: $C_{35}H_{44}O_{9}$
Molecular Weight: 608.7280

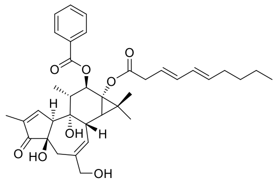

wikstroemia factor C$_1$

Chemical Formula: $C_{37}H_{46}O_{8}$
Molecular Weight: 618.7670

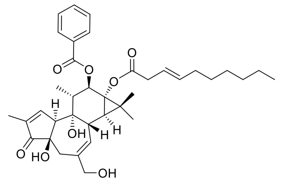

12-*O*-benzoyl-13-*O*-(3*E*)-decenoylphorbol

Chemical Formula: $C_{37}H_{48}O_{8}$
Molecular Weight: 620.78

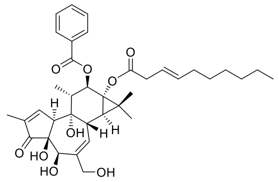

12-*O*-benzoyl-13-*O*-(3*E*)-decenoyl-5*β*-hydroxyphorbol

Chemical Formula: $C_{37}H_{48}O_{9}$
Molecular Weight: 636.78

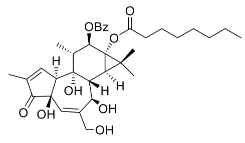

wikstrocin A

Chemical Formula: $C_{35}H_{46}O_{9}$
Molecular Weight: 610.7440

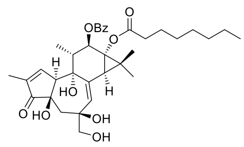

wikstrocin B

Chemical Formula: $C_{35}H_{46}O_{9}$
Molecular Weight: 610.7440

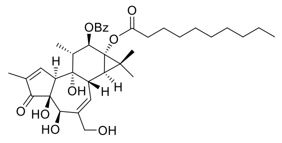

12-*O*-benzoyl-13-*O*-decanoyl-5*β*-hydroxyphorbol

Chemical Formula: $C_{37}H_{50}O_{9}$
Molecular Weight: 638.7980

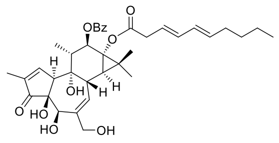

12-*O*-benzoyl-13-*O*-(3*E*,5*E*)-decadienoyl-5*β*-hydroxyphorbol

Chemical Formula: $C_{37}H_{46}O_{9}$
Molecular Weight: 634.77

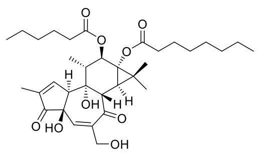

wikstrocin C

Chemical Formula: $C_{34}H_{50}O_{9}$
Molecular Weight: 602.7650

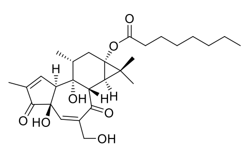

wikstrocin D

Chemical Formula: $C_{28}H_{40}O_{7}$
Molecular Weight: 488.6210

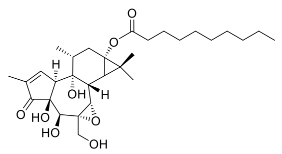

6*α*,7*α*-epoxy-5*β*-hydroxy-12-deoxyphorbol-13-decanoate

Chemical Formula: $C_{30}H_{46}O_{8}$
Molecular Weight: 534.6900

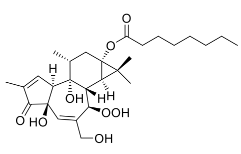

wikstrocin E

Chemical Formula: $C_{28}H_{42}O_{8}$
Molecular Weight: 506.6360

Figure S33. **Structures of 16 tigliane-type diterpenoids.**

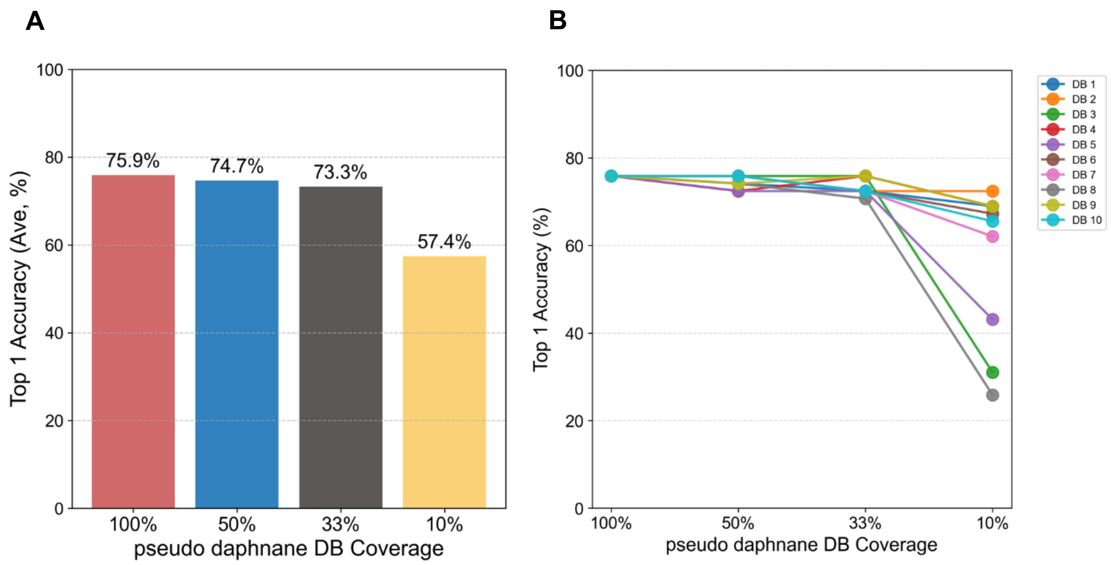

Figure S34. **Impact of candidate database coverage on Top-1 identification accuracy.** (A) average Top-1 accuracy over 10 pseudo-daphnane databases at each coverage level (100%, 50%, 33%, 10%).(B) Top-1 accuracy of individual database variants (DB1–DB10) at each coverage level.

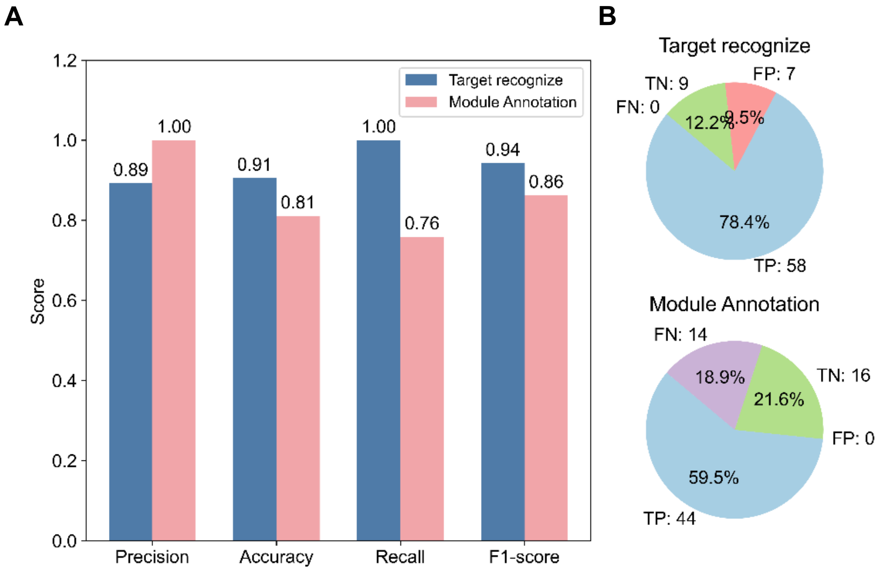

Figure S35. **Performance Evaluation of the CNPs-MFSA in Daphnane-Type Structure Annotation.** (A) Top-1 daphnane-type structures annotation performance (Precision, Accuracy, Recall, F1-score) by the Target Recognize module of CNPs-MFSA on a mixed dataset of 28 normal daphnanes, 30 macrocyclic orthoesters, and 16 tigliane-type diterpenoids. (B) Pie chart of true positives (TP), false positives (FP), and false negatives (FN) in structure annotations by CNPs-MFSA.

Table S1. Compounds Information of 58 in-house Daphnane-type Diterpenoids.

No.	Compound Name	Type[a]	Molecular Formula	Molecular Weight	Precursor Ion (+)	Adduct Ion (+)	Rt (min,+)
**DA01**	daphnetoxin	D	$C_{27}H_{30}O_8$	482.1914	483.2007	$[M+H]^+$	4.50
**DA02**	daphnepedunin J	D	$C_{29}H_{42}O_8$	518.2880	519.2948	$[M+H]^+$	10.15
**DA03**	excoecariatoxin	D	$C_{30}H_{40}O_8$	528.2723	529.2795	$[M+H]^+$	9.50
**DA04**	simplexin	D	$C_{30}H_{44}O_8$	532.3036	533.3104	$[M+H]^+$	11.60
**DA05**	daphnepedunin I	D	$C_{31}H_{46}O_8$	546.3193	547.3266	$[M+H]^+$	12.70
**DA06**	huratoxin	D	$C_{34}H_{48}O_8$	584.3349	585.3413	$[M+H]^+$	13.73
**DA07**	genkwanine N	D	$C_{34}H_{38}O_9$	590.2516	591.2588	$[M+H]^+$	8.38
**DA08**	genkwanine M	D	$C_{34}H_{38}O_9$	590.2516	591.2590	$[M+H]^+$	8.75
**DA09**	wikstrotoxin A	D	$C_{35}H_{50}O_8$	598.3506	599.3564	$[M+H]^+$	14.52
**DA10**	genkwadaphnin	D	$C_{34}H_{34}O_{10}$	602.2152	603.2209	$[M+H]^+$	6.68
**DA11**	wikstroelide M	D	$C_{34}H_{50}O_9$	602.3455	603.2209	$[M+H]^+$	8.72
**DA12**	genkwanine H	D	$C_{34}H_{40}O_{10}$	608.2621	609.2678	$[M+H]^+$	8.32
**DA13**	wikstroelide H	D	$C_{34}H_{46}O_{10}$	614.3091	615.3149	$[M+H]^+$	11.17
**DA14**	stelleralide K	D	$C_{34}H_{48}O_{11}$	632.3197	633.3262	$[M+H]^+$	7.43
**DA15**	wikstroelide A	D	$C_{36}H_{50}O_{10}$	642.3404	643.3462	$[M+H]^+$	13.31
**DA16**	yuanhuajine	D	$C_{37}H_{42}O_{10}$	646.2778	647.2842	$[M+H]^+$	9.94
**DA17**	gniditrin	D	$C_{37}H_{42}O_{10}$	646.2778	647.2843	$[M+H]^+$	9.59
**DA18**	yuanhuacine	D	$C_{37}H_{44}O_{10}$	648.2934	649.3004	$[M+H]^+$	10.83
**DA19**	daphneodorin H	D	$C_{37}H_{48}O_{10}$	652.3247	653.3314	$[M+H]^+$	9.15
**DA20**	mezereine	D	$C_{38}H_{38}O_{10}$	654.2465	655.2537	$[M+H]^+$	8.64

**DA21**	12-*O*-(*E*)-cinnamoyl-9,13,14-ortho-(2*E*,4*E*,6*E*)-decatrienylidyne-5β,12β-dihydroxyresiniferonol-6α,7α-oxide	D	$C_{39}H_{44}O_{10}$	672.2934	673.3003	$[M+H]^+$	10.70
**DA22**	12-*O*-(*E*)-cinnamoyl-9,13,14-ortho-(2*E*,4*E*)-decadienylidyne-5β,12β-dihydroxyresiniferonol-6α,7α-oxide	D	$C_{39}H_{46}O_{10}$	674.3091	675.3156	$[M+H]^+$	11.49
**DA23**	daphneodorin E	D	$C_{39}H_{44}O_{11}$	688.2884	689.2947	$[M+H]^+$	9.18
**DA24**	daphneodorin D	D	$C_{39}H_{46}O_{11}$	690.3040	691.3102	$[M+H]^+$	9.97
**DA25**	daphneodorin G	D	$C_{39}H_{48}O_{11}$	692.3197	693.3252	$[M+H]^+$	7.99
**DA26**	acutilobin C	D	$C_{40}H_{46}O_{12}$	718.2989	719.3053	$[M+H]^+$	9.01
**DA27**	acutilobin D	D	$C_{40}H_{48}O_{12}$	720.3146	721.3205	$[M+H]^+$	9.77
**DA28**	daphneodorin F	D	$C_{40}H_{48}O_{13}$	736.3095	737.3159	$[M+H]^+$	5.40
**DA29**	daphnepedunin C	MD	$C_{29}H_{42}O_{8}$	518.2880	519.2952	$[M+H]^+$	8.36
**DA30**	pimelea factor S_7	MD	$C_{30}H_{44}O_{8}$	532.3036	533.3105	$[M+H]^+$	9.22
**DA31**	pimelea factor S_6	MD	$C_{30}H_{44}O_{8}$	532.3036	533.3107	$[M+H]^+$	9.21
**DA32**	pimelotide A	MD	$C_{30}H_{42}O_{9}$	546.2829	547.2891	$[M+H]^+$	9.31
**DA33**	pimelotide C	MD	$C_{30}H_{42}O_{9}$	546.2829	547.2899	$[M+H]^+$	8.32
**DA34**	daphnepedunin B	MD	$C_{30}H_{44}O_{9}$	548.2985	549.3059	$[M+H]^+$	5.98
**DA35**	daphnepedunin A	MD	$C_{30}H_{44}O_{9}$	548.2985	549.3057	$[M+H]^+$	5.58
**DA36**	daphnepedunin G	MD	$C_{30}H_{44}O_{9}$	548.2985	549.3054	$[M+H]^+$	5.36
**DA37**	daphnepedunin H	MD	$C_{30}H_{44}O_{9}$	548.2985	549.3054	$[M+H]^+$	5.42

**DA38**	wikstromacrin	MD	$C_{37}H_{50}O_9$	638.3455	639.3519	$[M+H]^+$	12.50
**DA39**	pimela factor P_2	MD	$C_{37}H_{50}O_9$	638.3455	639.3522	$[M+H]^+$	13.30
**DA40**	edgeworthianin D	MD	$C_{36}H_{52}O_{10}$	644.3560	645.3622	$[M+H]^+$	10.96
**DA41**	wikstroelide F	MD	$C_{37}H_{48}O_{10}$	652.3247	653.3320	$[M+H]^+$	10.46
**DA42**	edgeworthianin A	MD	$C_{36}H_{50}O_{11}$	658.3353	659.3413	$[M+H]^+$	10.93
**DA43**	stelleralide C	MD	$C_{37}H_{46}O_{11}$	666.3040	667.3110	$[M+H]^+$	10.68
**DA44**	daphnepedunin E	MD	$C_{37}H_{46}O_{11}$	666.3040	667.3114	$[M+H]^+$	10.50
**DA45**	daphnepedunin D	MD	$C_{37}H_{48}O_{11}$	668.3197	669.3265	$[M+H]^+$	5.51
**DA46**	edgeworthianin F	MD	$C_{38}H_{54}O_{12}$	702.3615	703.3682	$[M+H]^+$	10.24
**DA47**	edgeworthianin E	MD	$C_{40}H_{58}O_{12}$	730.3928	731.3990	$[M+H]^+$	11.04
**DA48**	edgeworthianin B	MD	$C_{40}H_{56}O_{13}$	744.3721	745.3771	$[M+H]^+$	11.65
**DA49**	edgeworthianin G	MD	$C_{41}H_{58}O_{13}$	758.3877	759.3942	$[M+H]^+$	12.31
**DA50**	stelleralide H	MD	$C_{44}H_{54}O_{12}$	774.3615	775.3675	$[M+H]^+$	9.57
**DA51**	gnidimacrin	MD	$C_{44}H_{54}O_{12}$	774.3615	775.3684	$[M+H]^+$	10.24
**DA52**	edgeworthianin C	MD	$C_{43}H_{54}O_{13}$	778.3564	779.3620	$[M+H]^+$	12.13
**DA53**	daphnepedunin F	MD	$C_{44}H_{50}O_{13}$	786.3251	787.3316	$[M+H]^+$	10.94
**DA54**	stelleralide F	MD	$C_{46}H_{56}O_{14}$	832.3670	833.3741	$[M+H]^+$	7.98
**DA55**	stelleralide G	MD	$C_{51}H_{58}O_{14}$	894.3827	895.3884	$[M+H]^+$	10.15
**DA56**	daphneodorin A	MD	$C_{53}H_{60}O_{16}$	952.3881	953.3942	$[M+H]^+$	8.95
**DA57**	daphneodorin B	MD	$C_{55}H_{62}O_{18}$	1010.3936	1011.3989	$[M+H]^+$	8.18
**DA58**	daphneodorin C	MD	$C_{55}H_{62}O_{18}$	1010.3936	1011.3993	$[M+H]^+$	7.61

[a] D: normal daphnane-type diterpenoids, MD: macrocyclic daphnane orthoesters.

Table S2. ^{1}H (500 MHz) and ^{13}C (125 MHz) NMR Spectroscopic Data of DA04a– DA04c ($CDCl_3$).

no.	DA4a		DA4b		DA4c	
	δ_H (J in Hz)	δ_C	δ_H (J in Hz)	δ_C	δ_H (J in Hz)	δ_C
1	5.61 (1H, brd, 1.6)	128.1	1.60 (1H, d, 12.9)<br>2.32 (1H, quin, 6.3)	33.4	1.81 (1H, m)<br>1.51 (1H, m)	33.7
2		135.6	2.25 (1H, quin, 6.8)	42.9	1.80 (1H, m)	35.0
3	4.40 (1H, s)	83.5		221.0	3.50 (1H, s)	93.3
4		78.3		79.0		79.5
5	4.05 (1H, s)	75.6	4.07 (1H, s)	71.7	4.34 (1H, d, 2.9)	72.1
6		61.0		60.3		59.9
7	3.35 (1H, s)	64.0	3.41 (1H, s)	64.6	3.37 (1H, s)	64.5
8	2.80 (1H, d, 2.2)	36.9	2.72 (1H, d, 2.6)	36.8	2.81 (1H, d, 2.5)	36.5
9		79.7		79.4		79.5
10	3.40 (1H, brd, 1.6)	52.2	2.82 (1H, dd, 13.3, 5.9)	44.7	2.59 (1H, dd, 13.2, 6.0)	46.1
11	2.38 (1H, quin, 7.4)	35.0	2.32 (1H, quin, 6.3)	35.3	2.33 (1H, quin, 7.2)	34.9
12	1.60 (1H, d, 14.2)<br>2.16 (1H, dd, 14.2, 8.6)	37.1	1.55 (1H, d, 14.1)<br>1.81 (1H, m)	34.0	1.55 (1H, m)<br>1.82 (1H, m)	34.1
13		83.8		85.0		85.1
14	4.29 (1H, d, 2.6)	82.2	4.27 (1H, d, 2.9)	79.4	4.34 (1H, d, 2.9)	79.1
15		146.7	1.84 (1H, m)	34.8		
16	4.86 (1H, t, 1.5)<br>4.99 (1H, s)	110.9	0.9 (3H, dd, 8.3, 7.2)	17.1[a]	0.9 (3H, t, 7.3)	17.2[a]
17	1.75 (3H, s)	19.0	0.9 (3H, dd, 8.3, 7.2)	17.3[a]	0.9 (3H, t, 7.0)	17.3[a]
18	1.18 (3H, d, 6.9)	21.1	1.19 (3H, d, 6.9)	20.7	1.15 (3H, d, 6.9)	20.6
19	1.71 (3H, t, 1.2)	13.4	1.12 (3H, d, 6.3)	12.5	1.03 (3H, d, 6.3)	16.2
20	3.69 (1H, d, 12.3)<br>3.97 (1H, d, 12.3)	65.5	3.79 (2H, s)	65.4	3.68 (1H, dd, 12.2, 6.3)<br>3.88 (1H, dd, 12.2, 6.3)	66.3
1'		119.4		119.0		118.9
2'	1.90 (2H, m)	35.0	1.90 (2H, m)	35.0	1.90 (2H, m)	35.0
3'	1.56 (2H, m)	23.5	1.56 (2H, m)	23.5	1.56 (2H, m)	23.5
4'	1.32 (2H, m)	29.6	1.32 (2H, m)	29.6	1.32 (2H, m)	29.6
5'	1.20-1.35 (2H, m)	29.6	1.20-1.35 (2H, m)	29.6	1.20-1.35 (2H, m)	29.6
6'	1.20-1.35 (2H, m)	29.5	1.20-1.35 (2H, m)	29.5	1.20-1.35 (2H, m)	29.5
7'	1.20-1.35 (2H, m)	29.3	1.20-1.35 (2H, m)	29.3	1.20-1.35 (2H, m)	29.3
8'	1.24 (2H, m)	31.9	1.24 (2H, m)	31.9	1.24 (2H, m)	31.9
9'	1.26 (2H, m)	22.7	1.26 (2H, m)	22.7	1.26 (2H, m)	22.7
10'	0.86 (3H, t, 7.0)	14.1	0.86 (3H, t, 6.9)	14.1	0.86 (3H, t, 6.9)	14.1

[a]Interchangeable resonances.

Table S3. **56 Plant Materials of Thymelaeaceae family.**

No.	Genus	Species	Part	Abbreviation	Origin
Thy01	*Daphne*	*D. tangutica*	aerial part	Dta-ap	China
Thy02	*Daphne*	*D. bholum*	whole plant	Dbh-w	China
Thy03	*Daphne*	*D. axillaris*	twig, leaf	Dax-tl	China
Thy04	*Daphne*	*D. grueningiana*	aerial part	Dgr-ap	China
Thy05	*Daphne*	*D. feddei*	whole plant	Dfe-w	China
Thy06	*Daphne*	*D. gemmata*	whole plant	Dge-w	China
Thy07	*Daphne*	*D. giraldii*	whole plant	Dgi-w	China
Thy08	*Daphne*	*D. acutiloba Rehd.*	twig, leaf	Dac-tl	China
Thy09	*Daphne*	*D. yunnanensis*	whole plant	Dyu-w	China
Thy10	*Daphne*	*D. aurantiaca*	twig, leaf	Dau-tl	China
Thy11	*Daphne*	*D. retusa*	twig, leaf	Dre-tl	China
Thy12	*Daphne*	*D. papyracea var. crassiuscula*	twig, leaf	Dpa-tl	China
Thy13	*Daphne*	*D. pedunculata*	whole plant	Dpe-w	China
Thy14	*Daphne*	*D. atrocaulis*	leaf, stem	Dat-ls	China
Thy15	*Daphne*	*D. kiusiana var. atrocaulis*	whole plant	Dki-w	China
Thy16	*Daphne*	*D. holoserica*	whole plant	Dho-w	China
Thy17	*Daphne*	*D. depauperata*	whole plant	Dde-w	China
Thy18	*Daphne*	*D. odora*	flower	Dod-fr	Japan
Thy19	*Daphne*	*D. odora*	bud	Dod-b	Japan
Thy20	*Daphne*	*D. odora*	leaf	Dod-l	Japan
Thy21	*Daphne*	*D. odora*	root	Dod-r	Japan
Thy22	*Daphne*	*D. odora*	stem	Dod-s	Japan
Thy23	*Daphne*	*D. jezoensis*	flower	Dje-fr	Japan
Thy24	*Daphne*	*D. jezoensis*	fruit	Dje-ft	Japan
Thy25	*Daphne*	*D. genkwa*	flower	Dge-fr	Japan
Thy26	*Daphne*	*D. pseudomezereum*	flower	Dps-fr	Japan
Thy27	*Daphne*	*D. pseudomezereum*	fruit	Dps-ft	Japan
Thy28	*Daphne*	*D. pontica*	flower	Dpo-fr	Turkey
Thy29	*Daphne*	*D. pontica*	leaf	Dpo-l	Turkey
Thy30	*Daphne*	*D. pontica*	stem	Dpo-s	Turkey
Thy31	*Wikstroemia*	*W. dolichantha*	whole plant	Wdo-w	China
Thy32	*Wikstroemia*	*W. lamatsoensis*	whole plant	Wla-w	China
Thy33	*Wikstroemia*	*W. scytophylla*	whole plant	Wsc-w	China
Thy34	*Wikstroemia*	*W. pilosa*	whole plant	Wpi-w	China
Thy35	*Wikstroemia*	*W. ligustrina*	whole plant	Wli-w	China
Thy36	*Wikstroemia*	*W. paniculata*	whole plant	Wpa-w	China
Thy37	*Wikstroemia*	*W. indica*	leaf	Win-l	China
Thy38	*Wikstroemia*	*W. indica*	stem	Win-s	China
Thy39	*Wikstroemia*	*W. capitato-racemosa*	whole plant	Wca-w	China
Thy40	*Wikstroemia*	*W. alternifolia*	whole plant	Wal-w	China
Thy41	*Wikstroemia*	*W. lichiangensis*	aerial part	Wli-ap	China
Thy42	*Wikstroemia*	*W. lichiangensis*	root	Wli-r	China
Thy43	*Wikstroemia*	*W. stenophylla*	whole plant	Wst-w	China
Thy44	*Wikstroemia*	*W. nutans*	whole plant	Wnu-w	China
Thy45	*Wikstroemia*	*W. lungtzeensis*	whole plant	Wlu-w	China
Thy46	*Wikstroemia*	*W. micrantha*	twig, leaf	Wmi-tl	China

Thy47	*Wikstroemia*	*W. delavayi*	whole plant	Wde-w	China
Thy48	*Wikstroemia*	*W. trichotoma*	twig, leaf	Wtr-tl	China
Thy49	*Edgeworthia*	*E. gardneri*	whole plant	Ega-w	China
Thy50	*Edgeworthia*	*E. albiflora*	whole plant	Eal-w	China
Thy51	*Edgeworthia*	*E. chrysantha*	fruit	Ech-ft	Japan
Thy52	*Edgeworthia*	*E. chrysantha*	bud	Ech-b	Japan
Thy53	*Edgeworthia*	*E. chrysantha*	leaf	Ech-l	Japan
Thy54	*Edgeworthia*	*E. chrysantha*	stem	Ech-s	Japan
Thy55	*Edgeworthia*	*E. chrysantha*	flower	Ech-fr	China
Thy56	*Stellera*	*S. chamaejasme L.*	root	Sch-r	China

Table S4. **Skeleton substructure of type 1−4.**

Type 1	Structure	Type 2	Structure	Type 3	Structure	Type 4	Structure
A1	1,2-en-3-one	B1	5-ol-6,7-epoxy	C1	9,13,14-orthoester	M1	C9 macrocyclic ring
A2	1,2-dihydro-3-one	B2	5-ol-6,7-diol	C2	12-ol and 9,13,14-orthoester	M2	C10 macrocyclic ring without acylation
A3	1,2-en-3-ol	B3	5-ol-6,7-ene	C3	9,13,14-triol	M3	C10 macrocyclic ring, 2′-ol
A4	1,2-dihydro-3-ol	B4	5-dehydro-6,7-epoxy	C4	12-ol and 9,13,14-triol	M4	C10 macrocyclic ring, 2′-ol-7′-ol
A5	1,10-en-3-one	B5	5-dehydro-6,7-diol	C5	9,13,14-orthoester, 18-ol	M5	C10 macrocyclic ring, 2′-ol-6′-ol-7′-ol
A6	1-alkyl-3-one	B6	5-dehydro-6,7-ene	C6	9,13,14-orthoester, 12-ol-18-ol	M6	C10 macrocyclic ring, 2′-ol-6′-ol-7′-ol
A7	1-alkyl-2-ol-3-one	B7	5-ol-4,7-epoxy			M7	C14 macrocyclic ring, 5′,6′-ene
A8	1-alkyl-3-ol	B8	5-ol-4,6-epoxy			M8	C16 macrocyclic ring
A9	1-alkyl-2-ol-3-ol					M9	C16 macrocyclic ring, 15′-ol
A10	3,4-seco						
A11	bicyclo[2.2.1]heptane ring						

Table S5. **Public daphnane-related datasets from GNPS.**

No.	Dataset	Data Name	Family	Plant Name	Part	Instrument
1	MSV000087728	VGF141_A07	Enkleia	*Enkleia malaccensis*	roots	Q Exactive Focus
2	MSV000087728	VGF141_G06	Enkleia	*Enkleia malaccensis*	leaves	Q Exactive Focus
3	MSV000087728	VGF141_H06	Enkleia	*Enkleia malaccensis*	woody stems	Q Exactive Focus
4	MSV000087728	VGF147_A05	Aquilaria	*Aquilaria agallocha*	roots	Q Exactive Focus
5	MSV000087728	VGF147_B03	Aquilaria	*Aquilaria agallocha*	leaves	Q Exactive Focus
6	MSV000097043	Eup50_01	Euphorbia	*Euphorbia kansui*	tuber	Orbitrap Exploris 120
7	MSV000097043	Eup50_02	Euphorbia	*Euphorbia lathyris*	seed	Orbitrap Exploris 120
8	MSV000097043	EupE01	Euphorbia	*Euphorbia kansui*	tuber	Orbitrap Exploris 120
9	MSV000097043	EupE02	Euphorbia	*Euphorbia lathyris*	seed	Orbitrap Exploris 120
10	MSV000097043	EupW_01	Euphorbia	*Euphorbia kansui*	tuber	Orbitrap Exploris 120
11	MSV000097043	EupW_02	Euphorbia	*Euphorbia lathyris*	seed	Orbitrap Exploris 120

Table S6. **Information on plants containing different classes of compounds.**

No.	Data source	Plant Name	Abbreviation	Data Name	Instrument
1	Crude drug extraction	Aconiti radix processa	ARP	fuzi	Q Exactive Orbitrap
2	Crude drug extraction	Citri unshiu pericarpium	CUP	chenpi	Q Exactive Orbitrap
3	Crude drug extraction	Phellodendri cortex	PC	huangbai	Q Exactive Orbitrap
4	Crude drug extraction	Aurantii fructus immaturus	AFI	zhishi	Q Exactive Orbitrap
5	Crude drug extraction	Citri Unshiu Pericarpium Immaturus	CUPI	qingpi	Q Exactive Orbitrap
6	MSV000087217	*Taxus brevifolia*	TAX	NCBITaxon:46220	Q Exactive

Table S7. **Target Recognize results for daphnanes annotation.**

	Predicted Positive	Predicted Negative
Actual Positive	TP: 58	FP: 7
Actual Negative	TN: 9	FN: 0

Table S8. **Module Annotation results for daphnanes annotation.**

	Predicted Positive	Predicted Negative
Actual Positive	TP: 44	FP: 0
Actual Negative	TN: 16	FN: 14

Table S9. **Summary of evaluation metrics for structure annotation.**

Metric	Target recognize	Module Annotation
Precision	0.8923	1
Recall	1	0.7586
F1-score	0.943	0.8627
FPR	0.4375	0
Accuracy	0.9054	0.8108

Other Supplementary Materials for this manuscript include the following:
Table S10: pseudo-daphnane library containing aglycone and feature product ions
Table S11: pseudo-daphnane library containing substituents and feature product ions
Table S12: accuracy evaluation of CNPs-MFSA against SIRIUS, MS-FINDER, MetFrag
Table S13: daphnane annotation result for 56 Thymeleaceae extracts
Table S14: daphnane annotation result for 11 GNPS public Thymeleaceae extracts

LC-MS dataset of standards and extracts.
Zenodo: https://doi.org/10.5281/zenodo.15503081
GNPS MassIVE: MSV000098518
Code: [Github]: https://github.com/MiZhang47/CNPs-MFSA.git
[Dryad]: https://doi.org/10.5061/dryad.gqnk98szp

External Code Document: CNPs-MFSA The full implementation of the CNPs-MFSA application, including the GUI version, web-based version, and associated resources (e.g., input/output examples, formula database generation, pseudo-databases, and user manual), is available in the provided GitHub repository.
CNPs_MFSA_Web.py
Main Web Interface of CNPs-MFSA Tool
This script serves as the primary Python implementation for the Dash-based graphical user interface (GUI) of the CNPs-MFSA application. It integrates file upload, parameter control, and result display for automated structural annotation of complex natural products.

User Manual of CNPs-MFSA.pdf
User Manual for the CNPs-MFSA Tool
Detailed instruction manual outlining how to operate the CNPs-MFSA web tool, including guidance on file formats, input preparation, and interpretation of output.

pseudo daphnane DB (200w).zip
Pseudo-CNPs Library for Daphnane Annotation (200k entries)
A zip archive containing a pseudo-daphnane compound library used for candidate structure matching in the annotation pipeline. Generated via modular assembly.

known daphnane library (215).zip
Validated Daphnane Compound Library
A reference library of 215 experimentally validated daphnane-type diterpenoids used for benchmarking annotation accuracy.

Sensitive Analysis Dataset.zip
Sensitivity Analysis Dataset for Annotation Robustness
Includes perturbed input datasets used for sensitivity testing of the CNPs-MFSA strategy, demonstrating robustness under different input configurations.

pseudo aconitine DB
aconitine target recognize and feature ion.txt
Target Recognition Rules and Feature Ion Definitions for Aconitine-type Alkaloids
Defines rules and key fragment ions used to recognize aconitine-type compounds in mass spectrometry data.

pseudo-aconitine library.xlsx
Pseudo-Compound Library of Aconitine Analogs
Excel spreadsheet listing pseudo-structures of aconitine-type alkaloids generated by modular fragmentation strategy.

pseudo obakunone DB
obakunone analogs-annotation rule.txt
Annotation Rules for Obakunone-type Triterpenoids
Describes structural rules and logic for annotating obakunone analogs based on MS/MS fragmentation characteristics.

obakunone analogs-feature ion labeling_conditions.txt
Feature Ion Labeling Conditions for Obakunone Analogs
Specifies conditions for assigning diagnostic fragment ions in obakunone-type compounds.

obakunone analogs-target recognize_conditions.txt
Target Recognition Conditions for Obakunone Analogs
Details logic rules and threshold settings for initial target recognition of obakunone-type compounds.

pseudo-obakunone library.xlsx
Pseudo-Compound Library of Obakunone Analogs
Contains combinatorially generated obakunone-like structures used for structure matching during annotation.
pseudo taxol DB
pseudo-taxol library.xlsx
Pseudo-Compound Library of Taxol Analogs

A curated pseudo-database of taxol (paclitaxel) analogs constructed via modular assembly.

taxol_target recognize_feature ion labeling.txt
Target Recognition and Feature Ion Definitions for Taxol-type Compounds
Lists major fragment ions and recognition rules used to identify taxol-related structures in experimental data.

input files (daphnane)
Acyl.csv
Acyl Substructure Definition File
Contains the list of acyl group substructures and corresponding formulas used for acyl module annotation in daphnane-type diterpenoids.

feature ion and neutral loss (daphnane).txt
Diagnostic Ion and Neutral Loss List for Daphnane Annotation
Enumerates key fragment ions and characteristic neutral losses for identifying daphnane-type compounds.

Positive Ion Formula.db
Positive Ion Formula Database for Daphnane Annotation
Stores positive ion adduct formulas and their corresponding masses used during precursor ion analysis.

Positive Ion Formula_NL.db
Positive Ion Neutral Loss Database for Daphnane Annotation
Contains known neutral losses associated with adduct ions to assist in structure assignment.

pseudo-D.zip
Pseudo-Library of Normal Daphnane-type Diterpenoids
Compressed file of pseudo-structures of normal daphnane compounds generated via modular combination.

pseudo-MD.zip
Pseudo-Library of Macrocyclic Daphnane-type Diterpenoids
Compressed file of macrocyclic daphnane analogs used for structural annotation.
input files (for CNPs)
Positive Ion Formula.db
Positive Ion Formula Database for General CNPs

Same as above, but tailored for non-daphnane CNP classes such as aconitines, taxanes, and limonoids.

Positive Ion Formula_NL.db
Positive Ion Neutral Loss Database for General CNPs
Neutral loss entries corresponding to precursor ion types across various CNP subclasses.
